# Leaves and fruits of *Bauhinia* (Leguminosae, Caesalpinioideae, Cercideae) from the Oligocene Ningming Formation of Guangxi, South China and their biogeographic implications

**DOI:** 10.1186/1471-2148-14-88

**Published:** 2014-04-24

**Authors:** Qi Wang, Zhuqiu Song, Yunfa Chen, Si Shen, Zhenyu Li

**Affiliations:** 1 State Key Laboratory of Systematic and Evolutionary Botany, Institute of Botany, Chinese Academy of Sciences, Beijing 100093, P.R. China; 2 Natural History Museum of Guangxi, Nanning 530012, P.R. China

**Keywords:** *Bauhinia*, Bifoliolate leaf, Bilobate leaf, Biogeography, Cercideae, *Cercis*, Eocene, Evolution, Fruits, Legumes, Leguminosae, Low latitude, Ningming Formation, Oligocene, Pulvinus, Tethys Seaway origin, Unifoliolate leaf

## Abstract

**Background:**

The pantropical genus *Bauhinia*, along with the northern temperate *Cercis* and several tropical genera, bear bilobate, bifoliolate, or sometimes unifoliolate leaves, which constitute the tribe Cercideae as sister to the rest of the family Leguminosae based on molecular phylogenetics. Hence, the fossil record of Cercideae is pivotal to understand the early evolution and biogeographic history of legumes.

**Results:**

Three fossil species of *Bauhinia* were described from the Oligocene Ningming Formation of Guangxi, South China. *Bauhinia ningmingensis* sp. nov. is characterized by its bifoliolate, pulvinate leaves bearing basal acrodromous primary veins and brochidodromous secondary veins. *B. cheniae* sp. nov. bears moderately or deeply bilobate, pulvinate leaves, with basal actinodromous primary veins and eucamptodromous secondary veins. *B. larsenii* D.X. Zhang et Y.F. Chen emend. possesses shallowly or moderately bilobate, pulvinate leaves bearing basal actinodromous primary veins and brochidodromous secondary veins, as well as elliptic, stipitate, non-winged, and oligo-seeded fruits. Meanwhile, previously reported *Bauhinia* fossils were reviewed, and those pre-Oligocene foliage across the world are either questionable or have been rejected due to lacking of reliable evidence for their pulvini or/and basal actinodromous or acrodromous venations. Besides Oligocene leaves and fruits presented here, foliage and/or wood of *Bauhinia* have been documented from the Miocene–Pliocene of Thailand, India, Nepal, Uganda, and Ecuador.

**Conclusions:**

*Bauhinia* has exhibited a certain diversity with bifoliolate- and bilobate-leafed species in a low-latitude locality–Ningming since at least the Oligocene, implying that the tropical zone of South China may represent one of the centres for early diversification of the genus. The reliable macrofossils of *Bauhinia* and *Cercis* have made their debut in the Eocene–Oligocene floras from mid-low latitudes and appeared to lack in the coeval floras at high latitudes, implying a possible Tethys Seaway origin and spread of legumes. However, detailed scenarios for the historical biogeography of *Bauhinia* and its relatives still need more robust dataset from palaeobotany and molecular phylogeny in future research.

## Background

**Figure 1:**
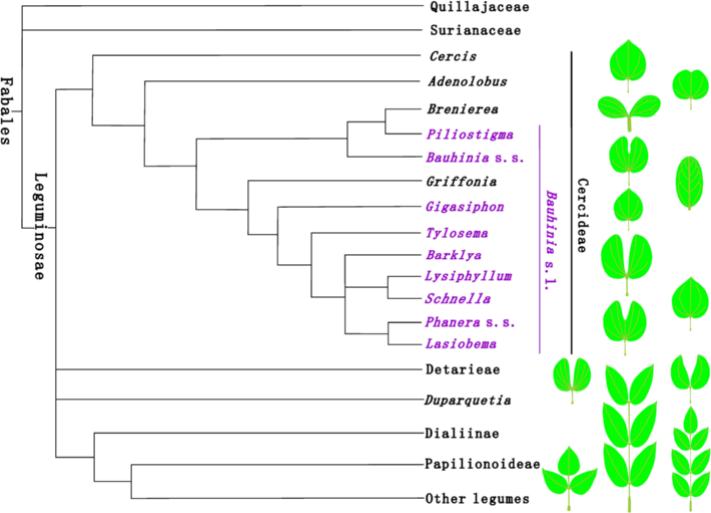
**A simplified phylogenetic tree of the Leguminosae**, **with special reference to the phylogeny of the tribe Cercideae (after **[10]**,**[17]**) and iconic leaf forms enhanced.** The purple taxa belong to *Bauhinia* sensu lato.

*Bauhinia* L. (Leguminosae Juss., Caesalpinioideae DC.) is a pantropical legume genus with ca.150—300 species, the number of which depends on the demarcation of the genus [1-6] (see Additional file 1). The taxonomy of *Bauhinia* is especially complicated, and it has been recognized either as a large genus [1-3,5,7-9], or as 8-9 distinct genera [4,6,10] (Figure 1). Although a taxonomical consensus has not been achieved, recent studies on pollen morphology and molecular systematics of *Bauhinia* have suggested that *Bauhinia* sensu lato is not monophyletic and should be subdivided into *Bauhinia* sensu stricto and other independent genera [6,10-13] (Figure 1). *Bauhinia* is well known for its ornamental shrubs and trees, such as *B. blakeana* Dunn being first chosen as the city flower of Hong Kong, China in 1965. In addition, seeds of *B. petersiana* Bolle are used as a coffee substitute [4]; some species, e.g., *B. championii* (Benth.) Benth., *B. purpurea* L., *B. tomentosa* L., have local pharmacological uses [14-16].

*Bauhinia* was named after two Swiss botanists, the brothers Jean Bauhin (1541–1613) and Gaspard Bauhin (1560–1624), suggesting a brotherly relationship in its commonly bilobate leaves [4]. *Bauhinia*, along with a northern temperate genus *Cercis* L. and several tropical genera, bear bilobate, bifoliolate, or sometimes unifoliolate leaves, which constitute the tribe Cercideae Bronn as sister to the remaining legumes in the molecular phylogenetic trees [17-24]. Bilobate, bifoliolate, or unifoliolate pulvinate leaves with basal actinodromous or acrodromous venations are characteristic for Cercideae [25-27], whereas leaves of other legumes are usually pinnately compound, occasionally trifoliolate or palmate (Figure 1). Hence, well-preserved bilobate, bifoliolate, or unifoliolate pulvinate leaves are easily recognizable in the fossil record and can provide an instrumental evidence for understanding the early evolution and biogeographic history of the Cercideae and the Leguminosae.

The goals of this paper are to (1) investigate and evaluate the fossil record of *Bauhinia*, with special reference to that of *Cercis*, by comparing both extinct and extant angiosperms with the similar lobed leaf forms, (2) describe the foliage and fruit fossils from the Oligocene Ningming Formation of Guangxi, South China, and discuss their biogeographic implications.

## Methods

### Macrofossils

**Figure 2:**
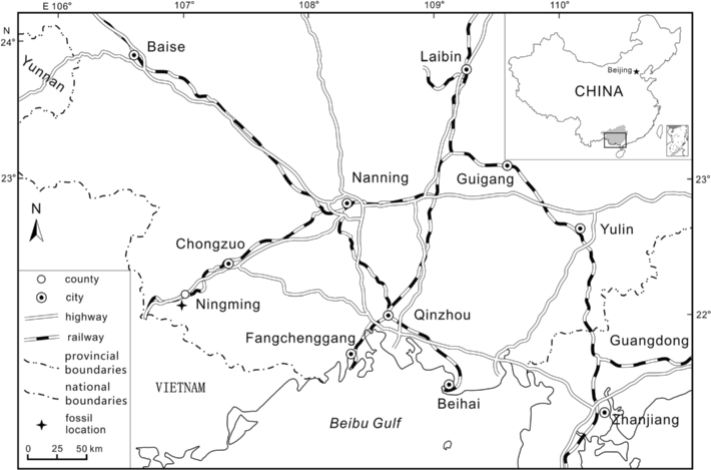
**Fossil locality showing Ningming of Guangxi, South China (after **[36]**).**

The fossil foliage and fruits studied in this paper were collected from the Ningming Formation at 22°07.690’N, 107°02.434’E in the western region of Ningming County, Guangxi Zhuang Autonomous Region, South China (Figure 2). The Ningming Formation is primarily shallow lacustrine deposits consisting of gray to dark gray mudstone, light yellow shaly siltstone, and finely grained sandstone. No volcanic rocks and mammals are hitherto found in the Ningming Formation [28], and an absolute age for this formation is therefore unavailable. The Ningming Basin is among the late Palaeogene basins (e.g., mammal-bearing Na Duong and Bose basins) [29,30] in southern Guangxi and northern Vietnam that experienced a generally similar geological history, so it is inferred that the Ningming Formation is likely to be late Eocene—Oligocene in age. The previous studies on plant macrofossils (e.g., *Palaeocarya ningmingensis* H.M. Li et Y.F. Chen, *P. guangxiensis* H.M. Li et Y.F. Chen, *Bauhinia larsenii* D.X. Zhang et Y.F. Chen, *Cephalotaxus ningmingensis* G.L. Shi et al., *Cupressus guangxiensis* G.L. Shi et al., and *Calocedrus huashanensis* G.L. Shi et al.), sporo-pollen assemblages (e.g., *Quercoidites microhenrici* (Potonié) Potonié), and fishes (e.g., *Ecocarpia ningmingensis* G.J. Chen et al., *Huashancyprinus robustispinus* G.J. Chen et M.M. Chang) from the same locality indicate the Ningming Formation most possibly an Oligocene age [31-39], which is adopted in this paper.

In China, all the land belongs to our country. Our fossil-collecting fieldwork was done in non-National Nature Reserves (NNR) and non-private areas and has been allowed by the local government. We did not violate the Chinese fossil collection and mining laws and management regulations.

The macrofossils are preserved as compressions/impressions only with a little organic material remaining in mudstone. Cuticle preparations were unsuccessful because organic material has been greatly weathered. All the marcofossil specimens used herein are deposited at Natural History Museum of Guangxi (NHMG), Nanning, P.R. China (see Additional file 2).

### Herbaria

The exsiccatae examined in this study are kept at the following herbaria: Chengdu Institute of Biology, Chinese Academy of Sciences, Chengdu (CDBI), Guangxi Institute of Botany, Chinese Academy of Sciences, Guilin (IBK), South China Botanical Garden, Chinese Academy of Sciences, Guangzhou (IBSC), the Herbarium of Northeast China, Shenyang (IFP), Kunming Institute of Botany, Chinese Academy of Sciences, Kunming (KUN), and the Chinese National Herbarium, Beijing (PE) (see Additional file 2).

### Online databases

**Figure 3:**
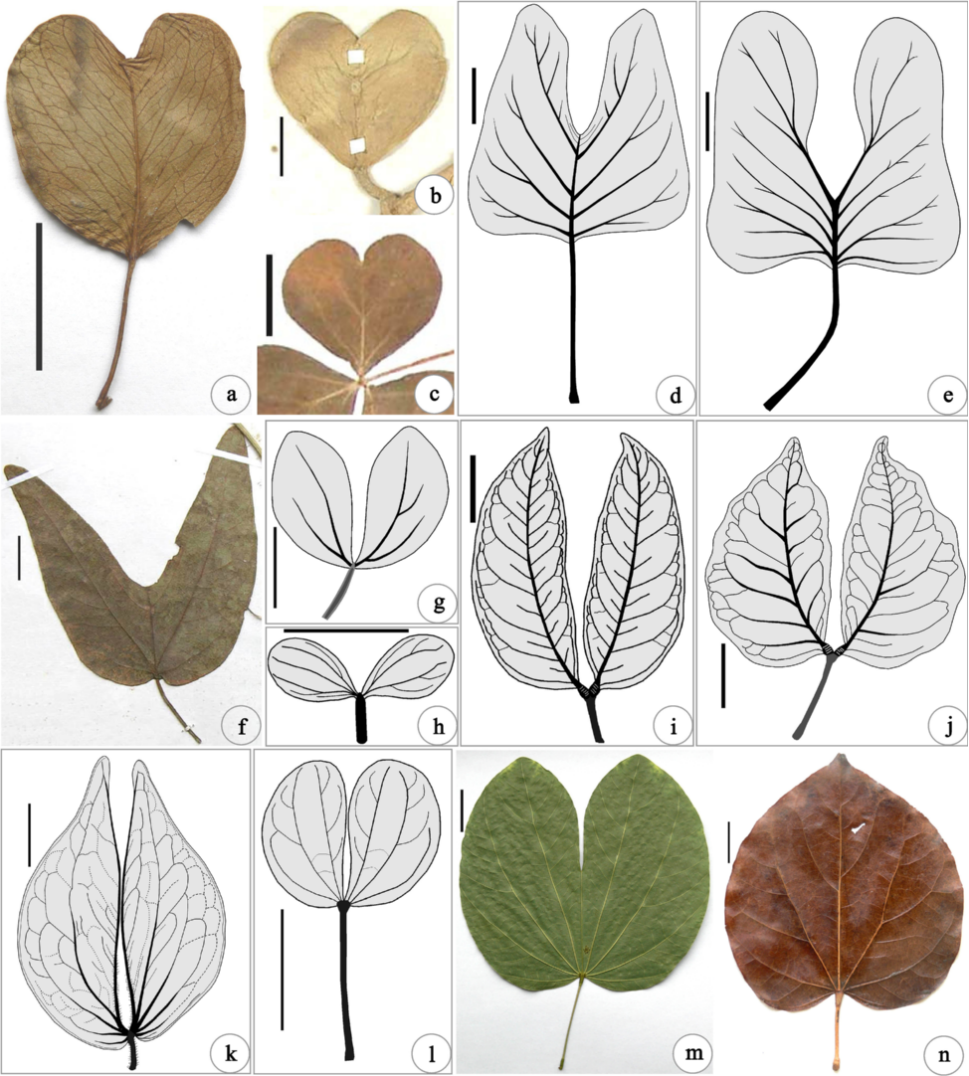
**Similar leaf forms in *Bauhinia *and other angiospermous taxa. (a) ***Ipomoea pes*-*caprae* (L.) R. Br. Specimen: PE12054. **(b)***Hoya kerrii* Craib. Specimen: IBSC199290. **(c)***Oxalis corymbosa* DC. Specimen: PE1688774. **(d)***Liriodendrites bradacii* K.R. Johnson [44]. **(e)***Liriophyllum kansense* Dilcher et P.R. Crane [45]. **(f)***Passiflora cupiformis* Masters. Specimen: KUN0368045. **(g)***Zygophyllum fabago* L. [41]. **(h)***Brenierea insignis* Humbert [46]. **(i)***Hymenaea courbaril* L. [41]. **(j)***Guibourtia coleosperma* (Benth.) J. Léonard [47]. **(k)***Aphanocalyx richardsiae* (J. Léonard) Wieringa [48,49]. **(l)***Bauhinia didyma* L. [41]. **(m)***Bauhinia variegata* L. Cultivated at NHMG, Nanning and photographed by Qi Wang on October 17^th^ 2013, also see the Cover Image of this paper. **(n)***Cercis chinensis* Bunge. Cultivated at the Institute of Botany, Beijing. Scale bars = 2 cm.

**Figure 4:**
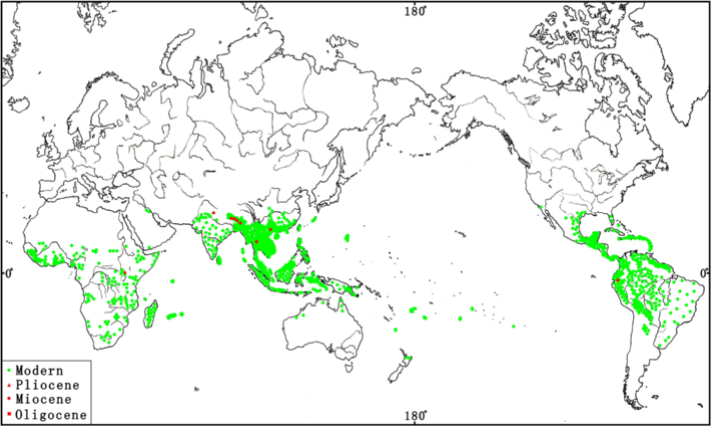
**Distributions of modern and fossil *Bauhinia *L.** (The base map drawn by Mr. Sun Yingbao, Institute of Botany, CAS, Beijing). Green dots indicate modern distributions [42]. Red symbols show fossils from the Oligocene–Miocene of China [33], this paper, Ecuador [50], India [51-54], Nepal [55], Thailand [56], and Uganda [57].

(1) eFloras.org [40]. *Bauhinia* and other living taxa concerned here were consulted (Figure 3; see Additional file 3). (2) Chinese Virtual Herbarium (CVH) [41]. The images of herbarium specimens were browsed. (3) ILDIS (International Legume Database & Information Service) [42]. The geographic distribution of living species in *Bauhinia* is compiled by ILDIS, with a few newly published records (Figure 4; see Additional file 1). (4) Hunt Institute for Botanical Documentation [43]. Standardized abbreviations of plant-family names and periodical titles in this paper were consulted and applied (see References; Additional file 3).

### Terminology

Terms used in the specimen descriptions for leaves and fruits follow Ellis et al. [58]. As for the foliage of the Cercideae, the lower pulvinus has been interpreted as a primary pulvinus at the base of the leaf while the upper pulvinus as a secondary pulvinus homologous with the pulvinus on the leaflet petiolule, consisting of an apical common joint with two distal laminar joints, one for each half of the lamina [25,27,59]. Anatomically, laminar joints are resulted from the tertiary pulvini at the base of each primary vein [27]. Van der Pijl [25] classified the leaves of *Bauhinia* into three basic types: unifoliolate, bilobate, and bifoliolate. Cusset [26] subdivided the leaves of the tribe Bauhinieae Benth. into seven types, but unifoliolate, bilobate, and bifoliolate types epitomize in the foliage of living and fossil species of *Bauhinia*. Hence, unifoliolate, bilobate, and bifoliolate leaves are adopted herein for the description of *Bauhinia* foliage. Time calibrations refer to the latest Geologic Time Scale [60]. The global palaeogeographic maps for the Late Cretaceous, Palaeocene, Eocene, and Oligocene were browsed [61].

### Comparative morphology

Both fossil and extant taxa bearing similar unifolioate, bilobate, and bifoliolate leaves in Leguminosae and other families (Figure 3; see Additional file 3) were compared to evaluate the fossil record and biogeographic history of *Bauhinia*. Based upon an extensive review on the literature and specimens of previously reported *Bauhinia* and other similar foliage from the Cretaceous and Cenozoic, we summarized the reliable fossil record of *Bauhinia* (see Additional file 4).

### Figures

**Figure 5:**
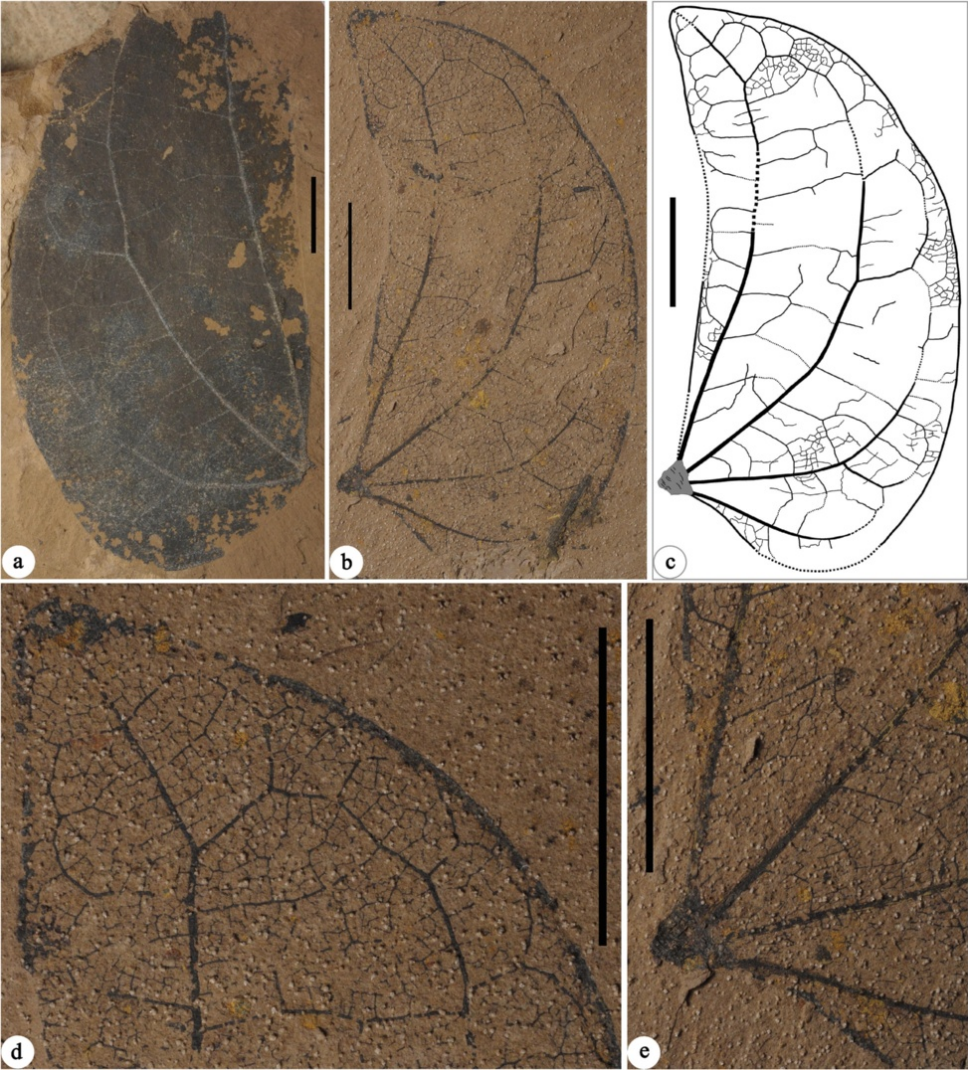
***Bauhinia ningmingensis *sp. nov. from the Oligocene of Ningming, Guangxi, South China.(a)** NHMG 011655. The foliage apex is not preserved. **(b-c)** Holotype: NHMG 011654, and its line-drawing, showing the leaf architectural detail. **(d)** The apex of holotype, showing the higher-order veins. **(e)** The base of holotype, showing the pulvinate tissue. Scale bars = 1 cm.

**Figure 6:**
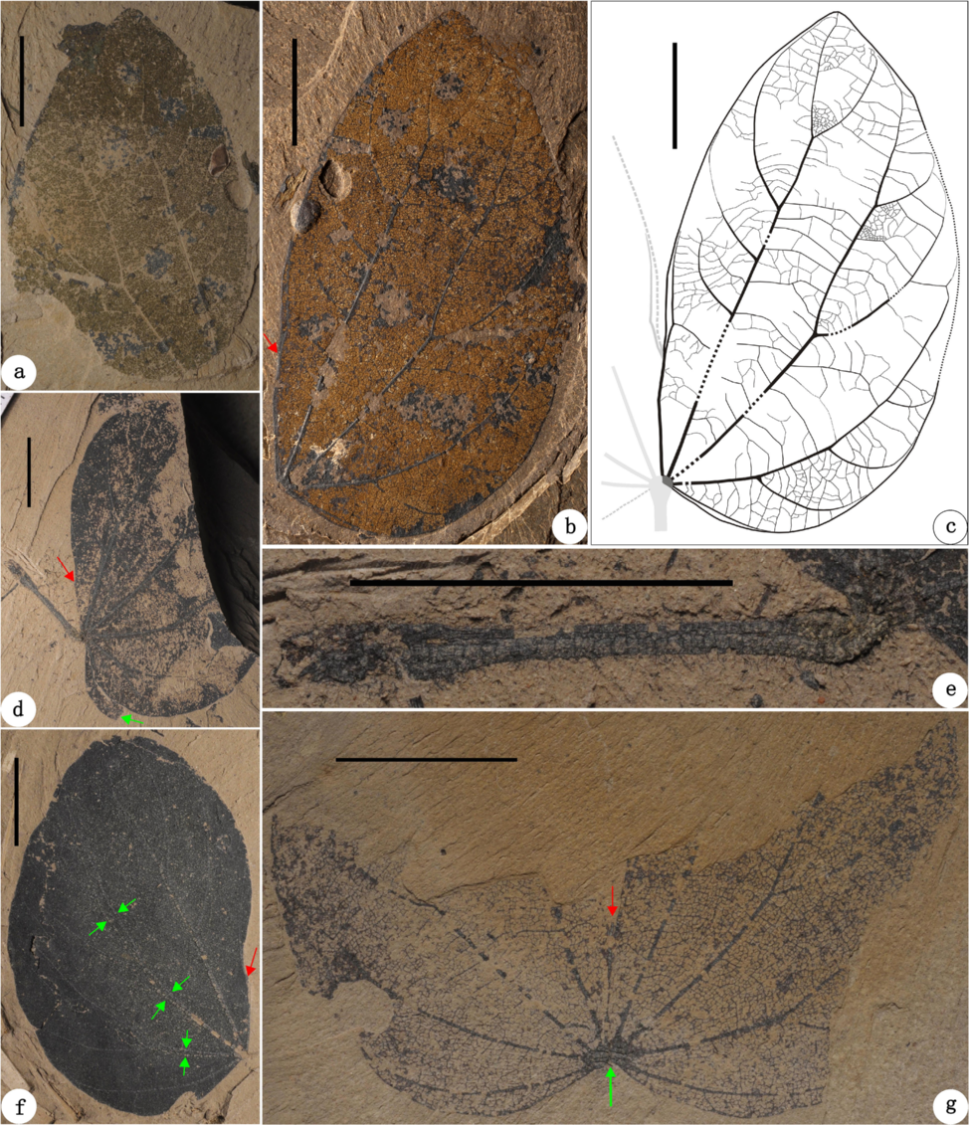
***Bauhinia cheniae *sp. nov. from the Oligocene of Ningming, Guangxi, South China. (a-b)** Holotype: NHMG 011656a, b. Red arrow indicates the position of the sinus. **(c)** Line-drawing of holotype, showing the leaf architectural detail. **(d)** NHMG 011657. Red arrow indicates the position of the sinus. Green arrow shows a folded leaf base. **(e)** Enlargement of the petiole in **d**, showing thickened upper and lower pulvini as well as dense, spreading hairs. **(f)** NHMG 011658. Red arrow indicates the position of the sinus. Green arrows indicate partially overlapped primary veins, implying this leaf is folded. **(g)** NHMG 011659, with the higher-order veins. Red arrow indicates a short spine in the sinus. Green arrow shows a semicircular laminar joint at the leaf base. Scale bars = 1 cm.

**Figure 7:**
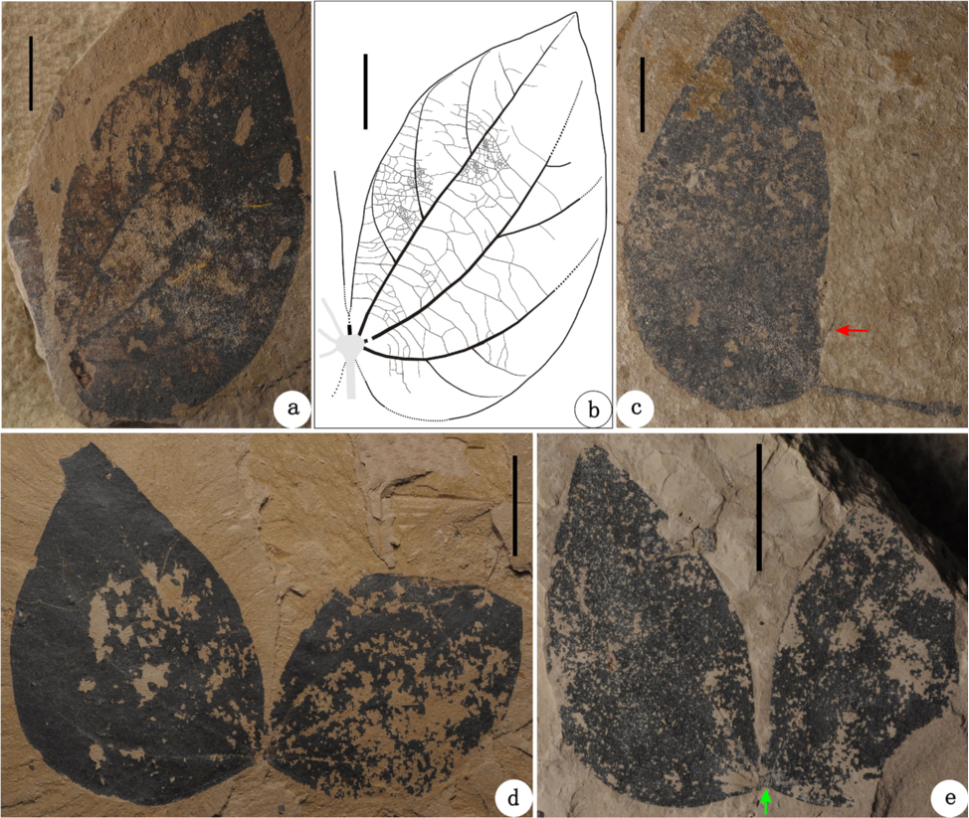
**Additional leaves of *Bauhinia cheniae *sp. nov. from Ningming. (a-b)** NHMG 011670, and its line-drawing, showing the leaf architectural detail. **(c)** NHMG 011671. Red arrow indicates a long spine in the sinus of folded leaf. **(d-e)** NHMG 011672, 011673, showing two deeply bilobate leaves. Green arrow shows a semicircular laminar joint at the leaf base. Scale bars = 1 cm.

**Figure 8:**
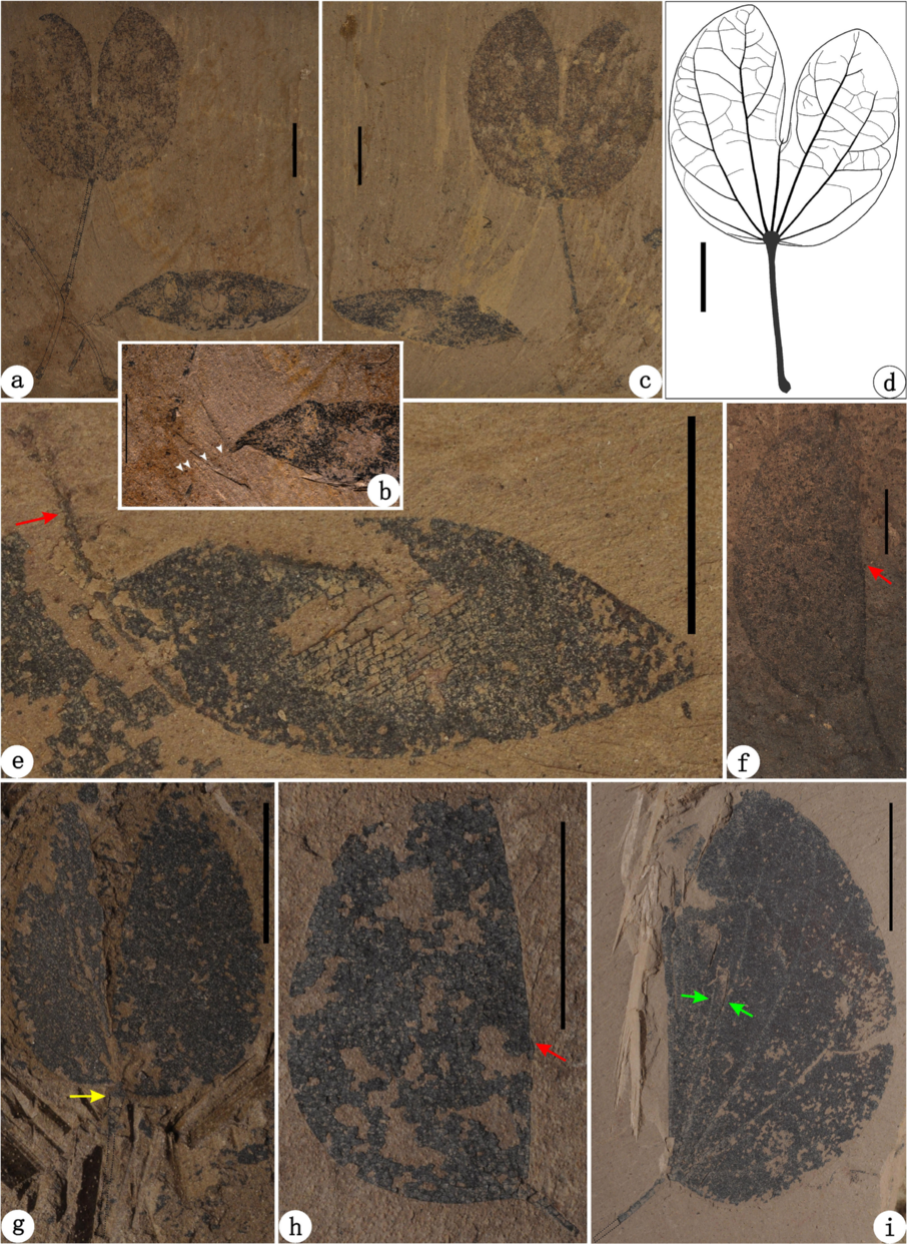
***Bauhinia larsenii *D.X. Zhang et Y.F. Chen emend. from the Oligocene of Ningming**, **Guangxi**, **South China**. **(a-b)** Holotype: NHMG 45003. **(b)** Partial enlargement of holotype. White arrows show an organic connection of the leafy shoot and fruit. **(c-d)** NHMG 45004 and its line-drawing, showing the leaf architectural detail. **(e)** NHMG 45019, showing a detached fruit. Red arrow refers to a thin, long stipe. **(f)** NHMG 45012. Red arrow indicates a short spine in the sinus of folded leaf. **(g**) NHMG 011676. Yellow arrow refers to a thickened upper pulvinus. **(h)** NHMG 011678. Red arrow indicates the position of the sinus of folded leaf. **(i)** NHMG 011677. Green arrows indicate partially overlapped primary veins, implying this leaf is folded. Scale bars = 1 cm.

Photographs of specimens were taken with digital cameras (Panasonic DMC-FZ30 and Nikon D90). A simplified phylogenetic tree (Figure 1) of the Cercideae within Leguminosae was partially adapted from the literature [10,17], with the iconic leaf forms enhanced. A map for the fossil locality (Figure 2) was partially adapted from the literature [36]. Line drawings of leaf specimens for fossil and living taxa as well as of distributional map of *Bauhinia* were drawn (Figures 3, 4, 5, 6, 7 and 8) and arranged using CorelDRAW 10.0 (Corel, Ottawa, Ontario, Canada) and Adobe Photoshop 6.0 (San Jose, California, USA) programmes.

### Abbreviations

The standardized abbreviations for the family name of plants, the author citation of plant names in this paper and journal titles in References follow Brummitt and Powell [62] as well as Botanico-Periodicum-Huntianum (BPH), its supplement (BPH/S), and BPH-2 [63], respectively. The herbarium codes refer to Index Herbariorum [64].

## Results

### Similar leaf architectural comparisons

A leaf lamina partially or fully divided into two lobes is not very common but remarkable among angiosperms. Such leaf forms can be traced back to the Late Cretaceous, for example extinct *Liriodendron*-like angiosperms *Liriodendrites bradacii* K.R. Johnson [44] and *Liriophyllum kansense* Dilcher et P.R. Crane [45] (Figure 3d,e), but some of them have been erroneously identified as *Bauhinia* fossils (see Additional file 4). Overall, both extinct and extant taxa bearing bilobate and bifoliolate foliage occur in Leguminosae, Apocynaceae Juss., Convolvulaceae Juss., Liriodendraceae sensu M.S. Romanov et Dilcher, Oxalidaceae Bercht. et J. Presl, Passifloraceae Juss. ex Roussel, Proteaceae Juss., and Zygophyllaceae R. Br.. In order to determine the reliable fossils of *Bauhinia*, comparisons are made among both extinct and extant taxa with similar lobed leaf forms (Figure 3; see Additional file 3).

### *The taxonomy of Bauhinia* L

The systematics of living *Bauhinia* L. is primarily based upon growth habit, inflorescence, flower, calyx, hypanthium, petal, fertile stamen number, stamen filament, gynophore, stigma, pollen, fruit, seed, and leaf morphology [1-8,12,13], as well as molecular data [9,11]. Species of *Bauhinia* sensu stricto are usually trees or shrubs, rarely semi-scandent plants, whereas those of *Lysiphyllum* (Benth.) De Wit, *Schnella* Raddi, *Tylosema* (Schweinf.) Torre et Hillc., and *Phanera* Lour. (all belonging to *Bauhinia* sensu lato) are lianas, herbaceous vines or rarely shrubs. Different character combinations in reproductive and vegetative organs have been used to classify *Bauhinia* sensu lato into *Bauhinia* sensu stricto and other 7-8 genera, with reference to the molecular phylogenetics of Cercideae [4,6,10] (Figure 1). *Bauhinia* sensu stricto as one of the first branching lineages is the only pantropical genus in the subtribe Bauhiniinae (Benth.) Walp. of Cercideae, so it may boast an earlier origin and evolutionary history than other relatives within the Bauhiniinae.

Morphological characters are the features that ultimately support the distinctiveness of real biological entities, so integral studies mutually illuminating between morphology and molecular systematics will be key in the discrimination of elusive relationships within *Bauhinia* sensu lato. However, considerable convergence, parallelism or evolutionary conservativeness in the organs (especially leaves) of *Bauhinia* sensu stricto and its relatives often place palaeobotanists in a predicament. Without reproductive organs (especially the calyces, fertile stamens, and petals), bilobate leaves of some species in *Bauhinia* sensu stricto, *Phanera*, and *Schnella* would not be distinguished from each other even by neobotanists. Hence, bilobate or bifoliolate leaf fossils in Cercideae were often assigned to *Bauhinia* sensu lato (see Additional file 4).

### The fossil record of Bauhinia and other bilobate leafed taxa

Overall, *Bauhinia* bears mostly bilobate, bifoliolate, or sometimes unifoliolate leaves having characteristic upper and lower pulvini, basal actinodromous or acrodromous primary veins (3-13 in number per leaf), brochidodromous, eucamptodromous or craspedodromous secondary veins, and alternate or opposite percurrent tertiary veins, character combinations of which are noticeably different from the lobed foliage of genera in the tribe Detarieae sensu lato Polhill of Leguminosae, as well as in Apocynaceae, Convolvulaceae, Liriodendraceae, Oxalidaceae, Passifloraceae, Proteaceae Juss., and Zygophyllaceae (see Figure 3; Additional file 3). The simple, unlobed leaves of *Bauhinia* also differ from those of *Cercis* in the tribe Cercideae in the former usually bearing fewer and weaker secondary veins along the distal third of the midvein and a mucro or spine at the leaf tip [59]. Based upon an extensive review on the previously reported *Bauhinia* and other similar foliage from the Cretaceous and Cenozoic, those pre-Oligocene reports regarding *Bauhinia* across the world are either questionable or have been rejected (see Additional file 4). Besides the reliable fossils from Ningming of China, foliage and/or wood of *Bauhinia* have been described from the Miocene-Pliocene of Thailand, India, Nepal, Uganda, and Ecuador.

### Key to the fossil species of Bauhinia from Ningming

1. Lateral primary veins approach to the margin; secondary veins eucamptodromous----------------------------------------------------------------*Bauhinia cheniae* sp. nov.

1. Lateral primary veins do not approach to the margin; secondary veins brochidodromous-------2

2. Bilobate, bifid to ca. 1/2 to 3/5 of laminar length------------------------------------------------*Bauhinia larsenii* D.X. Zhang et Y.F. Chen emend.

2. Bifoliolate, bifid to laminar base-------------------------------------*Bauhinia ningmingensis* sp. nov.

### Systematics

Family Leguminosae Juss.

Subfamily Caesalpinioideae DC.

Tribe Cercideae Bronn

Subtribe Bauhiniinae (Benth.) Walp.

Genus *Bauhinia* L.

Type *Bauhinia divaricata* L.

### Fossil species

Three fossil species of *Bauhinia* are described as follows. All the voucher specimens were collected from the same locality and stratigraphy, and they are deposited at the same museum.

### Type locality

Ningming County, Guangxi Zhuang Autonomous Region, South China (Figure 2).

### Stratigraphic horizon and age

Ningming Formation, Oligocene.

### Repository

Natural History Museum of Guangxi (NHMG), Nanning, P.R. China.

*Bauhinia ningmingensis* Qi Wang, Z. Q. Song, Y. F. Chen, S. Shen et Z. Y. Li, sp. nov. (Figure 5a-e).

### Etymology

The specific epithet is derived from Ningming County, where the fossils were collected.

### Holotype

NHMG 011654 (Figure 5b-e) (designated here).

### Paratypes

NHMG 011655 (Figure 5a) (designated here).

### Diagnosis

Small, bifoliolate leaves. Leaflets pulvinate and laminae strongly asymmetrical, obliquely ovate or slightly falcate. Leaflet apexes obtuse. Bases wide cuneate or slightly concave. Margin entire. Primary veins basal acrodromous (3-4 in number) and curved on the exmedial side, not reaching the leaflet margin. Secondary veins brochidodromous. Tertiary veins percurrent or ramified, straight, convex or sinuous. Quaternary veins forming irregular polygons. Aerolation well developed. Freely ending veinlets mostly branching once. Marginal ultimate veins looped and fimbriate.

### Description

Bifoliolate leaves inferred from the symmetry of individual leaflets. Leaflet laminae strongly asymmetrical, obliquely ovate or slightly falcate (Figure 5a-c), ca. 4.0-5.3 cm long and 2.0-2.6 cm wide, with partially preserved pulvinate tissue, ca. 2.5 mm long, showing some horizontal striations (Figure 5c,e). The petiole not preserved. Texture apparently membranous to chartaceous. Leaflet apexes obtuse (Figure 5b,d). Bases wide cuneate or slightly concave (Figure 5a-c,e). Margin entire. Primary veins basal acrodromous, 3-4 in number. Innermost primary veins straight or curved, extremely approaching to the leaflet margin at the base and reaching the leaflet apex. Primary veins on the exmedial side curved, connecting with secondary veins to form a series of arches and loops, not reaching the leaflet margin (Figure 5a-c). Outermost primary veins shorter and weaker. Secondary veins brochidodromous, diverging at ca. 60°-90° from the primary veins on the exmedial side. Tertiary veins alternate and opposite percurrent or ramified, straight, convex or sinuous (Figure 5a-c). Quaternary veins forming irregular polygons. Aerolation well developed. Freely ending veinlets mostly branching once. Marginal ultimate veins looped and fimbriate (Figure 5b-d).

### Comparisons

This fossil new species *B. ningmingensis* is very similar to living *B. madagascariensis* subsp. *meridionalis* Du Puy et R. Rabev. [46] and *B. didyma* L. Chen [3,5,41] (Figure 3l; see Additional file 3), which are distributed in southeastern Madagascar and southern China, Myanmar, northern Thailand, respectively. However, it noticeably bears much larger leaflets (ca. 4.0-5.3×2.0-2.6 cm) than those of the two living species (ca. 0.7-3 × 04-1.7 cm and 1.2-2.4 × 0.9-1.6 cm). In addition, *B. ningmingensis* sp. nov. is different from the previously reported bifoliolate fossil species *B. ecuadorensis* E.W. Berry [50] and *B. siwalika* U. Lakh. et N. Awasthi [51] from the Miocene of India and Ecuador in the leaf architectural detail (see Additional files 3 and 4).

*Bauhinia cheniae* Qi Wang, Z. Q. Song, Y. F. Chen, S. Shen et Z. Y. Li, sp. nov. (Figures 6a-g, 7a-e).

### Etymology

The specific epithet is dedicated to Prof. Chen Dezhao (Chen Te-chao) (South China Botanical Garden, CAS) for her important contribution to the taxonomy of Cercideae.

### Holotype

NHMG 011656a, b (Figure 6a-c) (designated here; part and counterpart specimens).

### Paratypes

NHMG 011657, 011658, 011659 (Figure 6d-g), 011670 (Figure 7a,b), 011671, 011672, 011673 (Figure 7c-e), 011674, 011675, 011660, 011661, 011662, 011663, 011664, 011665, 011666, 011667, 011668, and 011669 (designated here).

### Diagnosis

Small, broadly ovate or suborbicular, moderately or deeply bilobate leaves. Petioles glabrescent or hairy, bearing thicken upper and lower pulvini. Lobe apexes slightly acuminate, obtuse or rounded. Laminar bases shallowly to deeply cordate. Margin entire. Primary veins basal actinodromous, 7-9 in number. The midvein terminated in a short or long spine within a narrow or flaring sinus. Lateral primary veins straight or curved, branched or unbranched, approaching to the margin. Secondary veins eucamptodromous. Intersecondary veins present. Tertiary veins percurrent or ramified, mostly convex, sinuous or rarely straight. Quaternary veins percurrent, forming irregular polygons. Aerolation well developed. Freely ending veinlets mostly unbranched. Marginal ultimate veins absent.

### Description

Bilobate leaves, wide ovate or suborbicular, ca. 2.0-6.0 cm long and 2.2-6.5 cm wide, often folded along the midvein (Figures 6a-c, 7a-e). The petiole glabrescent or covered with dense, spreading hairs (Figure 6d,e).The petiole stout, ca. 1.6-2.0 cm long, bearing thickened, upper and lower pulvini (Figures 6d,e 7c). The upper pulvinus connecting the laminar base via a tiny, semicircular laminar joint. Bifid to ca. 2/3-4/5 of laminar length or almost to the laminar base, forming a narrow or flaring sinus. Two lobes symmetrical or slightly asymmetrical. Lobe apex slightly acuminate, obtuse or rounded (Figures 6a-d,f and 7a-e). Laminar base symmetrical, shallowly to deeply cordate (Figures 6a-d,f,g, 7a-e). Margin entire. Texture apparently chartaceous. Primary veins basal actinodromous, 7-9 in number, the outmost pair and midvein being weaker than the inner pairs. Midvein terminated in a short or long spine within the sinus (Figures 6g, 7c). Lateral primary veins straight or curved, branched or unbranched, and the innermost pair reaching the lobe apex and outer pairs approaching to the margin. Secondary veins eucamptodromous, diverging at ca. 30°-80° mainly from the innermost and outmost lateral primary veins and arching upward along the margin (Figures 6a-c,f,g, 7a,b). A pair of secondary veins usually emitting from the midvein near the sinus and approaching to the inner margin of lobes (Figure 6b-d, f). Intersecondary veins sometimes present, parallel to subjacent secondary veins. Tertiary veins alternate and opposite percurrent or ramified, mostly convex, sinuous or rarely straight. Quaternary veins alternate and opposite percurrent, forming irregular polygons (Figures 6b,c,g, 7a,b). Aerolation well developed. Freely ending veinlets mostly unbranched. Marginal ultimate veins absent.

### Comparisons

This fossil new species *B. cheniae* is very similar to living *B. variegata* L. (Figure 3m) and *B. purpurea* L. [3,5,41] in having deeply bilobate leaves and eucamptodromous secondary veins, but it differs from the extant species in having the fewer primary veins and a densely hairy petiole (see Additional file 3). In these respects, *B. cheniae* sp. nov. is also different from the previously reported, bilobate leafed fossil species *B. larsenii* D.X. Zhang et Y.F. Chen [33], *B. krishnanunnii* A.K. Mathur et al. [52], *B. ramthiensis* Antal et N. Awasthi [53], *B. nepalensis* N. Awasthi et N. Prasad [55], *Bauhinia* sp. 1 [56], *Bauhinia* sp. 2 [54], and *B. waylandii* R.W. Chaney [57] from the Miocene–Pliocene of India, Nepal, Thailand, and Uganda (see Additional files 3 and 4).

*Bauhinia larsenii* D.X. Zhang et Y. F. Chen emend. Qi Wang, Z. Q. Song, Y. F. Chen, S. Shen et Z. Y. Li (Figure 8a-i).

*Bauhinia larsenii* D.X. Zhang et Y. F. Chen, see Chen and Zhang in *Bot. J. Linn. Soc*. 147: 439, Figures 1, 2, 3, 5 and 6, 2005.

### Holotype

NHMG 45003 (Figure 8a,b herein) (first designated and illustrated by Chen and Zhang [33]: Figure 1]).

### Paratypes

NHMG 45004 (counterpart specimen of holotype; first figured herein, Figure 8c,d), 45012 and 45019 (first designated and illustrated by Chen and Zhang [33]: Figures 2 and 6; Figure 8f,e herein).

### Other specimens examined herein

NHMG 011676, 011678, 011677 (Figure 8g-i), and 011679.

### Emended description

Leaves suborbicular or slightly ovate to wide ovate, ca. 2.1-4.5 cm long and 1.8-4.8 cm wide, usually folded along the midvein (Figure 8a-d, f-i). Bifid to ca.1/2-3/5 of laminar length, forming a narrow sinus. Two lobes symmetrical or slightly asymmetrical. Lobe apex rounded to obtuse (Figure 8a,c,d,f). Laminar base symmetrical, rounded or shallowly cordate (Figure 8a,c,d,g). Margin entire. Texture apparently chartaceous to coriaceous. Primary veins basal actinodromous, 5-9 in number, the outmost pair being weaker than the midvein and inner pairs. Midvein terminated in a short spine within the sinus. Lateral primary veins branched or unbranched, and the innermost pair reaching the lobe apex. Major secondary veins brochidodromous, diverging at ca. 45°—60° from the lateral primary veins mainly on the exmedial side and sporadically on the admedial side (Figure 8d). A pair of minor secondary veins emitting from the midvein near the sinus and approaching to the inner margin of lobes (Figure 8d). Secondary veins fused with each other or the branches of primary veins to form loops near the leaf margin, or arcs between the primary veins. Tertiary veins alternate and opposite percurrent or ramified, convex or sinuous. Quaternary veins alternate percurrent. Marginal ultimate veins looped and fimbriate (Figure 8d). Other higher-order veins invisible. The petiole, ca. 1.0-2.2 cm long, with an upper pulvinus and a lower pulvinus (Figure 8a,c,d,f-i). The upper pulvinus thickened, connecting the laminar base via a tiny, semicircular laminar joint. The lower pulvinus slightly thickened, attached at a curved vegetative shoot at ca. 45°. Not far from the lower pulvinus, a fruit attached on the shoot (Figure 8a-c). The pedicel, ca. 0.2 cm long, with an inferior perianth scar and a fruit, indicating the flower of its parent plant hypogynous (Figure 8a). The fruit elliptic, ca. 2.8-3.5 cm long and 1.0-1.1 cm wide, with a slightly curved, acuminate base and an acute apex (Figure 8a-c,e). The fruit base bearing a thin stipe, ca. 0.4-0.8 cm long (Figure 8e). The suture slightly thickened and non-winged. Carbonaceous remnants with an oblique orientation on the valve surface (Figure 8e) implying the fruit unilocular, possibly coriaceous in texture, and tardily dehiscent. Seeds, about 2-4 in number in a fruit, grossly elliptic or oblong in contour (Figure 8a-c), ca. 0.3-0.6 cm long and 0.2-0.3 cm wide, oriented with their length perpendicular to the fruit length.

### Comparisons

This fossil species *B. larsenii* was first described by Chen and Zhang [27] on the basis of four specimens. Here, we emended this species, especially regarding the leaf architecture and fruit morphology, based upon the type specimens and newly collected materials. It is very similar to living *B. viridescens* Desv. and *B. brachycarpa* Wall. ex Benth. [3,5,41] in bearing shallowly or moderately bilobate leaves, brochidodromous secondary vein, and elliptic fruits (see Additional file 3). However, no adequate characters guarantee the fossils to belong to any living species. In addition, *B. larsenii* is different from *B. cheniae* sp. nov. and other fossil species [33,50-57] in the leaf architectural detail (see Additional files 3 and 4). In particular, *B. larsenii* represents the first recognition of *Bauhinia* fruit and foliage organically connected in the fossil record.

## Discussion

The Leguminosae is the third largest angiosperm family only after Orchidaceae Juss. and Asteraceae Bercht. et J. Presl, varying in habit from herbs to shrubs, vines, lianas, and trees, with an extremely high diversity of ca. 751 living genera and ca. 19,500 species [10,17] across different habitats of the world. Meanwhile, this family has an abundant and diverse fossil record, and its characteristic fruits, flowers, pollen, foliage, and wood have been well recognized from numerous Cenozoic localities around the world [65,66]. However, an outstanding incongruence between the palaeobotanical finds and molecular systematics of legumes is that the earliest fossil record of the tribe Cercideae as sister to all other lineages in the molecular phylogenetic trees of Leguminosae [9-14,20-24] has so far occurred later than that of some derived tribes bearing compound leaves such as Sophoreae Spreng. ex DC. in the subfamily Papilionoideae L. ex DC. [67]. Such an incongruence implies that the extant Cercideae bearing the simple, entire or bilobate to bifoliolate foliage is unlikely to be the most primitive in Leguminosae, but the derived as some authors formerly suggested from an extinct legume ancestor possibly with palmately compound leaves [68] or pinnately compound leaves [27,69]. The fossil record of the Cercideae lacking or being fewer than those derived tribes in the early Palaeogene of middle latitudes is because either the early distribution of the Cercideae might be restricted to low latitudes, or palaeobotanical studies on the coeval legumes from low latitudes are relatively inadequate [70]. Hence, the Cercideae fossils, especially from low latitudes, can provide an historical perspective for their early evolution, adaptive radiation, and biogeographic history.

Leaves of *Cercis* have been reported from Late Cretaceous and Cenozoic sediments, but the overwhelming majority of these reports have been rejected, questioned, revised [59,71], or in need of confirmation by reinvestigation of the original materials and discovery of better preserved materials [69] (see Additional file 4). The oldest reliable fossils of *Cercis* are represented by the foliage and/or fruits (i.e., *C. parvifolia* Lesq., *C. herbmeyeri* H. Jia et Manchester) from the Late Eocene Florissant Formation, Colorado and John Day Formation, Oregon, western USA [71,72]. In contrast, the foliage fossils of *Bauhinia* have been previously reported from the Late Cretaceous and Palaeogene of North America and Eurasia [73-88], but these identifications are erroneous or unreliable [70] (see Additional file 4). Although *Bauhinia* or *Bauhinia*-like bilobate foliage have been recently reported from the middle Eocene of Tanzania [89], the late Eocene of Vietnam [30], the late Eocene-early Miocene of Brazil [90], and possibly the latest Oligocene–mid-late Miocene of Australia [91], the preservation of these pre-Miocene fossils, which are observed from the originally published figures, appears too poor to reliably assign these leaf fossils to either *Bauhinia* or even Cercideae, because neither the pulvinus nor basal actinodromous or acrodromous venations can be confirmed (see Additional file 4). Instead, the oldest reliable evidence of *Bauhinia* and *Bauhinia*-like foliage are provided from the Oligocene Ningming Formation, Guangxi, South China (i.e., *Bauhinia ningmingensis* sp. nov., *B. cheniae* sp. nov., and *B. larsenii* D. X. Zhang et Y. F. Chen [33], this paper) and Coatzingo Formation, Puebla, Mexico (i.e., *Bauhcis moranii* Calvillo-Canadell et Cevallos-Ferriz [92]). By the Miocene–Pliocene, various species of *Bauhinia* have existed in Thailand, India, Nepal, Uganda, and Ecuador (Figure 4) while those of *Cercis* have become widespread in mid-latitudes of the northern hemisphere [69,71].

In addition, some other unifoliolate or bilobate foliage or fruit fossils from the Oligocene—Miocene of Jinggu (Yunnan), Zhangpu (Fujian), and Ningming (Guangxi) in South China have been reliably described [93-95] or preliminarily identified as *Cercis* and *Bauhinia* (Unpublished observation by Qi Wang, Institute of Botany, Beijing, October 18^th^, 2013). Also, the bilobate foliage extremely similar to *Bauhinia* has been discovered from the Eocene - Oligocene coals of West Sumatra, western Indonesia (vide the image of this leaf fossil and information provided by Drs. Yahdi Zaim, Institute of Technology, Bandung, Indonesia, Peter Wilf, Pennsylvania State University, and Gregg F. Gunnell, Duke University Lemur Center, March 17^th^ 2014). Hence, more Cercideae fossil will be studied and reported from low-latitude tropical zone [96] of East and Southeast Asia. Recently, a strictly east-to-west vicariance for the historical biogeography of *Cercis* has been postulated by molecular data [97]. The Cercideae macrofossils occurring in the Eocene to Oligocene of mid-low latitudes and apparently lacking in the coeval sediments at high-latitudes appear to partially support a tropical Tethys Seaway origin and spread [11,22,23] or an “Out-of-Tropical Asia” dispersal [26] of the Cercideae and the Leguminosae as formerly hypothesized by some authors. However, detailed historical biogeography of Cercideae still need more palaeobotanical and molecular dataset.

## Conclusions

*Bauhinia* has exhibited a certain diversity with three species (i.e., *B. ningmingensi*s, *B. cheniae*, and *B. larsenii*) bearing bifoliolate or bilobate leaves in a low-latitude locality—Ningming since at least the Oligocene, implying the tropical zone of South China may represent one of the centres for early diversification of the genus. The reliable macrofossils of *Bauhinia* and *Cercis* have made their debut in the Eocene—Oligocene floras from mid-low latitudes and appeared to lack in the coeval floras at high latitudes. By the Miocene–Pliocene, various species of *Bauhinia* have existed in Thailand, India, Nepal, Uganda, and Ecuador while those of *Cercis* have become widespread in mid-latitudes of the northern hemisphere. Such a biogeographic pattern implies a possible Tethys Seaway origin and spread for legumes. However, detailed scenarios for the historical biogeography of *Bauhinia* and its relatives still need more robust dataset from palaeobotany and molecular phylogeny in future research.

## Competing interests

The authors declare that they have no competing interests.

## Authors’ contributions

ZYL, YFC, QW, and SS conducted data analyses, taxonomic treatments, and evolutionary and biogeographic interpretations. QW and ZQS photographed specimens, illustrated the line-drawings, and arranged the figures. QW wrote the manuscript and formatted the text. All authors read and approved the final manuscript.

## Supplementary Material

Additional file 1 **The distribution of living species in *Bauhinia *** **L.** Click here for file

Additional file 2 Information on voucher specimens used in this study. Click here for file

Additional file 3 **Comparisons between *Bauhinia *** **species and other taxa with similar foliage.** Click here for file

Additional file 4 **Previously described fossils assignable or similar to *Bauhinia *** **L.** Click here for file
