# Supplementary material for: Leaves and fruits of Bauhinia (Leguminosae, Caesalpinioideae, Cercideae) from the Oligocene Ningming Formation of Guangxi, South China and their biogeographic implications
Source: BMC Evol Biol. 2014 Apr 24;14:88. doi: 10.1186/1471-2148-14-88 (PMC4101841; doi:10.1186/1471-2148-14-88)
Supplement: Additional file 1 — The distribution of living species in Bauhinia L. [file 1471-2148-14-88-S1.doc]

**Additional file 1 The distribution of living species in *Bauhinia* L. sensu lato*** [1—14]**.**

| Accepted names | **Distribution** |
| --- | --- |
| *Bauhinia accrescens* Killip et J.F. Macbr. | Peru |
| *B. acreana* Harms | Bolivia, Brazil, Peru |
| *B. aculeata* L. | India, Caribbean-TRP, El Salvador, Panama, Argentina, Bolivia, Brazil, Ecuador, Peru, Venezuela |
| *B. acuminata* L. | Sierra Leone, Zaire, Bangladesh, Brunei, Cambodia, China (Guangdong, Guangxi, Yunnan, Taiwan), India (Arunachal Pradesh, Assam, Bihar, Goa, Gujarat, Karnataka, Kerala, Madhaya Pradesh, Maharashtra, Manipur, Meghalaya, Nagaland, Orissa, Pondicherry, Punjab, Rajasthan, Tamil Nadu, Tripura, Uttar Pradesh, West Bengal), Indonesia-ISO, Java, Kalimantan, Laos, Myanmar, Pakistan, Philippines, Ryukyu Is, Sri Lanka, Thailand, Vietnam, Andaman Is, Mauritius |
| *B. acuruana* Moric. | Brazil |
| *Bauhinia aherniana* Perkins | Malaysia-ISO, Philippines, Sabah |
| *Schnella alata* (Ducke) Wunderlin  = *Bauhinia alata* Ducke ^[10]^ | Brazil |
| *Schnella altiscandens* (Ducke) Wunderlin  *= Bauhinia altiscandens* Ducke ^[10]^ | Brazil |
| *Bauhinia amambayensis* Fortunato | Paraguay |
| *Bauhinia ampla* Span.  = *Gigasiphon amplum* (Span.) de Wit | East Timor, Indonesia-ISO, Irian Jaya, Papua New Guinea |
| *Schnella anamesa* (J.F. Macbr.) Wunderlin  *= Bauhinia anamesa* J.F. Macbr. ^[10]^ | Brazil (Goias and Mato Grosso), Caribbean-TRP, Northern Marianas |
| *Phanera andersonii* (K. Larsen et S.S. Larsen) Bandyop. et al.  *= Bauhinia andersonii* K. Larsen et S.S. Larsen ^[9]^ | Malaysia-ISO, Peninsular Malaysia |
| *Bauhinia andrieuxii* Hemsl. | Mexico |
| *Schnella angulosa* (Vogel) Wunderlin  *= Bauhinia angulosa* Vogel ^[10]^ | Brazil |
| *Bauhinia ankarafantsikae* Du Puy et R. Rabev. | Madagascar |
| *Bauhinia apertilobata* Merr. et F.P. Metcalf | China (Fujian, Guangdong, Guangxi, Jiangxi) |
| *Tylosema argentea* (Chiov.) Brenan  = *Bauhinia argentea* Chiov. | Kenya, Somalia |
| *Bauhinia argentinensis* Burkart | Argentina, Paraguay |
| *B. aromatica* Ducke | Brazil (Ceara) |
| *B. augustii* Harms | Ecuador, Peru |
| *B. aurantiaca* Bojer | Comoro Is, Madagascar, Mauritius |
| *Bauhinia aurea* H. Lev. | China (Guangxi, Guizhou, Sichuan, Yunnan) |
| *Phanera aureifolia* (K. Larsen et S.S. Larsen) Bandyop. et al.  *= Bauhinia aureifolia* K. Larsen et S.S. Larsen ^[9]^ | Thailand |
| *Bauhinia aureopunctata* Ducke | Brazil |
| *B. baina* J.F. Macbr. | Peru |
| *B. bassacensis* Gagnep. | Cambodia, Indonesia-ISO, Java, Laos, Malaysia-ISO, Myanmar, Peninsular Malaysia, Sabah, Thailand, Vietnam |
| *B. bauhinioides* (Mart.) J.F. Macbr. | Caribbean-TRP, Argentina, Brazil, Paraguay, Venezuela |
| *B. beguinotii* Cufod. | Costa Rica, Panama, Colombia |
| *B. bidentata* Jack | Indonesia-ISO, Malaysia-ISO, Peninsular Malaysia, Sumatra, Thailand |
| *Bauhinia binata* Blanco  *= Lysiphyllum binatum* (Blanco) de Wit | Sudan, China, India, Indonesia-ISO, Java, Lesser Sunda Is, Philippines, Thailand, Australia, Papua New Guinea, Mauritius, Northern Marianas |
| *B. blakeana* Dunn | China (Guangdong), India, Indonesia-ISO, Malaysia-ISO, Papua New Guinea, Mauritius, Reunion, Rodrigues, Northern Marianas |
| *B. bohniana* L. Chen | China (Yunnan) |
| *B. bombaciflora* Ducke | Brazil |
| *B. bowkeri* Harv. | South Africa, Zimbabwe, Australia |
| *B. brachycalyx* Ducke | Brazil, Ecuador |
| *B. brachycarpa* Benth. | China (Gansu, Guangxi, Guizhou, Hubei, Sichuan, Xizang, Yunnan), Laos, Myanmar, Thailand |
| *B. bracteata* (Benth.) Baker | Cambodia, Laos, Myanmar, Thailand, Vietnam |
| *B. brevicalyx* Du Puy et R. Rabev. | Madagascar |
| *B. brevipedicellata* Jarvie | Indonesia-ISO, Kalimantan |
| *Bauhinia brevipes* Vogel | Bolivia, Brazil |
| *Phanera burbidgei* (Stapf) Bandyop. et al.  *= Bauhinia burbidgei* Stapf ^[9]^ | Brunei, Malaysia-ISO, Sabah, Sarawak |
| *Bauhinia burrowsii* EJD Schmidt ^[14]^ | Mozambique |
| *B. buscalionii* Mattei | Somalia |
| *B. calliandroides* Rusby | Bolivia |
| *B. caloneura* Malme | Brazil (Mato Grosso) |
| *Bauhinia calycina* Gagnep. | Cambodia, Malaysia-ISO, Peninsular Malaysia |
| *Phanera campanulata* (S.S. Larsen) Bandyop.  *= Bauhinia campanulata* S.S. Larsen ^[9]^ | Brunei |
| *Bauhinia campestris* Malme | Brazil (Mato Grosso, Mato Grosso do Sul) |
| *B. candelabriformis* Cowan | Brazil (Goias) |
| *B. capuronii* Du Puy et R. Rabev. | Madagascar |
| *B. carcinophylla* Merr. | China, Vietnam |
| *B. cardinalis* Gagnep. | Cambodia, Laos, Vietnam |
| *Lysiphyllum carronii* (F. Muell.) Pedley  *= Bauhinia carronii* F. Muell. | India, Australia (Queensland, Western Australia) |
| *Schnella carvalhoi* (Vaz) Wunderlin  *= Bauhinia carvalhoi* Vaz ^[10]^ | Brazil |
| *Bauhinia catingae* Harms | Brazil |
| *B. chalcophylla* L. Chen | China (Yunnan) |
| *B. chalkos* Cowan | Venezuela |
| *B. championii* (Benth.) Benth. | China (Fujian, Guangdong, Guangxi, Guizhou, Hubei, Hunan, Jiangxi, Zhejiang, Hainan, Hong Kong, Taiwan), India (Assam, Sikkim, West Bengal), Vietnam |
| *B. chapulhuacania* Wunderlin | Mexico |
| *B. cheilantha* (Bong.) Steud. | Brazil |
| *B. cinnamomea* DC. | Brazil, French Guiana, Guyana, Peru, Surinam |
| *Bauhinia claviflora* L. Chen | China (Yunnan) |
| *Phanera clemensiorum* (Merr.) Bandyop. et al.  *= Bauhinia clemensiorum* Merr. ^[9]^ | Vietnam |
| *Phanera coccinea* Lour.  *= Bauhinia coccinea* (Lour.) DC. | China (Yunnan), Laos, Vietnam |
| *Bauhinia comosa* Craib | China (Sichuan, Yunnan) |
| *Bauhinia concreta* Craib | Thailand |
| *Schnella confertiflora* (Benth.) Wunderlin  *= Bauhinia confertiflora* Benth. ^[10]^ | Brazil |
| *Bauhinia conwayi* Rusby | Bolivia, Brazil, Peru |
| *B. cookii* Britton et Rose | Costa Rica, Guatemala, Honduras, Mexico |
| *B. corniculata* Benth. | Bolivia, Brazil, Colombia, Peru |
| *B. coronata* Benth. | Bolivia, Brazil, French Guiana |
| *B. corymbosa* Roxb. | China (Hainan, Guangdong), India (Punjab), Pakistan, Vietnam, Mauritius, New Zealand |
| *B. coulteri* J.F. Macbr. | Mexico |
| *B. crudiantha* (de Wit) Cusset | Malaysia-ISO, Sarawak |
| *Lysiphyllum cunninghamii* (Benth.) de Wit  *= Bauhinia cunninghamii* (Benth.) Benth. | Australia (Queensland) |
| *Bauhinia cuprea* Ridl. | Malaysia-ISO, Peninsular Malaysia |
| *Schnella cupreonitens* (Ducke) Wunderlin  *= Bauhinia cupreonitens* Ducke ^[10]^ | Brazil, Peru |
| *Bauhinia cupulata* Benth. | Brazil, Venezuela |
| *B. curtisii* Prain | Cambodia, Laos, Malaysia-ISO, Peninsular Malaysia, Thailand, Vietnam |
| *B. curvula* Benth. | Brazil |
| *B. damiaoshanensis* T. Chen | China (Guangxi) |
| *B. decandra* Du Puy et R. Rabev. | Madagascar |
| *B. decumbens* Henderson | Malaysia-ISO, Peninsular Malaysia |
| *B. delavayi* Franch. | China (Yunnan) |
| *B. dewitii* K. Larsen et S.S. Larsen | Malaysia-ISO, Sarawak |
| *B. didyma* L. Chen | China (Guangdong, Guangxi) |
| *B. dioscoreifolia* L. Chen | China (Hainan) |
| *B. dipetala* Hemsl. | Caribbean-TRP, Belize, Guatemala, Mexico |
| *B. diphylla* Buch.-Ham. | India (Maharashtra, Punjab, Tamil Nadu), Myanmar, Sri Lanka, Mauritius, Reunion, Rodrigues |
| *B. divaricata* L. | Cayman Is, Cuba, Dominican Republic, Haiti, Jamaica, St Kitts-Nevis, Virgin Is, Belize, Costa Rica, El Salvador, Guatemala, Honduras, Mexico, Nicaragua, United States (Texas), Mauritius |
| *Bauhinia divergens* Baker | India (Arunachal Pradesh, Nagaland), Myanmar |
| *Bauhinia dolichocalyx* Merr.  = *Gigasiphon dolichocalyx* (Merr.) de Wit | Philippines |
| *Phanera dubia* (Vogel) Vaz  *= Bauhinia dubia* Vogel ^[7]^ | Brazil |
| *B. dumosa* Benth. | Brazil |
| *B. eilertsii* Pulle | Surinam |
| *B. ellenbeckii* Harms | Ethiopia, Somalia |
| *B. elmeri* Merr. | Indonesia-ISO, Kalimantan, Malaysia-ISO, Sabah, Sarawak |
| *Bauhinia endertii* K. Larsen et S.S. Larsen | Indonesia-ISO, Kalimantan, Malaysia-ISO, Sabah, Sarawak |
| *Schnella erythrantha* (Ducke) Wunderlin  *= Bauhinia erythrantha* Ducke ^[10]^ | Brazil |
| *Bauhinia erythrocalyx* Wunderlin | Guatemala, Mexico |
| *Bauhinia erythropoda* Hayata | China (Yunnan, Guangxi, Hainan) |
| *Tylosema esculentum* (Burch.) A. Schreib.  = *Bauhinia esculenta* Burch. | Botswana, Namibia-ISO, South Africa |
| *Bauhinia esquirolii* Gagnep. | China (Guizhou) |
| *B. estrellensis* Hassl. | Paraguay |
| *Bauhinia excelsa* (Miq.) Prain | Brunei, Indonesia-ISO, Kalimantan, Malaysia-ISO, Sabah, Sarawak |
| *Phanera excurrens* (Stapf) Bandyop. et al.  *= Bauhinia excurrens* Stapf ^[9]^ | Malaysia-ISO, Sabah |
| *Bauhinia exellii* Torre et Hillc. | Angola-ISO |
| *Phanera fabrilis* (de Wit) Bandyop. et al.  *= Bauhinia fabrilis* (de Wit) K. Larsen et S.S. Larsen ^[9]^ | Malaysia-ISO, Sabah |
| *Bauhinia farek* Desv. | Ethiopia |
| *Tylosema fassoglensis* (Schweinf.) Torre et Hillc.  = *Bauhinia* *fassoglensis* Schweinf. | Angola-ISO, Burundi, Ethiopia, Kenya, Malawi, Mozambique, South Africa, Sudan, Swaziland, Tanzania, Uganda, Zaire, Zambia, Zimbabwe |
| *Bauhinia ferruginea* Roxb. | Indonesia-ISO, Malaysia-ISO, Myanmar, Peninsular Malaysia, Sumatra, Thailand, Nicobar Is |
| *B. finlaysoniana* (Benth.) Baker | Indonesia-ISO, Java, Kalimantan, Malaysia-ISO, Moluccas, Peninsular Malaysia, Philippines, Sabah, Sarawak, Sulawesi, Sumatra |
| *B. flagelliflora* Wunderlin | Ecuador |
| *Bauhinia flava* (de Wit) Cusset | Malaysia-ISO, Peninsular Malaysia |
| *Phanera flexuosa* (Moric.) L.P. Queiroz  *= Bauhinia flexuosa* Moric. ^[6]^ | Brazil |
| *Bauhinia foraminifer* Gagnep. | Brunei, Malaysia-ISO, Sarawak |
| *B. forficata* Link | Tonga, Argentina, Bolivia, Brazil, Paraguay, Peru, Uruguay |
| *Bauhinia foveolata* Dalzell | India (Gujarat, Karnataka, Maharashtra) |
| *Phanera franckii* (K. Larsen et S.S. Larsen) Bandyop et al.  *= Bauhinia franckii* K. Larsen et S.S. Larsen ^[9]^ | Malaysia-ISO, Peninsular Malaysia |
| *B. fryxellii* Wunderlin | Mexico |
| *B. fulva* Korth. | Indonesia-ISO, Java, Sumatra |
| *B. fusconervis* D. Dietr. | Brazil |
| *B. galpinii* N.E. Br. | Ghana, Kenya, Malawi, Mozambique, South Africa, Swaziland, Zambia, Zimbabwe, India (Karnataka, Maharashtra, Punjab, Tamil Nadu, West Bengal), Indonesia-ISO, Malaysia-ISO, Philippines, Sri Lanka, Norfolk Is, Caribbean-TRP, Mauritius, Reunion, Rodrigues, Seychelles, Fiji |
| *B. gardneri* Benth. | Brazil |
| *Bauhinia gilesii* F. Muell. et Bailey | Australia (Western Australia) |
| *Lysiphyllum gilvum* (Bailey) Pedley  = *Bauhinia gilva* (Bailey) Govaerts | Australia (New South Wales, Northern Territory, Queensland, South Australia) |
| *Phanera glabra* (Jacq.) Vaz  *= Bauhinia glabra* Jacq. ^[7]^ | India, Sri Lanka, Caribbean-TRP, Belize, Costa Rica, Mexico, Panama, Brazil, Colombia, Ecuador, French Guiana, Peru, Venezuela |
| *Bauhinia glabrifolia* (Benth.) Baker | Bhutan, Laos, Malaysia-ISO, Myanmar, Peninsular Malaysia, Thailand |
| *B. glabristipes* (de Wit) Cusset | Malaysia-ISO, Sarawak |
| *B. glauca* (Benth.) Benth. | Bangladesh, Myanmar, Cambodia, China (Fujian, Guangdong, Guangxi, Guizhou, Hubei, Hunan, Jiangxi, Sichuan, Yunnan, Hong Kong), India (Arunachal Pradesh, Assam, Manipur, Meghalaya, Mizoram, Nagaland), Indonesia-ISO, Java, Laos, Malaysia-ISO, Myanmar, Peninsular Malaysia, Sumatra, Thailand, Vietnam |
| *B. glaziovii* Taub. | Brazil |
| *B. godefroyi* Gagnep. | Cambodia |
| *Gigasiphon gossweileri* (Baker f.) Torre et Hillc.  = *Bauhinia gossweileri* Baker f. | Angola-ISO, Gabon, Zaire |
| *Bauhinia goyazensis* Harms | Brazil |
| *B. gracillima* (de Wit) Cusset | Malaysia-ISO, Sabah |
| *B. grandidieri* Baill. | Madagascar |
| *Bauhinia grandifolia* D. Dietr. | Brazil |
| *Schnella grazielae* (Vaz) Wunderlin  *= Bauhinia grazielae* Vaz ^[10]^ | Brazil |
| *Bauhinia grevei* Drake | Madagascar |
| *Bauhinia guentheri* Harms | Peru |
| *Schnella guianensis* (Aubl.) Wunderlin  *= Bauhinia guianensis* Aubl. ^[10]^ | Dominica, Guadeloupe, Trinidad & Tobago, Belize, Costa Rica, Mexico, Panama, Bolivia, Brazil, Colombia, Ecuador, French Guiana, Guyana, Peru, Surinam, Venezuela |
| *Bauhinia gypsicola* McVaugh | Mexico |
| *B. hagenbeckii* Harms | Brazil, Paraguay |
| *B. hainanensis* Merr. et Chun | China (Hainan) |
| *B. harmsiana* Hosseus | Cambodia, Thailand |
| *Bauhinia haughtii* Wunderlin | Ecuador |
| *Phanera havilandii* (Merr.) Bandyop et al.  *= Bauhinia havilandii* Merr. ^[9]^ | Brunei, Indonesia-ISO, Kalimantan, Malaysia-ISO, Sabah, Sarawak |
| *Bauhinia hekouensis* T.Y. Tu et D.X. Zhang ^[2]^ | China (Yunnan) |
| *B. hendersonii* (de Wit) Cusset | Indonesia-ISO |
| *B. herrerae* (Britton et Rose) Standl. et Steyerm. | Belize, Guatemala, Mexico |
| *B. hildebrandtii* Vatke | Madagascar |
| *B. hirsuta* Weinm. | Cambodia, China (Yunnan), Indonesia-ISO, Java, Laos, Malaysia-ISO, Peninsular Malaysia, Thailand, Vietnam |
| *B. hirsutiflora* Vaz | Brazil |
| *Bauhinia hirsutissima* Wunderlin | Peru |
| *Lysiphyllum hookeri* (F. Muell.) Pedley  *= Bauhinia hookeri* F. Muell. | India (Karnataka, Punjab, Uttar Pradesh, West Bengal) |
| *Gigasiphon humblotianum* (Baill.) Drake  *= Bauhinia humblotiana* Baill. | Madagascar |
| *Tylosema humifusa* (Pic. Serm. et Roti Mich.) Brenan  = *Bauhinia* *humifusa* Pic. Serm. et Roti Mich. | Kenya, Somalia |
| *Bauhinia humilis* Rusby | Bolivia |
| *B. hymenaeifolia* Hemsl. | Panama |
| *B. hypochrysa* T. Chen | China (Guangxi) |
| *B. hypoglauca* T. Chen | China (Yunnan) |
| *B. integerrima* Benth. | Brazil |
| *B. integrifolia* Roxb. | Indonesia-ISO, Malaysia-ISO, Peninsular Malaysia, Philippines, Sabah, Sumatra, Thailand |
| *B. involucellata* Kurz | Myanmar, Thailand |
| *B. involucrans* Gagnep. | Vietnam |
| *B. japonica* Maxim. | China (Guangdong), Japan (Ryukyu Is) |
| *B. jenningsii* P. Wilson | Caribbean-TRP, Belize, Guatemala, Mexico |
| *B. jucunda* Brandegee | Mexico |
| *B. kalantha* Harms | Tanzania |
| *Bauhinia khasiana* Baker | China (Hainan, Yunnan), India (Arunachal Pradesh, Manipur, Meghalaya, Mizoram, Nagaland), Laos, Vietnam |
| *Phanera kingii* (Prain ex King) Bandyop. et al.  *= Bauhinia kingii* Prain ex King ^[9]^ | Malaysia-ISO, Peninsular Malaysia |
| *Bauhinia kleiniana* Burkart | Brazil |
| *Schnella klugii* (Standl.) Wunderlin  = *Bauhinia klugii* Standl. ^[10]^ | Peru |
| *Bauhinia kockiana* Korth. | Brunei, Indonesia-ISO, Kalimantan, Lesser Sunda Is, Malaysia-ISO, Peninsular Malaysia, Sabah, Sarawak, Sumatra |
| *Phanera kostermansii* (K. Larsen et S.S. Larsen) Bandyop. et al.  *= Bauhinia kostermansii* K. Larsen et S.S. Larsen ^[9]^ | Indonesia-ISO, Kalimantan, Malaysia-ISO, Sabah |
| *Schnella kunthiana* (Vogel) Wunderlin  *Bauhinia kunthiana* Vogel ^[10]^ | Brazil, French Guiana, Guyana, Surinam, Venezuela |
| *Bauhinia kurzii* Prain | Myanmar |
| *B. lakhonensis* Gagnep. | Laos, Thailand, Vietnam |
| *B. lambiana* Baker f. | Brunei, Malaysia-ISO, Sarawak |
| *B. lamprophylla* Harms | Brazil |
| *B. leptantha* Malme | Brazil (Mato Grosso do Sul) |
| *Bauhinia leucantha* Thulin | Somalia |
| *Schnella lilacina* (Wunderlin et Eilers) Wunderlin  = *Bauhinia lilacina* Wunderlin et Eilers ^[10]^ | Brazil |
| *Bauhinia lingua* DC. | Indonesia-ISO, Lesser Sunda Is, Philippines, Sulawesi, Papua New Guinea |
| *B. lingyuenensis* T. Chen | China (Guangxi) |
| *B. longicuspis* Benth. | Bolivia, Brazil, Peru, Venezuela |
| *B. longifolia* (Bong.) Steud. | Bolivia, Brazil, Paraguay, Peru |
| *Bauhinia longipedicellata* Ducke | Brazil |
| *Schnella longiseta* (Ducke) Wunderlin  *= Bauhinia longiseta* Ducke ^[10]^ | Brazil (Amazonas) |
| *Bauhinia longistipes* T. Chen | China (Yunnan) |
| *B. lorantha* Gagnep. | Laos |
| *B. lucida* (Miq.) Prain | Indonesia-ISO, Malaysia-ISO, Peninsular Malaysia, Sumatra |
| *B. lyrata* Raizada | Myanmar |
| *B. macrantha* Oliv. ^[14]^ | Angola |
| *Bauhinia macranthera* Hemsl. | Mexico, United States (Florida, Texas) |
| *Gigasiphon macrosiphon* (Harms) Brenan  = *Bauhinia macrosiphon* Harms | Kenya, Tanzania |
| *Schnella macrostachya* Raddi ^[10, 11]^ | Brazil |
| *Bauhinia madagascariensis* Desv. | Madagascar, Mauritius |
| *Piliostigma malabaricum* (Roxb.) Benth.  *= Bauhinia malabarica* Roxb. | Bangladesh, Bhutan, Myanmar, Cambodia, India (Andhra Pradesh, Arunachal Pradesh, Assam, Bihar, Goa, Gujarat, Haryana, Himachal Pradesh, Jammu-Kashmir, Karnataka, Kerala, Madhaya Pradesh, Maharashtra, Manipur, Meghalaya, Mizoram, Nagaland, Orissa, Pondicherry, Punjab, Rajasthan, Sikkim, Tamil Nadu, Tripura, Uttar Pradesh, West Bengal), Indonesia-ISO, Java, Laos, Lesser Sunda Is, Nepal, Pakistan, Philippines, Thailand, Vietnam, Australia, Andaman Is, Nicobar Is |
| *Bauhinia malacotrichoides* Cowan | Brazil |
| *Bauhinia martinensis* J.F. Macbr. | Peru |
| *Schnella maximilianii* (Benth.) Wunderlin  *= Bauhinia maximilianii* Benth. ^[10]^ | Brazil |
| *Bauhinia meeboldii* Craib | Myanmar |
| *B. membranacea* Benth. | Brazil |
| *B. mendoncae* Torre & Hillc. | Angola-ISO, Zambia |
| *B. menispermacea* Gagnep. | Indonesia-ISO, Kalimantan, Malaysia-ISO, Sarawak |
| *Bauhinia merrilliana* Perkins | Malaysia-ISO, Philippines, Sabah, Sarawak |
| *Phanera microstachya* (Raddi) L.P. Queiroz  *= Bauhinia microstachya* (Raddi) J.F. Macbr. ^[6]^ | Belize, Guatemala, Mexico, Panama, Argentina, Bolivia, Brazil, Colombia, Ecuador, Paraguay, Peru, Venezuela |
| *Bauhinia mollis* (Bong.) D. Dietr. | Argentina, Bolivia, Brazil, Paraguay |
| *B. mombassae* Vatke | Kenya |
| *B. monandra* Kurz | Angola-ISO, Burundi, Ghana, Ivory Coast, Liberia, Mali, Nigeria, Sierra Leone, Somalia, Tanzania, Zaire, Zambia, Bangladesh, Myanmar, India (Karnataka, Maharashtra, Tamil Nadu, West Bengal), Indonesia-ISO, Malaysia-ISO, Sri Lanka, Thailand, Vietnam, Papua New Guinea, Caribbean-TRP, Mexico, Panama, Madagascar, Rodrigues, United States, Cook Is, Fiji, Niue, Northern Marianas, Society Is, Tonga, Brazil, Colombia, French Guiana |
| *B. morondavensis* Du Puy et R. Rabev. | Madagascar |
| *B. multinervia* (Kunth) DC. | Caribbean-TRP, Brazil, Surinam, Venezuela |
| *B. nakhonphanomensis* W. Chatan ^[3]^ | Thailand |
| *B. natalensis* Hook. | South Africa |
| *B. nervosa* (Benth.) Baker | Bangladesh, China, Myanmar, Thailand, India (Assam, Meghalaya) |
| *B. ombrophila* Du Puy et R. Rabev. | Madagascar |
| *Bauhinia ornata* Kurz | China (Hainan, Guangdong, Guangxi, Yunnan), India (Arunachal Pradesh, Assam, Meghalaya, Mizoram, Nagaland), Laos, Myanmar, Thailand, Vietnam |
| *Schnella outimouta* (Aubl.) Wunderlin  = *Bauhinia outimouta* Aubl. ^[10]^ | Brazil |
| *Bauhinia ovatifolia* T. Chen | China (Guangxi), India (Arunachal Pradesh) |
| *B. oxysepala* Gagnep. | Vietnam |
| *B. pachyphylla* Merr. | Philippines |
| *B. pansamalana* Donn. Sm. | Guatemala, Mexico |
| *B. pauciflora* Merr. | Philippines |
| *B. paucinervata* T. Chen | China (Guangxi) |
| *B. pauletia* Pers. | Caribbean-TRP, Costa Rica, El Salvador, Guatemala, Honduras, Mexico, Nicaragua, Panama, Colombia, Venezuela |
| *B. penicilliloba* Gagnep. | Cambodia, Laos, Thailand, Vietnam |
| *B. pentandra* (Bong.) Steud. | Brazil |
| *B. pervilleana* Baill. | Madagascar |
| *B. pes-caprae* Cav. | Mexico |
| *B. petersiana* Bolle | Angola-ISO, Botswana, Malawi, Mozambique, Namibia-ISO, South Africa, Tanzania, Zaire, Zambia, Zimbabwe, India (West Bengal) |
| *B. petiolata* (DC.) Hook. | Panama, Colombia, Venezuela |
| *B. phoenicea* Wight et Arn. | India (Karnataka, Kerala, Maharashtra, Tamil Nadu) |
| *B. pichinchensis* Wunderlin | Ecuador |
| *B. picta* (Kunth) DC. | Panama, Colombia, Venezuela |
| *Bauhinia pinheiroi* Wunderlin | Brazil |
| *Schnella platycalyx* (Benth.) Wunderlin  *= Bauhinia platycalyx* Benth. ^[10]^ | Brazil |
| *Bauhinia platypetala* Benth. | Bolivia, Brazil |
| *B. platyphylla* Benth. | Brazil |
| *Bauhinia podopetala* Baker | Madagascar |
| *Schnella poiteauana* (Vogel) Wunderlin  *= Bauhinia poiteauana* Vogel ^[10]^ | Brazil, French Guiana, Surinam |
| *Schnella porphyrotricha* (Harms) Wunderlin  *= Bauhinia porphyrotricha* Harms ^[10]^ | Brazil, Peru |
| *Bauhinia posthumi* (de Wit) Cusset | Indonesia-ISO, Sumatra |
| *B. pottingeri* Prain | Myanmar |
| *B. pottsii* G. Don | Myanmar, Cambodia, Indonesia-ISO, Java, Kalimantan, Malaysia-ISO, Peninsular Malaysia, Sumatra, Thailand |
| *B. praesignis* Ridl. | Malaysia-ISO, Peninsular Malaysia |
| *B. prainiana* Craib | Myanmar, Thailand |
| *Bauhinia pringlei* S. Watson | Mexico |
| *Schnella pterocalyx* (Ducke) Wunderlin  *= Bauhinia pterocalyx* Ducke ^[10]^ | Brazil |
| *Bauhinia pulchella* Benth. | Brazil |
| *B. pulla* Craib | Cambodia, Thailand |
| *B. purpurea* L. | Ethiopia, Malawi, Mozambique, Nigeria, Sierra Leone, South Africa, Uganda, Zaire, Zambia, Bangladesh, Bhutan, Brunei, China (Hainan, Hong Kong, Taiwan), India (Andhra Pradesh, Arunachal Pradesh, Assam, Bihar, Delhi, Goa, Gujarat, Haryana, Himachal Pradesh, Jammu-Kashmir, Karnataka, Kerala, Madhaya Pradesh, Maharashtra, Manipur, Meghalaya, Mizoram, Nagaland, Orissa, Pondicherry, Punjab, Rajasthan, Sikkim, Tamil Nadu, Tripura, Uttar Pradesh, West Bengal), Indonesia-ISO, Iraq, Laos, Malaysia-ISO, Myanmar (Yanan), Nepal, Pakistan, Philippines, Ryukyu Is, Sri Lanka, Thailand, Vietnam, Papua New Guinea, Barbados, Martinique, Puerto Rico, St Lucia, Costa Rica, El Salvador, Guatemala, Mexico, Panama, Andaman Is, Madagascar, Mauritius, Nicobar Is, United States, Fiji, Northern Marianas, Pitcairn Is |
| *B. pyrrhoclada* Drake | China (Hainan), Vietnam |
| *B. pyrrhoneura* Korth. | Indonesia-ISO, Sumatra |
| *B. quinanensis* T. Chen | China (Guizhou) |
| *Bauhinia racemosa* Lam. | Mauritania, Sierra Leone, Tanzania, Uganda, Bangladesh, Cambodia, China (Yunnan), India (Andhra Pradesh, Arunachal Pradesh, Assam, Bihar, Dadra-Nagar-Haveli, Goa, Gujarat, Haryana, Himachal Pradesh, Karnataka, Kerala, Madhaya Pradesh, Maharashtra, Manipur, Meghalaya, Orissa, Pondicherry, Punjab, Rajasthan, Tamil Nadu, Tripura, Uttar Pradesh, West Bengal), India-ISO, Myanmar, Pakistan, Sri Lanka, Thailand, Vietnam, Dominica, Mauritius |
| *Phanera radiata* (Vell.) Vaz  *= Bauhinia radiata* Vell. ^[7]^ | Brazil |
| *Phanera rahmatii* (Merr.) Bandyop et al.  *= Bauhinia rahmatii* Merr. ^[9]^ | Indonesia-ISO, Sumatra |
| *Bauhinia ramosissima* Hemsl. | Mexico |
| *Schnella reflexa* (Schery) Wunderlin  *= Bauhinia reflexa* Schery ^[10]^ | Panama, Columbia |
| *Piliostigma reticulatum* (DC.) Hochst.  *= Bauhinia reticulata* DC. | Burkina Faso, Cameroon, Central African Republic, Chad, Ethiopia, Ghana, Ivory Coast, Mali, Niger, Nigeria, Senegal, Sudan |
| *Bauhinia richardiana* DC. | Zaire, French Guiana |
| *Phanera ridleyi* (Prain ex King) Bandyop et al.  *= Bauhinia ridleyi* Prain ex King ^[9]^ | Malaysia-ISO, Peninsular Malaysia, Thailand |
| *Schnella riedeliana* (Bong.) Wunderlin  *= Bauhinia riedeliana* Bong. ^[10]^ | Brazil (Mato Grosso do Sul, Sao Paulo) |
| *Bauhinia roxburghiana* Voigt | India (Sikkim, Uttar Pradesh, West Bengal), Nepal |
| *B. rubeleruziana* Donn. Sm. | Belize, Guatemala, Mexico |
| *B. rubro-villosa* K. Larsen et S.S. Larsen | China (Guangxi), Laos, Vietnam |
| *B. rufa* (Bong.) Steud. | Brazil |
| *B. rufescens* Lam. | Benin, Cameroon, Chad, Ghana, Guinea, Guinea Bissau, Ivory Coast, Mali, Mauritania, Niger, Nigeria, Senegal, Sierra Leone, Sudan, Togo, India (Pondicherry, Tamil Nadu, West Bengal), India-ISO |
| *Bauhinia rusbyi* Britton | Bolivia |
| *Phanera rutilans* (Benth.) Vaz  *= Bauhinia rutilans* Benth. ^[7]^ | Brazil, Ecuador, Peru, Venezuela |
| *Bauhinia saccocalyx* Pierre | Laos, Thailand |
| *B. saigonensis* Gagnep. | Cambodia, Laos, Thailand, Vietnam |
| *Bauhinia scala-simiae* Sandwith | Guyana, Venezuela |
| *Lasiobema scandens* (L.) de Wit  = *Bauhinia scandens* L. ^[12]^ | Bangladesh, Bhutan, Cambodia, China (Hainan), India (Andhra Pradesh, Arunachal Pradesh, Assam, Bihar, Goa, Gujarat, Karnataka, Kerala, Madhaya Pradesh, Maharashtra, Meghalaya, Mizoram, Orissa, Pondicherry, Sikkim, Tamil Nadu, Tripura,West Bengal), Indonesia-ISO, Java, Laos, Lesser Sunda Is, Myanmar, Nepal, Sri Lanka, Sumatra, Thailand, Vietnam |
| *Gigasiphon schlechteri* (Harms) de Wit  = *Bauhinia schlechteri* Harms | Indonesia-ISO, Irian Jaya, Papua New Guinea |
| *Bauhinia seleriana* Harms | Guatemala, Honduras, Mexico |
| *B. semibifida* Roxb. | Brunei, India (West Bengal), Indonesia-ISO, Kalimantan, Malaysia-ISO, Myanmar, Peninsular Malaysia, Philippines, Sabah, Sarawak, Sri Lanka, Sulawesi, Sumatra |
| *B. seminarioi* Eggers | Ecuador |
| *B. semla* Wunderlin | Bangladesh, Nepal, Pakistan, India (Andhra Pradesh, Bihar, Haryana, Himachal Pradesh, Karnataka, Madhaya Pradesh, Maharashtra, Orissa, Punjab, Rajasthan, Tamil Nadu, Uttar Pradesh, West Bengal) |
| *B. sessilifolia* (DC.) Quinones | Columbia |
| *B. siamensis* K. Larsen et S.S. Larsen ^[4]^ | Thailand |
| *Bauhinia similis* Craib | Laos, Myanmar, Thailand |
| *Schnella siqueirae* (Ducke) Wunderlin  *= Bauhinia siqueiraei* Ducke ^[10]^ | Brazil, Guyana |
| *Phanera smilacina* (Schott) Vaz  *= Bauhinia smilacina* (Schott) Steud. ^[7]^ | Brazil |
| *Bauhinia somalensis* Pic. Serm. et Roti Mich. | Somalia |
| *Phanera splendens* (Kunth) Vaz  = *Bauhinia splendens* Kunth ^[7]^ | Venezuela |
| *Schnella sprucei* (Benth.) Wunderlin  *= Bauhinia sprucei* Benth. ^[10]^ | Brazil |
| *Barklya syringifolia* F. Muell. ^[11]^ | Queensland |
| *Phanera steenisii* (K. Larsen et S.S. Larsen) Bandyop et al.  *= Bauhinia steenisii* K. Larsen et S.S. Larsen ^[9]^ | Malaysia-ISO, Sabah |
| *Bauhinia stenantha* Diels | Ecuador |
| *Bauhinia stenocardia* Standl. | Brazil |
| *Schnella stenopetala* (Ducke) Wunderlin  *= Bauhinia stenopetala* Ducke ^[10]^ | Brazil |
| *Bauhinia stipularis* Korth. | India (Assam, Meghalaya), Indonesia-ISO, Sumatra, Nicobar Is |
| *B. strychnifolia* Craib | Thailand |
| *B. strychnoidea* Prain | Malaysia-ISO, Peninsular Malaysia |
| *B. subclavata* Benth. | Brazil |
| *Bauhinia subrotundifolia* Cav. | Mexico |
| *Schnella surinamensis* (Amshoff) Wunderlin  *= Bauhinia surinamensis* Amshoff ^[10]^ | Brazil, Guyana, Surinam, Venezuela |
| *Bauhinia sylvani* (de Wit) Cusset | Malaysia-ISO, Sabah, Sarawak |
| *B. taitensis* Taub. | Kenya |
| *B. tarapotensis* Benth. | Brazil, Colombia, Ecuador, Peru |
| *B. tessmannii* Harms | Peru |
| *B. thonningii* Schum. | Angola-ISO, Benin, Botswana, Burkina Faso, Cameroon, Chad, Ethiopia, Gabon, Ghana, Guinea, Guinea Bissau, Ivory Coast, Kenya, Malawi, Mali, Mozambique, Namibia-ISO, Niger, Nigeria, Senegal, Sierra Leone, South Africa, Sudan, Tanzania, The Gambia, Togo, Uganda, Zaire, Zambia, Zimbabwe, Yemen |
| *B. tomentosa* L. | Angola-ISO, Cameroon, Ethiopia, Ghana, Kenya, Nigeria, Sierra Leone, Somalia, South Africa, Tanzania, Zaire, Zambia, Zimbabwe, Bangladesh, China (Guangdong, Hong Kong), India (Andhra Pradesh, Bihar, Delhi, Goa, Gujarat, Haryana, Himachal Pradesh, Jammu-Kashmir, Karnataka, Kerala, Madhaya Pradesh, Maharashtra, Orissa, Pondicherry, Punjab, Rajasthan, Tamil Nadu, Uttar Pradesh, West Bengal), Myanmar (Yanan), Indonesia-ISO, Malaysia-ISO, Nepal, Pakistan, Sri Lanka, Thailand, Vietnam, Papua New Guinea, Antigua-Barbuda, Barbados, Cuba, Dominica, Dominican Republic, Guadeloupe, Martinique, Montserrat, Puerto Rico, St Kitts-Nevis, St Lucia**,** Mauritius, Fiji, Northern Marianas, Tonga, Brazil |
| *B. tortuosa* Collett et Hemsl. | Myanmar |
| *Bauhinia touranensis* Gagnep. | China (Guangxi, Guizhou, Yunnan), India (Arunachal Pradesh), Laos, Myanmar, Vietnam |
| *Schnella trichosepala* (L.P. Quieroz) Wunderlin  = *Phanera trichosepala* L.P. Quieroz ^[6, 10]^ | Brazil |
| *Bauhinia tubicalyx* Craib | Thailand |
| *Bauhinia tuichiensis* Cayola & A. Fuentes ^[13]^ | Bolivia |
| *Bauhinia tumupasensis* Rusby | Bolivia |
| *Schnella uleana* (Harms) Wunderlin  *= Bauhinia uleana* Harms ^[10]^ | Brazil, Peru |
| *Bauhinia ungulata* L. | Belize, Costa Rica, El Salvador, Guatemala, Mexico, Nicaragua, Panama, Seychelles, Bolivia, Brazil, Colombia, Paraguay, Venezuela |
| *B. urbaniana* Schinz | Angola-ISO, Botswana, Namibia-ISO, Zambia |
| *B. urocalyx* Harms | Bolivia, Brazil, Peru |
| *B. uruguayensis* Benth. | Argentina (Corrientes, Misiones), Brazil (Parana, Rio Grande do Sul, Santa Catarina, Sao Paulo), Paraguay |
| *B. vahlii* Wight et Arn. | Zaire, Bhutan, India (Andhra Pradesh, Arunachal Pradesh, Assam, Bihar, Delhi, Goa, Gujarat, Haryana, Himachal Pradesh, Jammu-Kashmir, Madhaya Pradesh, Maharashtra, Orissa, Punjab, Rajasthan, Sikkim, Tamil Nadu, Uttar Pradesh, West Bengal), India-ISO, Nepal, Pakistan, Sri Lanka, Mauritius |
| *B. variegata* L. | Ethiopia, Ghana, Kenya, Malawi, Mozambique, Nigeria, Sierra Leone, South Africa, Tanzania, Uganda, Zaire, Zambia, Zimbabwe, Bangladesh, Bhutan, China (Hainan, Hong Kong), India (Andhra Pradesh, Arunachal Pradesh, Assam, Delhi, Goa, Gujarat, Haryana, Himachal Pradesh, Jammu-Kashmir, Karnataka, Kerala, Madhaya Pradesh, Maharashtra, Manipur, Meghalaya, Mizoram, Nagaland, Orissa, Pondicherry, Punjab, Rajasthan, Sikkim, Tamil Nadu, Tripura, Uttar Pradesh, West Bengal), India-ISO, Indonesia-ISO, Iraq, Laos, Malaysia-ISO, Myanmar (Yanan), Nepal, Pakistan, Sri Lanka, Thailand, Vietnam, Norfolk Is, Papua New Guinea, Bahamas, Dominican Republic, Grenada, Haiti, Puerto Rico, St Lucia, El Salvador, Mexico, Panama, Mauritius, Seychelles, United States (California, Florida, Texas), Fiji, New Zealand, Society Is, Tonga, Brazil, Colombia |
| *B. venustula* T. Chen | China (Guangxi) |
| *B. vespertilio* S. Moore | Brazil |
| *B. wallichii* J.F. Macbr. | Bangladesh, India (Arunachal Pradesh, Assam, Meghalaya, Nagaland, West Bengal), Myanmar, Vietnam |
| *B. weberbaueri* Harms | Ecuador, Peru |
| *B. williamsii* F. Muell. | Papua New Guinea |
| *Bauhinia winitii* Craib  = *Lysiphyllum winitii* (Craib) de Wit | Thailand |
| *Bauhinia wrayi* Prain | Brunei, Indonesia-ISO, Kalimantan, Malaysia-ISO, Peninsular Malaysia, Sabah, Sarawak, Sumatra |
| *Phanera wuzhengyii* (S.S. Larsen) Bandyop et al.  *= Bauhinia wuzhengyii* S.S. Larsen ^[5, 9]^ | Yunnan (China) |
| *Bauhinia xerophyta* Du Puy et R. Rabev. | Madagascar |
| *Phanera yunnanensis* (Franch.) Wunderlin  *= Bauhinia yunnanensis* Franch. ^[8]^ | Brunei, China (Guizhou, Sichuan, Yunnan), Indonesia-ISO, Kalimantan, Myanmar, Thailand |

***** The taxonomy of *Bauhinia* is especially complicated, and it has been recognized either as a large genus or as 7—9 distinct genera. Hence, a taxonomical consensus has not been achieved.

**References**

1. ***International Legume Database & Information Service (ILDIS).*** Published on the Internet [http://www.ildis.org/] (accessed 15 April 2014). Cardiff: Cardiff School of Computer Science & Informatics.

2. Tu TY, Zhang DX: ***Bauhinia hekouensis* (Leguminosae, Caesalpinioideae), a new species from Yunnan, China.** *Novon* 2013, 22:332–335.

3. Chatan W: **A new species of *Bauhinia* L. (Caesalpinioideae, Leguminosae) from Nakhon Phanom Province, Thailand.** *PhytoKeys* 2013, 26:1–5.

4. Larsen K, Larsen SS: ***Bauhinia siamensis* (Leguminosae-Caesalpinioideae), an extraordinary new species from Thailand.** *Nat Hist Bull Siam Soc* 2002, 50:99–104.

5. Larsen SS: ***Bauhinia wuzhengyii* (Leguminosae, Caesalpinioideae), a new Chinese species.** *Novon* 1999, 9:526–529.

6. Queiroz LP: **New species and new combinations in *Phanera* Lour. (Caesalpinioideae: Cercideae) from the Caatinga biome.** *Neodiversity* 2006, 1:6–10.

7. Vaz AMSF: **New combinations in *Phanera* (Leguminosae; Cercideae) from Brazil.** *Rodriguésia* 2010, 61 (Suppl.):S33–S40.

8. Wunderlin BP: **New combination in *Phanera* (Fabaceae).** *Phytoneuron* 2011, 19:1–2.

9. Bandyopadhyay S, Ghoshal PP, Pathak MK: **Fifty new combinations in *Phanera* Lour. (Leguminosae: Caesalpinioideae) from Paleotropical region.** *Bangladesh J Pl Taxon* 2012, 19:55–61.

10. Wunderlin RP: **New combinations in *Schnella* (Fabaceae: Caesalpiniodeae: Cercideae).** *Phytoneuron* 2010, 49:1–5.

11. Wunderlin RP: **Reorganization of the Cercideae (Fabaceae: Caesalpinioideae).** *Phytoneuron* 2010, 48:1–5.

12. De Wit HCD: **A revision of Malaysian Bauhinieae.** *Reinwardtia* 1956, 3:381–539.

13. Cayola Pérez L, Fuentes AF: *Bauhinia tuichiensis* **(Fabaceae, Cercideae), una Especie**

**Nueva del Bosque Seco de la Región Madidi, Bolivia.** *Novon* 2012, 22:148–151.

14. Schmidt EJD: **A new species of *Bauhinia* from southern Mozambique and the reinstatement of *Bauhinia macrantha*.** *Bothalia* 2012, 42:44–47.
