# Supplementary material for: Leaves and fruits of Bauhinia (Leguminosae, Caesalpinioideae, Cercideae) from the Oligocene Ningming Formation of Guangxi, South China and their biogeographic implications
Source: BMC Evol Biol. 2014 Apr 24;14:88. doi: 10.1186/1471-2148-14-88 (PMC4101841; doi:10.1186/1471-2148-14-88)
Supplement: Additional file 2 — Information on voucher specimens used in this study. [file 1471-2148-14-88-S2.doc]

**Additional file 2 Information on voucher specimens used in this study.**

| **Taxon** | **Number** | **Preservation** | **Locality** | **Age** | **Repository** |
| --- | --- | --- | --- | --- | --- |
| *Bauhinia acuminata* L. | 1650948, 1650949 | Herbarium | Ledong, Guangdong, China | Recent | PE |
|  | 359209, 359574 | Herbarium | Guangzhou, Guangdong, China | Recent | IBSC |
|  | 0156551, 0156552 | Herbarium | Mengzi, Yunnan, China | Recent | KUN |
| *Bauhinia brachycarpa* Wall. ex Benth. | 0156592 | Herbarium | Yongren, Yunnan, China | Recent | KUN |
|  | 126787, 126791 | Herbarium | Luding, Sichuan, China | Recent | CDBI |
|  | 1126803 | Herbarium | Qiaojia, Yunnan, China | Recent | PE |
|  | 1411847, 1411593 | Herbarium | Muli, Sichuan, China | Recent | PE |
| *Bauhinia championii* (Benth.) Benth.  [= *Lasiobema championii* (Benth.) De Wit] | 86677 | Herbarium | Shanglin, Guangxi, China | Recent | IBK |
|  | 72122 | Herbarium | Hexian, Guangxi, China | Recent | IBK |
|  | 1744135, 1744210, 1749247 | Herbarium | Hengyang, Hunan, China | Recent | PE |
| *Bauhinia cheniae* Qi Wang et al., sp. nov. | 011656a, b, 011657, 011658, 011659, 011660, 011661, 011662, 011663, 011664, 011665, 011666, 011667, 011668, 011669, 011670, 011671, 011672, 011673, 011674, 011675 | Impression | Ningming, Guangxi, China | Oligocene | NHMG |
| *Bauhinia didyma* L. | 51370 | Herbarium | Yangjiang, Guangdong, China | Recent | IBK |
|  | 140107 | Herbarium | Yangchun, Guangdong, China | Recent | IBK |
|  | 372770 | Herbarium | Yangchun, Guangdong, China | Recent | PE |
|  | 233082 | Herbarium | Yangjiang, Guangdong, China | Recent | IBSC |
| *Bauhinia larsenii* D.X. Zhang et Y.F. Chen | 45003, 45004, 45012, 45019, 011676, 011678, 011677, 011679 | Impression | Ningming, Guangxi, China | Oligocene | NHMG |
| *Bauhinia ningmingensis* Qi Wang et al., sp. nov. | 011654, 011655 | Impression | Ningming, Guangxi, China | Oligocene | NHMG |
| *Bauhinia purpurea* L. | 4980, 04980 | Herbarium | Guangzhou, Guangdong, China | Recent | IBK |
|  | 615960, 01859281 | Herbarium | Jinghong, Yunnan, China | Recent | PE |
|  | 753441 | Herbarium | Nanning, Guangxi, China | Recent | PE |
|  | 1230478, 1230480 | Herbarium | Lingui, Guangxi, China | Recent | PE |
| *Bauhinia variegata* L.  [= *Phanera variegata* (L.) Benth.] | 1697 | Herbarium | Liuzhou, Guangxi, China | Recent | IBK |
|  | 133102 | Herbarium | Wuzhou, Guangxi, China | Recent | IBK |
|  | 0890849, 0988583, 0988584 | Herbarium | Xishuangbanna, Yunnan, China | Recent | PE |
|  | 0157541 | Herbarium | Mojiang, Yunnan, China | Recent | KUN |
|  | 0157545 | Herbarium | Jinghong, Yunnan, China | Recent | KUN |
| *Cercis canadensis* L. | 1477715 | Herbarium | Monroe, Michgan, USA | Recent | PE |
|  | 0158969, 0665814 | Herbarium | Blount, Tennessee, USA | Recent | KUN |
| *Cercis chinensis* Bunge | 1694813 | Herbarium | Beijing, China (cultivated) | Recent | PE |
|  | 1181259, 1181336 | Herbarium | Shennongjia, Hubei, China | Recent | PE |
|  | 00673275, 00673586, 00673742 | Herbarium | Hangzhou, Zhejiang, China (cultivated) | Recent | IBSC |
| *Cercis miochinensis* H.H. Hu et R.W. Chaney | 50610, 50648, 51995, 52907, 990002, 20110509, 20110604 | Impression | Linqu, Shandong, China | Miocene | PE |
| *Cynometra elmeri* Merr. | 55655 | Herbarium | Borneo | Recent | IBSC |
| *Hardwickia binata* Roxb. | 28470, 28471 | Herbarium | India | Recent | IBSC |
| *Hoya kerrii* Craib  [= *Hoya obovata* Decaisne var. *kerrii* (Craib) Costantin.] | 199290 | Herbarium | Guangzhou, Guangdong, China (cultivated) | Recent | IBSC |
|  | 139391 | Herbarium | Chiang Mai, Thailand | Recent | IBSC |
| *Hymenaea verrucosa* Gaertn. | 159337, 476623, 463924 | Herbarium | Guangzhou, Guangdong, China (cultivated) | Recent | IBSC |
| *Ipomoea* *pes-caprae* (L.) R. Br. | 12054 | Herbarium | Xiamen, Fujian, China | Recent | PE |
|  | 107732 | Herbarium | Qingluo, Guangdong, China | Recent | IBK |
|  | 107872 | Herbarium | Huiyang, Guangdong, China | Recent | IBK |
|  | 107578 | Herbarium | Lufeng, Guangdong, China | Recent | IBK |
|  | 8048, 8978, 143294 | Herbarium | Hong Kong, China | Recent | IBSC |
| *Liriodendron tulipifera* L. | 1381329 | Herbarium | Miami, USA | Recent | PE |
|  | 1291241 | Herbarium | Georgia, USA | Recent | PE |
|  | 1118516 | Herbarium | Kyoto, Japan (cultivated) | Recent | PE |
|  | 1455184 | Herbarium | Beijing, China (cultivated) | Recent | PE |
| *Oxalis corymbosa* DC. | 1688774 | Herbarium | Shikokuchuo, Ehime Prefecture, Japan | Recent | PE |
|  | 705186 | Herbarium | Leqing, Zhejiang, China | Recent | PE |
|  | 01767332 | Herbarium | Boyang, Jiangxi, China | Recent | PE |
| *Passiflora cupiformis* Masters | 844, 93122 | Herbarium | Donglan, Guangxi, China | Recent | IBK |
|  | 457040, 457041, 457042 | Herbarium | Xingyi, Guizhou, China | Recent | IBSC |
|  | 457032, 457036, 457037 | Herbarium | Anlong, Guizhou, China | Recent | IBSC |
|  | 0368045, 0368049 | Herbarium | Lijiang, Yunnan, China | Recent | KUN |
| *Zygophyllum* *fabago* L. | 054668, 054669, 054670 | Herbarium | Jinta, Gansu, China | Recent | IFP |
|  | 054592, 054593 | Herbarium | Jiuquan, Gansu, China | Recent | IFP |
|  | 038037, 038038, 038039 | Herbarium | Manasi, Xinjiang, China | Recent | CDBI |

***Notes:*** CDBI: Chengdu Institute of Biology, Chinese Academy of Sciences, Chengdu; IBK: Guangxi Institute of Botany, Chinese Academy of Sciences, Guilin; IBSC: South China Botanical Garden, Chinese Academy of Sciences, Guangzhou; IFP: the Herbarium of Northeast China, Shenyang; KUN: Kunming Institute of Botany, Chinese Academy of Sciences, Kunming; NHMG: Natural History Museum of Guangxi, Nanning; PE: the Chinese National Herbarium, Beijing (see Index Herbariorum [http://sweetgum.nybg.org/ih/]).
