# Supplementary material for: Leaves and fruits of Bauhinia (Leguminosae, Caesalpinioideae, Cercideae) from the Oligocene Ningming Formation of Guangxi, South China and their biogeographic implications
Source: BMC Evol Biol. 2014 Apr 24;14:88. doi: 10.1186/1471-2148-14-88 (PMC4101841; doi:10.1186/1471-2148-14-88)
Supplement: Additional file 3 — Comparisons between Bauhinia species and other taxa with similar foliage. [file 1471-2148-14-88-S3.doc]

**Additional file 3** **Comparisons between *Bauhinia* species and other taxa with similar foliage.**

| **Species (Family)** | **Leaf / Leaflet** | | | | | | **Venation** | |
| --- | --- | --- | --- | --- | --- | --- | --- | --- |
|  | **Shape** | **Size** | **Apex** | **Base** | **Petiole** | **Texture** | **Primary vein** | **Secondary vein** |
| *Adenolobus garipensis* (E. Mey.) Torre et Hillc.^[1—3, 15]^ (LEGU) | Bilobate, suborbicular or wide orbicular, each lobe semi-orbicular | 0.8—2.0×1.2—3.0 cm | Slightly emarginate or very shallowly bilobed | Cordate | Pulvinate, glabrous, (0.5-) 0.8—1.8 cm long | Subcoriaceous | Basal actinodromous, 3—5 in number | Brochidodromous, moderately developed on the midvein |
| *Aphanocalyx cynometroides* Oliv. ^[4, 14]^ (LEGU) | Bifoliolate, leaflet somewhat elliptic | 3—25×1—10 cm (leaflet) | Acute or acuminate | Cuneate | Pulvinate, densely velvety or glabrous, 0.1—0.5 cm long | Coriaceous | Basal acrodromous, 4 in number, 2 per leaflet | Brochidodromous, numerous |
| *Aphanocalyx djumaensis* (De Wild.) J. Léonard ^[4, 14]^ (LEGU) | Bifoliolate, leaflet semi-ovate to semi-obovate | 2.1—12.7×0.9—4.7 cm (leaflet) | Acute or acuminate | Rounded or cuneate | Pulvinate, densely velvety or glabrous, 0.2—1 cm long | Thickly coriaceous | Basal acrodromous, 4 in number, including 1 inner-marginal per leaflet | Brochidodromous, 4 in number per leaflet |
| *Aphanocalyx richardsiae* (J. Léonard) Wieringa  [= *Monopetalanthus richardsiae* J. Léonard] ^[3^, ^4, 9, 14]^ (LEGU) | Bifoliolate, ovate or ovate-elliptic, leaflet semi-ovate-elliptic or semi-ovate-elliptic | 0.9—13×0.5—4.5 cm (leaflet) | Acute or (long) acuminate | Rounded or cuneate, rarely auriculate | Pulvinate, densely velvety or glabrous, 0.1—0.7 cm long | Thinly to thickly coriaceous | Basal acrodromous, 4 in number, including 1 inner-marginal per leaflet | Eucamptodromous to brochidodromous, 4—6 in number per leaflet |
| *Aphanocalyx singidaensis* Herend. et B.F. Jacobs^[4]^ (fossil LEGU) | Bifoliolate, leaflet obliquely ovate, somewhat falcate | At least 6 ×1.9—2.8 cm (leaflet) | Apparently acute | Acute to obtuse | Pulvinate, 2 cm long | Unknown | Basal acrodromous, 2 in number, 1 per leaflet | Eucamptodromous, ca. 10 in number, with 1—2 intersecondaries |
| *Bauhcis moranii* Calvillo-Canadell et Cevallos-Ferriz^[26]^ (fossil LEGU) | Bilobate, wide ovate to elliptic-oblate | 4.3—4.5×5.8—6.4 cm | Bifid to 1/8—1/7 of laminar length | Truncate or cordate | Pulvinate, at least 0.5—1.2 cm long | Unknown | Basal actinodromous, 7 in number | Brochidodromous, with 3—5 intersecondary veins on the midvein |
| *Bauhinia acuminata* L. ^[5, 6, 8, 17, 36]^ (LEGU) | Bilobate, ovate, ovate-orbicular to suborbicular | 7—12×8—12.5 cm | Bifid to 1/3—2/5 of laminar length | Cordate | Pulvinate, pubescent, 2.5—4 cm long | Subcoriaceous | Basal actinodromous, 7—11 in number, outmost veins weak, midvein apex spinose | Brochidodromous, usually rare |
| *Bauhinia binnata* Blanco  [= *Lysiphyllum binatum* (Blanco) de Wit] ^[2, 3, 8]^ (LEGU) | Bifoliolate, leaflet wide ovate | 2.5—4.5×2.5—3.5 cm (leaflet) | Rounded at leaflet apex | Wide cuneate | Pulvinate, slender, 1.0—2.5 cm long | Chartaceous | Basal actinodromous, 8—10 in number | Brochidodromous |
| *Bauhinia brachycarpa* Wall. ex Benth. ^[2, 5, 6, 17, 36]^ (LEGU) | Bilobate, suborbicular | 0.5—12×1—10 cm | Bifid to ca. 1/3—1/2 of laminar length; lobe apex rounded | Truncate or shallowly cordate | Pulvinate, 0.6—6 cm long | Chartaceous or membranous | Basal actinodromous, 5—13 in number, outmost veins weak, midvein apex spinose | Brochidodromous, none on the midvein |
| *Bauhinia championii* (Benth.) Benth.  [= *Lasiobema championii* (Benth.) de Wit] ^[2, 3, 5, 6—8, 17, 36]^ (LEGU) | Bilobate or entire, ovate or cordate | 3—10×2.5—9 cm | Acute, obtuse or emarginate, or variously bilobed; lobe apex acute, acuminate or obtuse | Truncate, cordate, rounded or concave | Pulvinate, slender, 1.0—2.5 cm long | Chartaceous to coriaceous | Basal actinodromous, 5—9 in number | Brochidodromous, 3—4 in number, none on the midvein |
| *Bauhinia cheniae* Qi Wang et al. ^[36, this paper]^ (fossil LEGU) | Bilobate, wide ovate or suborbicular | 2.0—6.0×2.2—6.5 cm | Bifid to ca. 2/3—4/5 or almost to the base; shortly acuminate, obtuse or rounded at lobe apex | Moderately or deeply cordate | Pulvinate, with spreading hairs or no trichomes, 1.6—2.0 cm long | Apparently chartaceous | Basal actinodromous, 7—9 in number | Eucamptodromous, a pair on the midvein near the sinus |
| *Bauhinia didyma* L. Chen ^[2, 5, 6, 17, 36]^ (LEGU) | Bifoliolate, suborbicular, leaflet obliquely obovate | 1.2—2.4×0.9—1.6 cm (leaflet) | Obtuse or rounded at leaflet apex | Truncate | Pulvinate, glabrous, 1—2.4 cm long | Membranous | Basal actinodromous, 6 in number, 3 per leaflet | Brochidodromous |
| *Bauhinia ecuadorensis* E.W. Berry^[27]^ (fossil LEGU) | Bifoliolate, suborbicular, leaflet obliquely ovate-elliptic | 5.25×2.5 cm (leaflet) | Rounded at leaflet apex | Wide cuneate | Pulvinate, stout, ca. 1.5 cm long | Apparently thin and stiff | Basal actinodromous, 8 in number, 4 per leaflet | Brochidodromous, numerous |
| *Bauhinia fassoglensis* Schweinf.  [= *Tylosema fassoglensis* (Schweinf.) Torre et Hillc.] ^[2, 3^, ^9]^ (LEGU) | Bilobate, wide ovate or suborbicular | 5—11.5 (-20)×5.5—12 (-23) cm | Bifid to 1/10—1/3 (rarely 1/2); rounded at lobe apex | Deeply cordate | Pulvinate, rusty-pubescent or densely tomentous, 1.5—22 cm long | Chartaceous | Basal actinodromous, 9—11 in number, midvein apex spinose | Brochidodromous, 8—10 in number, none on the midvein |
| *Bauhinia humblotiana* Baill.  [= *Gigasiphon humblotianum* (Baill.) Drake] ^[3^, ^12]^ (LEGU) | Simple, unlobed, ovate to ovate-elliptic | (7-) 11—21×7—14 cm | Acuminate | Rounded or shallowly cordate | Pulvinate, slender, glabrous, 1.5—3.5 cm long | Chartaceous | Basal actinodromous, 5—7 in number | Eucamptodromous, 1 pair on the midvein |
| *Bauhinia krishnanunnii* A.K. Mathur et al. ^[28]^ (fossil LEGU) | Bilobate, wide ovate or suborbicular | 6×5 cm | Emarginate, U-shaped | Cordate | Not preserved | Coriaceous | Basal actinodromous, 7 in number | Eucamptodromous |
| *Bauhinia larsenii* D.X. Zhang et Y.F. Chen^[16, 36, this paper]^ (fossil LEGU) | Bilobate, suborbicular or slightly ovate to wide ovate | 2.1—4.5×1.8—4.8 cm | Bifid to ca. 1/2—3/5; rounded or obtuse at lobe apex | Rounded or shallowly cordate | Pulvinate, ca. 1—2.2 cm long | Apparently chartaceous | Basal actinodromous, 5—9 in number, midvein apex spinose | Brochidodromous |
| *Bauhinia marabarica* Roxb.  [= *Piliostigma marabaricum* (Roxb.) Benth.] ^[2, 8]^ (LEGU) | Bilobate, wide ovate or suborbicular | 5—12×8—16 cm | Bifid to 1/8—1/4; rounded at lobe apex | Rounded to subcordate | Pulvinate, grooved, densely pubescent, 2—4 cm long | Coriaceous | Basal actinodromous, 7—11 in number | Brochidodromous, 5—8 in number, none on the midvein |
| *Bauhinia nepalensis* N. Awasthi et N. Prasad^[29]^ (fossil LEGU) | Bilobate, each lobe ovate to elliptic | 4.5—7.5×2.8—5.7 cm | Emarginate; obtuse to rounded at lobe apex | Auriculate | Pulvinate, 3.8 cm long | Thickly chartaceous | Basal actinodromous, 11 in number | Eucamptodromous, about 4—6 pairs |
| *Bauhinia ningmingensis* Qi Wang et al. ^[36, this paper]^ (fossil LEGU) | Bifoliolate, each leaflet obliquely ovate or slightly falcate | 4.0—5.3×2.0—2.6 cm | Acutely obtuse | Wide cuneate or slightly concave | Pulvinate, petiole not preserved | Apparently membranous to chartaceous | Basal acrodromous, 3—4 in number per leaflet | Brochidodromous |
| *Bauhinia purpurea* L. ^[2, 3, 5—8, 17, 23, 36]^ (LEGU) | Bilobate, suborbicular | 10—15× 9—14 cm | Bifid to 1/3—1/2; slightly acute or rarely rounded at lobe apex | Shallowly cordate | Pulvinate, 3—4 cm long | Stiffly chartaceous | Basal actinodromous, 9—13 in number, midvein and outmost veins weak | Eucamptodromous, none on the midvein |
| *Bauhinia ramthiensis* Antal et N. Awasthi^[30]^ (fossil LEGU) | Bilobate, each lobe elliptic | Ca. 9.0×4.3 cm | Not preserved | Cordate | Not preserved | Coriaceous | Basal actinodromous, 9 in number | Eucamptodromous, numerous |
| *Bauhinia siwalika* U. Lakh. et N. Awasthi^[31]^ (fossil LEGU) | Bifoliolate, leaflet more or less ovate | 1.5—4.0×1—3 cm | Rounded | Auriculate | Pulvinate, thick, 0.8—1.0 cm long | Chartaceous | Basal actinodromous, 3—4 in per leaflet | Brochidodromous |
| *Bauhinia* sp. 1^[32 ]^(fossil LEGU) | Bilobate, wide ovate or orbicular | At least 3×7 cm | Bifid to 1/4; lobe apex missing | Cordate | Pulvinate, thick, 3.5 cm long | Coriaceous | Basal actinodromous, 9 in number | Incompletely preserved |
| *Bauhinia* sp. 2  (Cf. *Bauhinia purpurea* L.) ^[33]^ (fossil LEGU) | Bilobate, lobe ovate | 5.5×7.0 cm | Bifid to ca. 2/5; rounded at lobe apex | Approximately rounded | Not preserved | Coriaceous | Basal actinodromous, 9 in number | Brochidodromous |
| *Bauhinia thonningii* Schumach.  [= *Piliostigma thonningii* (Schumach.) Milne-Redh.] ^[3, 9, 15]^ (LEGU) | Bilobate, wide ovate | 5—17×6—19 cm | Bifid to 1/8—1/3; rounded at lobe apex | Cordate | Pulvinate, thick, 1.0—4.2 cm long | Coriaceous | Basal actinodromous, 7—11 in number, midvein apex spinose | Eucamptodromous or brochidodromous, numerous, 1—2 on the midvein |
| *Bauhinia variegata* L. [= *Phanera variegata* (L.) Benth.] ^[2, 3, 5—8, 17, 36]^ (LEGU) | Bilobate, suborbicular or wide ovate | 5—10×7—14 cm | Bifid to 1/3—1/2; rounded at lobe apex | Truncate or shallowly to deeply cordate | Pulvinate, 2.5—4 cm long | (Sub)coriaceous | Basal actinodromous, 9—11, rarely 13 in number, midvein apex spinose | Eucamptodromous, none on the midvein |
| *Bauhinia waylandii* R.W. Chaney^[34]^ (fossil LEGU) | Bilobate, suborbicular, lobe ovate | 2.5×2.5 cm | Bifid to 1/3; rounded at lobe apex | Broadly cordate | Pulvinate, stout, at least 1.3 cm long | Stiff | Basal actinodromous, 7 in number | Brochidodromous, slender |
| *Brenierea insignis* Humbert ^[2, 3, 12]^ (LEGU) | Bifoliolate, leaflet oblong or obovate to suborbicular | 0.6—1.2×0.35—0.9 (-1.2) cm (leaflet) | Rounded at leaflet apex | Cuneate | Grooved above, puberulous, densely scaly, 0.2—0.6 cm long | Somewhat thickened | Basal acrodromous, 3 or 4 in number per leaflet | Brochidodromous, none on the midvein |
| *Cercis canadensis* L. ^[2, 17, 22^, ^23, 36]^ (LEGU) | Simple, unlobed, suborbicular or wide ovate | 4—10 ×4—10 cm or slightly wider than long | Acuminate | Shallowly cordate | Pulvinate, 1.5—5.0 cm long | Chartaceous | Basal actinodromous, 5—7 in number | Brochidodromous, 2—4 pairs on the midvein |
| *Cercis chinensis* Bunge ^[2, 5, 6^, ^17, 24, 36]^ (LEGU) | Simple, unlobed, suborbicular or triangular-orbicular | 5—10 ×5—10 cm or slightly longer than wide | Acute | Shallowly to deeply cordate | Pulvinate, 1—4 cm long | Chartaceous | Basal actinodromous, 5—7 in number | Brochidodromous, 2—3 pairs on the midvein |
| *Cercis miochinensis* H.H. Hu et R.W. Chaney ^[24, 36]^ (fossil LEGU) | Simple, unlobed, suborbiculate or wide ovate | 3.3—13×2.7—11 cm | Acuminate | Wide cordate | Pulvinate, up to 2.9 cm long | Chartaceous | Basal actinodromous, 5—7 in number | Brochidodromous, 2—4 pairs on the midvein |
| *Colophospermum mopane* (Kirk ex Benth.) Kirk ex J. Léonard ^[3^, ^4, 10, 15]^ (LEGU) | Bifoliolate, butterfly-like, leaflet sub-triangular or obliquely ovate | 4.5—10×1.5—5.0 cm (leaflet) | Obtuse or obtusely acuminate at leaflet apex | Rounded or wide cuneate | Pulvinate, 1.8—2.5 cm long | Coriaceous | Basal actinodromous, numerous, 8—11 in number per leaflet | Brochidodromous, 4 in number per leaflet |
| *Cynometra bauhinaefolia* Benth. ^[4^, ^13]^ (LEGU) | Bifoliolate, leaflet obliquely ovate-oblong to ovate-lanceolate | 1—4×0.5—2.0 cm (leaflet) | Acuminate, with a retuse tip | Cuneate | Pulvinate, glabrous to densely pilose, 0.2—0.6 cm long | Thinly coriaceous | 2 in number, 1 per leaflet | Pinnate, brochidodromous, ca. 5—8 pairs per leaflet |
| *Cynometra elmeri* Merr. ^[11, 36]^ (LEGU) | Bifoliolate, leaflet (ob) ovate-oblong or oblong, somewhate falcate | 3.5—15×1.3—4.0 cm (leaflet) | Acuminate, with a retuse tip | Cuneate | Pulvinate, 0.4—0.8 cm long | Coriaceous | 2 in number, 1 per leaflet | Pinnate, brochidodromous, ca. 5—7 pairs per leaflet |
| *Griffonia physocarpa* Baill. ^[2, 3, 25]^ (LEGU) | Simple, alternate, unlobed, oblong-elliptic to ovate | 5—15×3—7 cm | Acuminate | Rounded | Pulvinate, ca. 0.5—1.0 cm long | Coriaceous | 1 midvein | Pinnate, brochidodromous, 4—5 pairs, including a strong, basal pair |
| *Guibourtia coleosperma* (Benth.) J. Léonard ^[4, 15]^ (LEGU) | Bifoliolate, butterfly-like, leaflet obliquely ovate | 7.0—10×3.8—4.8 cm (leaflet) | Obtusely acuminate | Obtuse or slightly cuneate | Pulvinate, 1.9—2.8 cm long | Coriaceous | 2 in number, 1 per leaflet | Pinnate, brochidodromous, ca. 5—7 pairs per leaflet, with 2—3 intersecondaries |
| *Hardwickia binata* Roxb. ^[3^, ^4, 10, 11, 17, 36]^ (LEGU) | Bifoliolate, butterfly-like, leaflet obliquely ovate or semi-orbicular | 2—6×0.7—3.3 cm (leaflet) | Obtusely acute | Obtuse | Pulvinate, slender, glabrous, 1.2—2.5 cm long | Coriaceous | Basal acrodromous, 4—6 per leaflet | Eucamptodromous, ca. 4—5 in number per leaflet |
| *Hymenaea verrucosa* Gaertn. ^[3^, ^5^, ^6, 12, 17, 36]^ (LEGU) | Bifoliolate, leaflet obliquely elliptic, curved | 5—9×2.5—4 cm (leaflet) | Shortly acuminate to obtuse | Obtuse, decurrent | Glabrous, 1.2—2.0 cm long; petiolule twisted, 0.1—0.3 cm long | Coriaceous | 2 in number, 1 per leaflet | Pinnate, brochidodromous, ca. 5—7 pairs per leaflet, with 2—3 intersecondaries |
| *Dilobeia thousarsii* Roem. et Schult.^[35]^ (PROT) | Bilobate, obovate | Ca. 12.5×11.3 cm | Bifid to 3/10; rounded at lobe apex | Concave, decurrent | Normal, ca. 7 cm long | Coriaceous | Suprabasal actinodromous, usually 3 in number, including 1 midvein, reaching the sinus of the apical notch | Pinnate, brochidodromous, including a pair on the midvein near the sinus |
| *Hoya kerrii* Craib  [= *Hoya obovata* Decaisne var. *kerrii* (Craib) Costantin.] ^[17, 36]^ (APOC) | Bilobate, obovate to orbicular | 4—6×5—6 cm | Retuse, shallowly emarginate | Rounded or wide cuneate | Robust, 0.5—2.0 cm long | Succulent, thick | 1 midvein | Pinnate, obscure |
| *Ipomoea* *pes-caprae* (L.) R. Br. ^[17, 36]^ (CONV) | Bilobate, ovate, elliptic, circular, reniform or somewhat quadrate to oblong | 3.5—9 × 3—10 cm | Bifid to 1/5—1/4 or emarginate; rounded at lobe apex | Wide cuneate, truncate, or shallowly cordate | Robust, 2—14 cm long | Succulent, rather thick, 2-glandular abaxially | 1 midvein, sometimes spinose at the apex | Pinnate, brochidodromous, 5—7 pairs, with 2—3 intersecondaries |
| *Liriodendron tulipifera* L. ^[17, 21, 36]^ (LIRD) | Commonly with 2 shallow upper lobes and 2 lateral lobes at broadest part, or sometimes squarrose and barely lobed | (4-) 7.5—15 (-23.5) × (8.5-) 12.5—18.5 (-25.5) cm | Broad truncate, emarginate or 2-lobed; acute or acuminate at lobe apex | Truncate to slightly cordate, sometimes with 1 lateral lobe near base of each side | Normal, 4—11.5 (-16) cm long | Membranous to chartaceous | 1 midvein | Pinnate, brochidodromous or craspedodromous 5—7 pairs |
| *Liriodendrites bradacii* K.R. Johnson ^[18, 19, 21]^ (fossil LIRD) | Bilobate, somewhat quadrate | 4—13 × 5—11 cm | Bifid to ca. 1/2—4/5; rounded or emarginate, rarely bilobate at lobe apex | Truncate to rounded, rarely acute or cordate | Normal, ca. 2—7 cm long | Unknown | 1 midvein, reaching the sinus of the apical notch | Pinnate, brochidodromous, 2—6 pairs |
| *Liriodendrites sachalinensis* (Krysht.) P.I. Alekseev ^[19, 21]^ (fossil LIRD) | Bilobate, orbicular or ovate | 7—9 × 8—9 cm | Bifid to 1/4—1/3; rounded or obtuse at lobe apex | Cordate | Not preserved | Unknown | 1 midvein, reaching the sinus of the apical notch | Pinnate, brochidodromous, 5—6 pairs |
| *Liriophyllum kansense* Dilcher et P.R. Crane ^[19—21]^ (fossil LIRD) | Bilobate, quadrate to wide ovate | 6—14 × 6.4—18.6 cm | Bifid to ca. 1/2—3/5; broadly rounded at lobe apex | Shallowly cordate, acute or obtuse, straight or more typically  decurrent | Stout, up to 10 cm long, simple, without alate appendages | Unknown | 1 midvein, dichotomizing  slightly before the leaf margin | Pinnate, brochidodromous, 2—5 pairs |
| *Oxalis corymbosa* DC. ^[17, 36]^ (OXAL) | Trifoliolate, leaflet obcordate | 1.0—4.5 × 1.5—6 cm  (leaflet) | Deeply emarginate | Wide cuneate | 5—30 cm long, with sparse or dense trichomes | Membranous | 1 midvein | Pinnate, brochidodromous, 2—3 pairs |
| *Passiflora* *cupiformis* Masters ^[17, 36]^ (PASS) | Bilobate, obovate or somewhat quadrate to oblong | 6—12 (-15) × 4 —10 cm | Bifid to 1/5—1/4 or truncate; rounded or obtuse at lobe apex | Rounded to cordate, truncate | 3—7 cm long, sparsely pubescent, with 2 discoid glands | Coriaceous | Basal actinodromous, usually 3 in number, rarely 5, midvein apex spinose | Brochidodromous, numerous, with a pair on the midvein |
| *Zygophyllum* *fabago* L. ^[17, 36]^ (ZYGO) | Bifoliolate, leaflet obovate to oblong-obovate | 1.5—3.3 × 0.6—2.0 cm (leaflet) | Rounded or obtuse | Cuneate | 1—3 cm long, grooved, with linear wings | Succulent, thick | 2 in number, 1 per leaflet | Pinnate, brochidodromous, ca. 2—4 in number |

**References**

1. Brummitt RK, Ross JH: **A reconsideration of the genus *Adenolobus* (Leguminosae–Caesalpinioideae).** Kew Bull 1976, 31:399–406.

2. Zhang DX: **Leaf venation of Cercideae (Leguminosae).** *J Trop Subtrop Bot* 1994, 2:45–57.

3. Lewis G, Forest F: **Tribe Cercideae.** In *Legumes of the world*. Edited by Lewis G, Schrire B, Mackinder B, Lock M. Kew: The Royal Botanic Gardens; 2005:57–68.

4. Herendeen PS, Jacobs BF: **Fossil legumes from the middle Eocene (46.0 Ma) Mahenge flora of Singida, Tanzania.** *Amer J Bot* 2000, 87:1358–1366.

5. Chen TC: ***Bauhinia* Linn.** In *Flora Reipublicae Popularis Sinicae, Tomus 39*. Edited by Wu TL, Chen PY, Wei CF, Chen TC. Beijing: Science Press; 1988:145–203.

6. Chen TC, Zhang DX, Larsen K, Larsen SS: ***Bauhinia* Linnaeus.** In *Flora of China, vol. 10*. Edited by Wu ZY, Raven PH, Hong DY. Beijing: Science Press & St. Louis: Missouri Botanical Garden Press; 2010:6–21.

7. Yu CH, Chen ZL: *Leaf architecture of the woody dicotyledons from tropical and subtropical China*. Beijing: Pergamon Press; 1991:1–414.

8. De Wit HCD: **A revision of Malaysian Bauhinieae.** *Reinwardtia* 1956, 3:381–539.

9. Brenan JPM: **Leguminosae subfamily Caesalpinioideae.** In *Flora of Tropical East Africa*. Edited by Milne-Redhead E, Polhill RM. London: Crown Agents for Oversea Governments and Administrations; 1967:1–230.

10. Welman M: ***Colophospermum mopane* is the correct name for the "mopane".** *SABONET News* 1999, 4:188–195.

11. Knaap van Meeuwen MS: **The Indo-Malesian and Pacific Cynometreae.** *Blumea* 1970, 18:13–53.

12. Du Puy DJ, Labat JN, Rabevohitra R, Villiers JF, Bosser J, Moat J: *The Leguminosae of Madagascar.* Kew: The Royal Botanic Gardens; 2002:1–737.

13. Dwyer JD: **The new world species of *Cynometra*.** *Ann Missours Bot Gard* 1958, 55:311–345.

14. Wieringa JJ: ***Monopetalanthus* exit. A systematic study of *Aphanocalyx*, *Bikinia*, *Icuria*, *Michelsonia* and *Tetraberlinia* (Leguminosae, Caesalpinioideae).** Wageningen Agric Univ Pap 1999, 99 (4):1–320.

15. Palmer E, Pitman N: *Trees of southern Africa, vol. 2*. Cape Town A. A. Balkema; 1972:705–1497.

16. Chen YF, Zhang DX: ***Bauhinia larsenii*, a fossil legume from Guangxi, China.** *Bot J Linn Soc* 2005, 147:437–440.

17. ***eFloras.org.*** Published on the Internet [http://www.efloras.org/] (accessed 15 April 2014). St. Louis: Missouri Botanical Garden & Cambridge: Harvard University Herbaria.

18. Johnson KR: **Description of seven common fossil leaf species from the Hell Creek Formation (Upper Cretaceous: Upper Maastrichtian), North Dakota, South Dakota, and Montana.** *Proc Denver Mus Nat Hist, Ser 3*, 1996, 12:1–47.

19. Alekseev PI: **Genus *Liriodendrites* in Cretaceous and Early Paleogene floras of northern Asia.** *Paleontol J* 2009, 43:1181–1189.

20. Dilcher DL, Crane PR: ***Archaeanthus*: An early angiosperm from the Cenomanian of the western Interior of North America.** *Ann Missouri Bot Gard* 1984, 71:351–384.

21. Romanov MS, Dilcher DL: **Fruit structure in Magnoliaceae *s. l.* and Archaeanthus and their relationships.** *Amer J Bot* 2013, 100:1494–1508.

22. Owens SA, Fields PF, Ewers FW: **Degradation of the upper pulvinus in modern and fossil leaves of *Cercis* (Fabaceae).** *Amer J Bot* 1998, 85:273–284.

23. Owens SA: **Secondary and tertiary pulvini in the unifoliate leaf of *Cercis canadensis*** L. (Fabaceae) with comparison to *Bauhinia purpurea* L. *Int J Pl Sci* 2000, 161:583–597.

24. Wang Q: **Pulvini of *Cercis* leaves from the Miocene Shanwang Formation of Shandong Province and the early evolution of the pulvinus in Leguminosae.** *Acta Palaeontol Sin* 2012, 51:1–13.

25. Aubréville A: **Légumineuses-Caesalpinioidées.** In *Flore du Gabon, vol. 15.* Aubréville A, Leroy JF. Paris: Muséum Nationale d’Histoire Naturelle; 1968:1–362.

26. Calvillo-Canadell L, Cevallos-Ferriz SRS: ***Bauhcis moranii* gen. et sp. nov. (Cercideae, Caesalpinioideae), an Oligocene plant from Tepexi de Rodríguez, Puebla, Mexico, with leaf architecture similar to *Bauhinia* and *Cercis*.** *Rev Palaeobot Palynol* 2002, 122:171–184.

27. Berry EW: **Fossil floras from southern Ecuador.** *Johns Hopkins Univ Stud Geol* 1945, 14:93–150.

28. Mathur AK, Mishra VP, Mehra S: **Systematic study of plant fossils from Dagshai, Kasauli and Dharmsala formations of Himachal Pradesh.** *Geol Surv India, Palaeontol Indica, N S* 1996, 50:1–121.

29. Awasthi N, Prasad M: **Siwalik plant fossils from Surai Khola area, western Nepal.** *Palaeobotanist* 1990, 38:298–318.

30. Antal JS, Awasthi N: **Fossil flora from the Himalayan foot-hills of Darjeeling District, West Bengal, India and its palaeoecological and phytogeographical significance.** *Palaeobotanist* 1993, 42:14–60.

31. Lakhanpal RN, Awasthi N: **A late Tertiary forule from near Bhikhnathoree in West Champaran District, Bihar.** In *Proceedings of the symposium on evolutionary botany and biostratigraphy. Current Trends in Life Sciences 10 (A. K. Ghosh Commemoration Volume)*. Edited by Sharma AK, Mitra GC, Banerjee M. New Delhi: Today and Tomorrow’s Printers & Publishers; 1984: 587–596.

32. Endo S, Fujiyama I: **Some Late Mesozoic and Late Tertiary plants and a fossil insect from Thailand.** In *Contributions to the geology and palaeontology of Southeast Asia 31, geology and palaeontology of Southeast Asia, volume 2*. Edited by Kobayashi T, Toriyama R. Tokyo: University of Tokyo Press; 1966:191–197.

33. Bande MB, Srivastava GP: **Late Cenozoic plant-impressions from Mahuadnar Valley, Palamu District, Bihar.** *Palaeobotanist* 1990, 37:331–366.

1. Chaney RW: **A Tertiary flora from Uganda.** *J Geol* 1933, 41:702–709.

35. Pole M, Bowman DMJS: **Tertiary plant fossils from Australia’s ‘Top End’**. *Austral Syst Bot* 1996, 9:113–126.

36. Direct observations on the cultivated plants and the specimens deposited at Chengdu Institute of Biology, Chinese Academy of Sciences (CDBI), Guangxi Institute of Botany, Chinese Academy of Sciences (IBK), South China Botanical Garden, Chinese Academy of Sciences (IBSC), the Herbarium of Northeast China (IFP), Kunming Institute of Botany, Chinese Academy of Sciences (KUN), Natural History Museum of Guangxi (NHMG), and the Chinese National Herbarium (PE) (see Additional file 2).
