# Supplementary material for: Leaves and fruits of Bauhinia (Leguminosae, Caesalpinioideae, Cercideae) from the Oligocene Ningming Formation of Guangxi, South China and their biogeographic implications
Source: BMC Evol Biol. 2014 Apr 24;14:88. doi: 10.1186/1471-2148-14-88 (PMC4101841; doi:10.1186/1471-2148-14-88)
Supplement: Additional file 4 — Previously described fossils assignable or similar to Bauhinia L. [file 1471-2148-14-88-S4.doc]

**Additional file 4 Previously described fossils assignable or similar to *Bauhinia* L.**

| **Name** | **Locality** | **Organ** | **Cited figure and specimen** | **Geological age** | **Age reference** | **Identification** |
| --- | --- | --- | --- | --- | --- | --- |
| *Liriophyllum populoides* Lesq.  (= *Liriophyllum beckwithii* Lesq.) | Kassler sandstone, South Platte Formation near Morrison, Colorado, USA | Leaf | Lesquereux, 1878, p. 482–483, no figure; Lesquereux, 1883, p. 75–76, pl. 10, fig. 1, pl. 11, figs. 1–2; Hollick, 1894, p. 471, pl. 221; Hollick, 1896, p. 249, pl. 269, fig. 2; Dilcher and Crane, 1984, p. 367, figs. 47–50 | Albian–Cenomanian, late Early Cretaceous–early Late Cretaceous | Dilcher and Crane, 1984 | Closely related to Liriodendraceae *sensu lato* M.S. Romanov et Dilcher, see Romanov and Dilcher, 2013, p. 1504 |
| *Liriophyllum kansense* Dilcher et P.R. Crane | Harker, Dakota Formation, Kansas, USA | Leaf | Dilcher et al., 1976, p. 855, fig. 1e, f; Retallack and Dilcher, 1981, p. 44, fig. 2. 14; Dilcher and Crane, 1984, p. 367, figs. 51–59, 60b; Romanov and Dilcher, 2013, p. 1504, fig. 7B | Albian–Cenomanian, late Early Cretaceous–early Late Cretaceous | Dilcher and Crane, 1984 | Closely related to Liriodendraceae *sensu lato*, see Romanov and Dilcher, 2013, p. 1504 |
| *Liriodendron giganteum* Lesq. | Fort Harker and Glasco, Kansas, USA | Leaf | Lesquereux, 1874, p. 93, pl. 22, fig. 2; Lesquereux, 1883, p. 74, no figure | Albian–Cenomanian, late Early Cretaceous–early Late Cretaceous | Dilcher and Crane, 1984 | Possibly related to *Liriophyllum* Dilcher et P.R. Crane (Liriodendraceae *sensu lato*) |
| *Liriodendrites occidentalis* P.I. Alekseev | Antibes, Sym Formation, western Siberia, Russia | Leaf | Alekseev, 2009, p. 1188, pl. 1, figs. 1–2, pl. 2, figs. 1–3 | Coniacian, Late Cretaceous | Alekseev, 2009 | Closely related to Liriodendraceae *sensu lato* |
| *Liriophyllum sachalinense* Krysht.  [*Bauhinia sachalinensis* (Krysht.) Vakhram.  (*nomen nudum*)] | Mgachi, Sakhalin Island, Russia | Leaf | Kryshtofovich, 1937, p. 272, pl. 10, figs. 4, 5; Vakhrameyev, 1966, p. 80, no description and figure; Krassilov, 1979, p. 118, pl. 46, figs. 3–5, pl. 47, figs. 1–3; Alekseev, 2009, p. 1185, fig. 2a, b | Santonian, Late Cretaceous | Alekseev, 2009 | Reclassified into an extinct genus *Liriodendrites* K.R. Johnson as *Liriodendrites* *sachalinensis* (Krysht.) P.I. Alekseev, see Alekseev, 2009, p. 1185 |
| *Bauhinia kazakhstanica* Shilin | Chu-Sarysu Depression, Taldysaj, Kazakhstan | Leaf | Shilin, 1970, p. 58, pl. 1, fig. 6; Shilin and Romanova, 1978, p. 67, pl. 17, figs. 1, 3 | Santonian–Campanian, Late Cretaceous | Shilin and Romanova, 1978; Krassilov et al., 1983 | Rejected as *Bauhinia* L. here due to lacking the basal actinodromous venation and upper pulvinus; possibly related to Liriodendraceae *sensu lato* |
| *Liriodendron iijimae* Tanai | Sawayama Formation, Hikage, Kuji City, Iwate Prefecture, Japan | Leaf | Tanai, 1979, p. 103, pl. 10, figs. 1, 3, 5, text-fig. 3. 7 | Campanian, Late Cretaceous | Tanai, 1979 | Bearing the pinnate secondary veins; closely related to *Liriodendrites* (Liriodendraceae *sensu lato*) |
| *Liriodendrites bradacii* K.R. Johnson | Hell Creek Formation near Marmarth, Slope County, North Dakota, USA | Leaf | Johnson, 1996, p. 3, figs. 1–14; Romanov and Dilcher, 2013, p. 1504, fig. 7B | Maastrichtian, Late Cretaceous | Johnson, 1996 | Closely related to Liriodendraceae *sensu lato* |
| *Liriodendrites aeternus* (Golovneva) P.I. Alekseev | Koryak Upland, Rarytkin, Koryak, and Kakanaut Formations, Russia | Leaf | Golovneva, 1994, p. 108, pl. 7, figs. 1, 6, pl. 72, fig. 5; Alekseev, 2009, p. 1188, fig. 2c, d | Maastrichtian, Late Cretaceous | Alekseev, 2009 | Closely related to Liriodendraceae *sensu lato* |
| *Liriodendropsis* *simplex* Newb. | Raritan Formation, Woodbridge, New Jersey, USA | Leaf | Newberry, 1895, p. 83, pl. 19, figs. 2–3, pl. 53, figs. 1–4, 7 | Late Cretaceous | Newberry, 1895 | Related to *Liriodendron* L., see Newberry, 1895, p. 83; recombined into a legume fossil genus as *Dalbergites simplex* (Newb.) Seward, see Seward and Conway, 1935, p. 26 |
| *Bauhinia cretacea* Newb. | Raritan Formation, Woodbridge, New Jersey, USA; Mgachi, Sakhalin Island, Russia | Leaf | Newberry, 1886, p. 77, pl. 56, fig. 5; Berry, 1911, p. 162, pl. 19, fig. 3; Kryshtofovich, 1918, p. 53, fig. 10; Kryshtofovich, 1937, p. 275, fig. 7; Krassilov, 1979, p. 118, pl. 46, figs. 3–5, 8, pl. 47, figs. 1–3 | Late Cretaceous | Berry, 1919; Kryshtofovich, 1937; Alekseev, 2009 | Rejected as *Bauhinia* by Herendeen et al, 1992, p. 305 and the present authors due to lacking of basal actinodromous venation and upper pulvinus. Newberry’s  *Bauhinia cretacea* appears to be related to *Liriodendrites*; Kryshtofovich’s *Bauhinia cretacea* has been reclassified as *Liriodendrites* *sachalinensis*, see Alekseev, 2009, p. 1185 |
| *Bauhinia ? gigantea* Newb. | Raritan Formation, Woodbridge, New Jersey, USA | Leaf | Newberry, 1895, p. 93, pl. 20, fig. 1; Berry, 1911, p. 164, no figure | Late Cretaceous | Berry, 1911 | Rejected as *Bauhinia* by Herendeen et al, 1992, p. 305 and the present authors. It bears the pinnate venation and lacks the upper pulvinus |
| *Bauhinia alabamensis* E.W. Berry | Tuscaloosa, Hale County, Alabama, USA | Leaf | Berry, 1910, p. 256, fig. 1; Berry, 1919, p. 99, pl. 23, fig. 8, text-fig. 12 | Late Cretaceous | Berry, 1919 | Rejected as *Bauhinia* by Herendeen et al, 1992, p. 305 and the present authors; bearing suprabasal, actinodromous venation and 3 shallow, lateral lobes, it is unrelated to *Bauhinia* |
| *Bauhinia marylandica* E.W. Berry | Magothy Formation at Grove Point, Maryland, USA | Leaf | Berry, 1908, p. 219, figs. no number; Berry, 1919, p. 98, no figure | Late Cretaceous | Berry, 1919 | Rejected as *Bauhinia* by Herendeen et al, 1992, p. 305 and the present authors; bearing the pinnate venation, it is related to *Liriodendrites* (Liriodendraceae *sensu lato*) |
| *Bauhinia ripleyensis* E.W. Berry | Ripley Formation, Barbour County, Alabama; McNairy sand member, McNairy County, Tennessee, USA | Leaf | Berry, 1916a, p. 294, pl. 16, fig. 1; Berry, 1919, p. 100, pl. 23, fig. 7 | Late Cretaceous | Berry, 1919 | Rejected as *Bauhinia* by Herendeen et al, 1992, p. 305 and the present authors; bearing the suprabasal actinodromous venation, it is unrelated to *Bauhinia* |
| *Bauhinia* sp. | Beleuty River, Kazakhstan | Leaf | Shilin, 1977, p. 138, pl. 5, fig. 6 | Late Cretaceous | Shilin, 1977 | Rejected as *Bauhinia* here due to lacking of actinodromous venation and upper pulvinus |
| *Cercis eocenica* Lesq. | Laramie Formation, Erie, Colorado, USA | Leaf | Lesquereux, 1873, p. 384, no figure; Schimper, 1874, p. 614, no figure; Knowlton, 1922, p. 148, no figure | Late Cretaceous | Knowlton, 1922 | Unidentifiable |
| *Phyllites bipartitus* Velen. | Bohemia, the Czech Republic | Leaf | Velenovský, 1885, p. 12, pl. 6, fig. 4 | Late Cretaceous | Berry, 1908 | Unrelated to *Bauhinia* here due to lacking an upper pulvinus; bearing a dichotomous midvein reaching the sinus of the apical notch, it may belong to *Liriophyllum* Lesq. (Liriodendraceae *sensu lato*) |
| *Liriodendrites sachalinensis* (Krysht.) P.I. Alekseev | Arkovo Formation, Mgachi, Sakhalin Island; Boshnyakovo Formation, Avgustovka River, Russia | Leaf | Kryshtofovich, 1937, p. 272, pl. 10, figs. 4, 5; Krassilov, 1979, p. 118, pl. 46, figs. 3–5, pl. 47, figs. 1–3; Alekseev, 2009, p. 1185, fig. 2a, b | Santonian, Late Cretaceous–Danian, early Paleocene | Alekseev, 2009 | Closely related to Liriodendraceae *sensu lato* |
| *Dalbergites simplex* (Newb.) Seward | Atanikerdluk, western Greenland, Denmark | Leaf | Newberry, 1895, p. 83, pl. 19, figs. 2–3, pl. 53, figs. 1–4, 7; Seward and Conway, 1935, p. 26, text-figs. 22–24 | Early Paleocene | Koch, 1964 | Closely similar to an extant species *Amicia zygomeris* DC. (Leguminosae), see Seward and Conway, p. 27 |
| *Bauhinites groenlandica* Seward | Atanikerdluk, western Greenland, Denmark | Leaf | Seward and Conway, 1935, p. 25, text-fig. 21 | Early Paleocene | Koch, 1964 | Unidentifiable for an affinity with *Bauhinia* due to lacking the base |
| *Bauhinia gracilis* J.R. Tao | Wuyun, Jiayin County, Heilongjiang Province, China | Leaf | Tao and Xiong, 1986, p. 127, pl. 13, fig. 6; Tao et al., 2000, p. 169, pl. 16, fig. 6 | Paleocene | Feng et al., 2000 | Rejected as *Bauhinia* here; bearing the crenate margin with glandular teeth, it may belong to *Populus* L. (Salicaceae Mirb.) |
| *Bauhinia wyomingiana* R.W. Br. | Fort Union Formation, Monarch, Wyoming, USA | Leaf | Brown, 1956, p. 104, fig. 1; Brown, 1962, p. 74, pl. 43, fig. 10 | Paleocene | Brown, 1962 | Rejected as *Bauhinia* by Herendeen et al, 1992, p. 305 and the present authors due to its pinnate venation; possibly related to *Liriodendrites* (Liriodendraceae *sensu lato*) |
| *Bauhinia* sp.  (*nomen nudum*) | Lance, near Ranchester, Wyoming, USA | Leaf | Knowlton, 1909, p. 211, no description and figure | Paleocene | Brown, 1962 | Unidentifiable |
| *Cercis borealis* Newb. | Yellowstone R. Valley, Montana, USA | Leaf | Newberry, 1883, p. 510, no figure | Paleocene | LaMotte, 1952 | Reclassified as *Paranymphaea crassifolia* (Newb.) R.W. Br., see Brown, 1962, p. 70, pl. 36, figs. 1–5 |
| *Cercis truncata* Lesq. | Badlands, North Dakota, USA | Leaf | Lesquereux, 1883, p. 237, no figure | Paleocene | LaMotte, 1952 | Unidentifiable |
| *Cercis coloradensis* Knowlton | Dawson Arkose, Colorado Springs, Colorado, USA | Leaf | Knowlton, 1930, p. 99, pl. 45, fig. 5 | Paleocene | LaMotte, 1952 | Reclassified as *Cercidiphyllum ellipticum* (Newb.) R.W. Br., see Brown, 1939, p. 491 |
| *Cercis ?* sp. | Ping Chau Island, Hong Kong, China | Leaf | Lee et al., 1991a, p. 52; Lee et al., 1991b, p. 43, no description and figure | Late Paleocene–Eocene | Lee et al., 1991b | Unidentifiable |
| *Cercis* sp. | Raichikha, Amur Region, Russia | Fruit | Baikovskaya, 1986, p. 6, pl. 2, fig. 10, pl. 3, figs. 1, 2. | Early Eocene | Akhmetiev, 2010 | Doubtful; due to lacking the wing along a placental suture |
| *Cercis* sp. | Laguna del Hunco, Chubut, Argentina | Leaf | Calvillo-Canadell et al., 2007, p. 585, no description and figure | Late Paleocene–Middle Eocene | Wilf et al., 2005; Calvillo-Canadell et al., 2007 | Rejected here (no adequate characters assignable to living *Cercis*, Personal communication with Dr. Peter Wilf, Pennsylvania State University, 2014) |
| *Cercis wilcoxiana* E.W. Berry | Holly Springs Sand, Vaughns, near Lamar, Benton County, Mississippi, USA | Leaf | Berry, 1916b, p. 228, pl. 49, fig. 1 | Early Eocene | Berry, 1916b | Rejected and reclassified as *Cercidiphyllum arcticum* (Heer) R.W. Br., see Brown, 1939, p. 492 |
| *Bauhinia primigenia* Ettingsh. et J.S. Gardner (*nomen nudum*) | London Clay, Sheppey, UK | Seed | Ettingshausen, 1879, p. 396, no description and figure | Early Eocene | Collinson and Cleal, 2001 | Reclassified into Icacinaceae Miers as *Faboidea crassicutis* Bowerb., see Reid and Chandler, 1933, p. 341 |
| *Bauhinia europaea* Engelh. | Messel, Darmstadt, Germany | Leaf | Engelhardt, 1922, p. 115, pl. 39, fig. 4 | Middle Eocene | Collinson et al., 2012 | Rejected as *Bauhinia* here due to its pinnate venation; possibly related to *Liriodendrites* (Liriodendraceae *sensu lato*) |
| *Cercis deperdita* Watelet | Belleu, Paris Basin, France | Leaf | Watelet, 1866, p. 241, pl. 58, fig. 9 | Middle Eocene | Kvaček, 2010 | Rejected by Wang, 2012; due to lacking of actinodromous venation |
| *Cercis dubia* Watelet | Belleu, Paris Basin, France | Leaf | Watelet, 1866, p. 242, pl. 59, fig. 1 | Middle Eocene | Kvaček, 2010 | Rejected by Wang, 2012; due to lacking of actinodromous venation |
| *Cercis grandifolia* Engelh. | Messel, Darmstadt, Germany | Leaf | Engelhardt, 1922, p. 114, pl. 38, fig. 1 | Middle Eocene | Collinson et al., 2012 | Rejected by Kvaček and Wilde, 2010; reclassified as *Byttneriopsis spiegelii* (Engelh.) Kvaček et Wilde (Malvaceae Juss.), see Kvaček and Wilde, 2010, p. 172 |
| *Bauhinia* sp. | Mahenge site, Singida Town, Tanzania | Leaf | Jacobs and Herendeen, 2004, p. 119, fig. 4Q | Middle Eocene | Herendeen and Jacobs, 2000; Harrison et al., 2001 | Doubtful (in the original figure of Jacobs and Herendeen, 2004, the preservation appears too poor to reliably assign this leaf to *Bauhinia*). Although “lobed leaf with pulvinus at base of lamina, fan-like main veins radiating from the base” was stated by Bruneau et al., 2008, p. 701, no figures were illustrated in 2008 |
| *Aphanocalyx singidaensis* Herend. et B.F. Jacobs | Mahenge site, Singida Town, Tanzania | Leaf | Herendeen and Jacobs, 2000, p. 1359, figs. 2–6 | Middle Eocene | Herendeen and Jacobs, 2000; Harrison et al., 2001 | Assigned to the tribe Amherstieae Benth. (Caesalpinioideae, Leguminosae), see Herendeen and Jacobs, 2000, p. 1359 |
| Cf. *Cynometra* sp. | Mahenge site, Singida Town, Tanzania | Leaf | Herendeen and Jacobs, 2000, p. 1361, figs. 13–15 | Middle Eocene | Herendeen and Jacobs, 2000; Harrison et al., 2001 | Very similar to some genera in the tribe Amherstieae, see Herendeen and Jacobs, 2000, p. 1362 |
| *Bauhinia pseudocotyledon* Cockerell | Florissant, Colorado, USA | Leaf | Cockerell, 1909, p. 184, fig. no number | Late Eocene | Meyer and Smith, 2008; Wang et al., 2013 | Rejected as *Bauhinia* here due to its pinnate venation |
| *Bauhinia* sp. | Na Duong Formation, Na Duong coal mine, northern Vietnam | Leaf | Böhme et al., 2013, p. 151, fig. 26B, no description | Late Eocene (late Bartonian–Priabonian) | Böhme et al., 2013 | Rejected as *Bauhinia* here due to its pinnate venation and narrow leaf form |
| *Cercis herbmeyeri* H. Jia et Manchester | John Day Formation, Teater Road, Oregon, USA | Fruit | Jia and Manchester, 2014, fig. 4A–M, in press | Late Eocene | Manchester and McIntosh, 2007 | Reliable; bearing the wing along a placental suture |
| *Cercis parvifolia* Lesq. | Florissant, Colorado and Teater Road, Oregon, USA; Red Deer River, Alberta, Canada; Messel, Darmstadt, Germany; Csillaghegy, Budapest, Hungary | Leaf and fruit (no organic connections) | Lesquereux, 1883, p. 201, pl. 31, figs. 5–7; Penhallow, 1908, p. 44, no figure; Engelhardt, 1922, p. 114, pl. 38, fig. 2; Brown, 1937, p. 177, pl. 54, figs. 2, 5; Rásky, 1943, p. 527, pl. 24, fig. 1; MacGinitie, 1953, p. 124, pl. 43, fig. 1, pl. 44, figs. 1–3, pl. 45, fig. 5; Becker, 1969, p. 103, pl. 32, fig. 6–8; Becker, 1972, p. 41, pl. 10, fig. 1–9; Jia and Manchester, 2014, figs. 2–3 | Middle Eocene–early Oligocene | Hably, 2006; Meyer and Smith, 2008; Collinson et al., 2012; Wang et al., 2013 | Leaf from Hungary reclassified as *Kydia kraeuselii* (Rásky) Hably, see Hably, 2010, p. 412; Some reliable leaf fossils from Colorado and Oregon belong to *Cercis parvifolia* Lesq. while fruits are given a new name *Cercis herbmeyeri* H. Jia et Manchester, see Jia and Manchester, 2014 |
| *Cercis nipponica* (Tanai) Tanai | Higure-zawa, Ikushunbetsu, Mikasa City, Hokkaido, Japan; Primorye Region, Russia | Leaf | Tanai, 1981, p. 474, pl. 8, figs. 8–10, text-fig. 4, k, l; Pavlyutkin, 2007, p. 578, pl. 11; Pavlyutkin and Petrenko, 2010, p. 45 | Late Eocene–Early Oligocene | Tanai, 1981; Pavlyutkin, 2007; Pavlyutkin and Petrenko, 2010 | Doubtful; petioles not preserved, see Jia and Manchester, 2014 |
| *Cercis tenuinervis* (Lesq.) R.W. Br. | Green River, DeBeque, Piceance Creek, Colorado, USA | Leaf | Brown, 1934, p. 58, pl. 12, figs. 1–4 | Eocene | LaMotte, 1952 | Doubtful |
| *Cercis* sp. | Fushun, Liaoning Province, China | Leaf | Onuki, 1931, p. 2, no description and figures; Hu and Chaney, 1938, p. 51 | Eocene | Onuki, 1931 | Unidentifiable |
| *Bauhinia* aff. *B. divaricata* L. | Itaquaquecetuba, São Paulo Basin, Brazil | Leaf | Biagolini et al., 2013, p. 644, fig. 4A, C | Late Eocene–Early Miocene | Biagolini et al., 2013 | Doubtful; poorly preserved and no pulvinus |
| *Bauhinia wadii* E.W. Berry | Lagrange Formation, Hickman, Fulton County, Kentucky, USA | Leaflet | Berry, 1924, p. 169, pl. 51, fig. 3 | Eocene | LaMotte, 1952 | Rejected as *Bauhinia* here due to its dichotomous and then sub-parallel secondary veins; it may not belong to Leguminosae |
| *Cercis hungarica* Rásky | Csillaghegy, Budapest, Hungary | Leaf | Rásky, 1943, p. 528, pl. 24, fig. 2, 4; Hably, 1986, p. 35, pl. 2, fig. 4 | Early Oligocene | Hably, 2006, 2010 | Reclassified as *Kydia* *kraeuselii* (Rásky) Hably (Malvaceae L.), see Hably, 2010, p. 412 |
| *Cercis harmatii* Rásky | Szépvölgy, Budapest, Hungary | Fruit | Rásky, 1943, p. 519, pl. 16, fig. 3 | Early Oligocene | Hably, 2006 | Doubtful |
| *Cercis maurerae* Meyer et Manchester | Iron Mountain, Painted Hills, Crooked River, Oregon, USA | Fruit | Meyer and Manchester, 1997, p. 123, pl. 49, figs. 8–10 | Oligocene | Meyer and Manchester, 1997 | Reliable |
| *Cercis oregonensis* Chaney | Eagle Creek, Multnomah and Hood River Cos., Oregon, USA | Leaf | Chaney, 1920, p. 177, pl. 16, figs. 3, 4 | Oligocene | LaMotte, 1952 | Reclassified as *Exbucklandia oregonensis* (Chaney) R. W. Br., see Brown, 1946, p. 348 |
| *Cercis* sp. | Gray Ranch, Crooked R., Oregon, USA | Fruit | Chaney, 1927, p. 125, pl. 15, fig. 5 | Oligocene | LaMotte, 1952 | Assigned to *Cercis spokanensis* Knowlton by LaMotte, 1952, p. 121; similar to *Cercis maurerae* Meyer et Manchester, 1997, p. 123 |
| *Cercis* sp. | John Day Formation, Fossil, Oregon, USA | Fruit | Manchester and Meyer, 1987, p. 123, fig. 6B | Oligocene | Manchester and Meyer, 1987 | Reclassified as *Cercis maurerae* Meyer et Manchester, 1997, p. 123 |
| *Cercis* sp. | Caijiachong Formation, Jinggu, Yunnan Province, China | Fruit | WGCPC, 1978, p. 105, pl. 87, fig. 4 | Oligocene | Guo and Zhou, 1992; Jia and Manchester, 2014 | Reliable |
| *Bauhinia* sp. | Kumarhatti, Kalka-Shimla Highway, Solan District, Himachal Pradesh, India | Leaf | Mathur et al., 1996, p. 33, pl. 5, fig. 2 | Oligocene | Srivastava and Guleria, 2006 | Unidentifiable; lacking the base and apex of the leaflet |
| *Bauhinia* *larsenii* D.X. Zhang et Y.F. Chen | Ningming County, Guangxi Zhuang Autonomous Region, China | Leaf and fruit (with organic connections) | Chen and Zhang, 2005, p. 439, figs. 1–5; Specimens: NHMG 45003, 45004, 45012, and 45019 | Oligocene | Chen and Chang, 2011; Shi et al., 2012 | Reliable; the same locality as that of the fossils in this study |
| *Bauhcis moranii* Calvillo-Canadell et Cevallos-Ferriz | Los Ahuehuetes, Tepexi de Rodríguez, Puebla, Mexico | Leaf | Calvillo-Canadell and Cevallos-Ferriz, 2002, p. 173, fig. 3 | Oligocene | Calvillo-Canadell and Cevallos-Ferriz, 2002 | Reliable for assigning to the tribe Cercideae; attributed to *Bauhinia* by Chen and Zhang, 2005, p. 437 |
| *Cercis antiqua* Saporta | Aix-en-Provence, southern France; Aydin, Şahinali Province, Turkey; Ipolytarnóc, Hungary; Mainz-Kastel, Hessen, Germany; Kundratec, Czech Republic | Leaf and fruit (no organic connections) | Saporta, 1862, p. 287, pl. 14, fig. 4A, B; Saporta, 1873, p. 123, pl. 17, figs. 7–15; Schimper, 1874, p. 373, pl. 105, figs. 6, 6a; Saporta, 1877, p. 405, fig. 3. 1, 2; Saporta, 1879, p. 240, fig. 53. 1, 2; Fritel, 1903, p. 223, fig. 266; Menzel, 1897, p. 10, pl. 1, fig. 8; Brabenec, 1909, p. 214, no figure; Jablonszky, 1915, p. 279; Kräusel, 1938, p. 63, pl. 9, fig. 9, pl. 10, figs. 1–4, text-fig. 19a–e; Mädler and Steffens, 1979, p. 13, pl. 6, fig. 3 | Oligocene–Early Miocene | Kirchheimer, 1957; Mädler and Steffens, 1979; Châteauneuf and Nury, 1995; Nury, 2000; Kvaček, 2004; Hably, 2006 | Reliable for those records from France; doubtful for those records from Turkey and Germany; rejected that record from Czech Republic |
| *Cercis ameliae* Saporta | Bois d'Asson, southern France | Leaf and fruit (no organic connections) | Saporta, 1867, p. 117, pl. 14, figs. 10–12; Schimper, 1874, p. 373, no figure | Oligocene | Châteauneuf and Nury, 1995; Nury, 2000 | Rejected here for leaves; doubtful for fruits |
| *Cercis spokanensis* Knowlton | Latah Formation, Whitebird, Idaho, and Spokane, Washington, USA; Csillaghegy, Budapest, Hungary | Leaf and fruit (no organic connections) | Knowlton, 1926, p. 43, pl. 29, fig. 9; LaMotte, 1936, p. 132, pl. 10, fig. 4; Brown, 1937, p. 177, pl. 54, figs. 8–12; Rásky, 1943, p. 529, pl. 24, fig. 3 | Early Oligocene–Middle Miocene | Owens et al., 1998; Hably, 2006 | Some leaves transferred to *Vitis* *washingtonensis* (Knowlton) R. W. Br. (Vitaceae) or *Exbucklandia* *oregonensis* (Chaney) R. W. Br. (Hamamelidaceae)*,* see Brown, 1937, p. 181, 1946, p. 348; leaf from Hungary rejected here and possibly belongs to *Disanthus* Maxim.; fruits reclassified as *Caesalpinia spokanensis* (Knowlton) Herend. et Dilcher, see Herendeen and Dilcher, 1991, p. 8 |
| *Cercis virgiliana* Massalongo  (*Cercis* "*virgilianum*") | Sinigaglia and Santa Giustina, Liguria, Italy | Leaf and fruit (no organic connections) | Massalongo, 1858, p. 129; Massalongo and Scarabelli, 1859, p. 425, pl. 9, figs. 20, 21; Meschinelli and Squinabol, 1892, p. 461; Paolucci, 1896, p. 147, pl. 24, fig. 179; Principi, 1916, p. 175, pl. 77, figs. 6–8, pl. 85, fig. 1 | Early Oligocene–Miocene | Bonci et al., 2011 | Leaves reclassified into *Kydia* Roxb. as *K. kraeuseli* (Rásky) Hably (Malvaceae L.), see Hably, 2010, p. 412; fruits assignable to *Cercis* are waiting for reconfirmation |
| *Cercis tournoueri* Saporta | Brognon, France; Salcedo, Italy | Leaf | Saporta, 1866, p. 273, pl. 1, fig. 6, text-fig. G, H; Schimper, 1874, p. 372, no figure; Schimper and Schenk, 1890, p. 683, figs, 6, 7; Principi, 1926, p. 99, pl. 11, fig. 2 | Middle Oligocene–Miocene | Principi, 1926 | Rejected here; due to bearing strong pinnate veins |
| *Cercis turgaica* Usnadze (later homonym) | Sarybulak, Kazakhstan; Krynka River, Alexandrovka Village, near the northern coast of the Azov Sea, southern Ukraine | Leaf and fruit (no organic connections) | Usnadze, 1957, p. 226, pl. 6, fig. 6, text-fig. 2; Takhtajan et al., 1963, p. 706, pl. 28, fig. 2; Kryshtofovich and Baikovskaya, 1965, p. 86, 87, pl. 19, fig. 10, pl. 21, fig. 6, pl. 22, fig. 14, pl. 39, fig. 2, text-figs. 32, 33 | Late Oligocene–Middle Miocene | Zhilin et al., 1989; Denk et al., 2011 | Reliable for some leaf fossils; being a later homonym of *Cercis turgaica* Usnadze ex Kiritchkova, 1955, it has been renamed as *Cercis kryshtofovichii* Usnadze, see Usnadze, 1971, p. 970 |
| *Cercis* sp. | Dembi Bay, Sikhote-Alin, southern Primorye, Russia | Leaf | Akhmetiev et al., 1973, p. 169, no description and figure | Possibly Late Oligocene | Guo and Zhang, 2002 | Unidentifiable |
| cf. Cercideae / Detarieae | Lightning Ridge, the Murray–Darling Basin, New South Wales, Australia | Leaf | Carpenter et al., 2011, p. 410, figs. 58–64 | Possibly latest Oligocene–middle-late Miocene | Carpenter et al., 2011 | Doubtful; lacking the upper pulvinus and bearing strong secondary veins on the mid-vein; Possibly belonging to Proteaceae Juss., cf. *Dilobeia* L.M.A.A. Du Petit-Thouars or Liriodendraceae*, Liriodendrites* as Pole and Bowman (1996), Pole (1998) formerly thought for those similar leaves from the Van Diemen Sandstone of Melville Island, Northern Territory, Australia |
| *Bauhinia kachchhensis* R.N. Lakh. et Guleria | Khari Nadi Bed, near Goyela-Mokra; Kachchh, India | Leaf | Lakhanpal and Guleria, 1982, p. 283, pl. 2, figs. 10, 12, 13, text-fig. 4; Lakhanpal et al., 1984, p. 251, pl. 11, figs. 1, 2, 4, text-fig. 12 | Early Miocene | Lakhanpal and Guleria, 1982 | Doubtful; lacking the base and apex of the foliage |
| *Bauhinia kasaulica* R. Arya et N. Awasthi | Kasauli, Himachal Pradesh, India | Leaf | Arya and Awasthi, 1994, p. 60, figs. 2, 3 | Early Miocene | Arya and Awasthi, 1994 | Doubtful; being 7–8 primary veins per lobe, it may not belong to *Bauhinia*, which usually bears 3–6 primary veins per lobe |
| *Bauhinia krishnanunnii* A.K. Mathur et al. | Dagshai Cantonment and Daghota, Kalka-Shimla Highway, Solan District, Himachal  Pradesh, India | Leaf | Mathur et al., 1996, p. 46, pl. 14, fig. 5; Guleria et al., 2000, 45, pl. 1, fig. 3 | Early Micoene | Srivastava and Guleria, 2006 | Reliable; similar to living *Bauhinia variegata* L. |
| *Bauhinia* sp. | Mae Sot, Changwat Tak, Thailand | Leaf | Endo and Fujiyama, 1966, p. 192, pl. 8, fig. 3 | Late Early Miocene–early Middle Miocene | Ridd et al., 2011 | Reliable; similar to *Bauhinia* *bidentata* Jack [= *Phanera bidentata* (Jack) Benth.], see Endo and Fujiyama, 1966, p. 192 |
| *Bauhinia* sp. | Chiang Muan basin, northern Thailand | Pollen | Songtham et al., 2004, p. 179, no description and figures | Late Middle Miocene | Songtham et al., 2004 | Unidentifiable |
| *Bauhinia destructa* Unger | Radoboj, Croatia | Fruit | Unger, 1850, p. 493; Unger, 1864, p. 31, pl. 11, fig. 2; Schimper, 1874, p. 389, no figure | Middle Miocene | Wang et al., 2010 | Legume fossil fruits with uncertain affinities; reclassified into *Leguminocarpum* Dotzler herein, see Wang, 2012a, p. 873 |
| *Bauhinia parschlugiana* Unger | Seegraben and Parschlug, Austria | Fruit | Unger, 1850, p. 493; Unger, 1864, p. 31, pl. 11, fig. 3; Schimper, 1874, p. 389, no figure; Ettingshausen, 1888, p. 366, no figure | Middle Miocene | Kovar-Eder et al., 2004 | Recombined as *Leguminosites parschlugianus* (Unger) Kovar-Eder et Z. Kvaček by Kovar-Eder et al., 2004, p. 74; reclassified as *Leguminocarpum parschlugianum* herein |
| *Bauhinia germanica* Heer | Oeningen, Kesselstein, Germany | Leaf | Heer, 1857, p. 109, pl. 134, fig. 21; Schimper, 1874, p. 389, no figure | Middle Miocene | Wang et al., 2013 | Rejected here due to its strong pinnate venation in lobes |
| *Bauhinia ramthiensis* Antal et N. Awasthi | Right bank of upsteam of Ramthi River near Oodlabari, Darjeeling District, West Bengal, India | Leaf | Antal and Awasthi, 1993, p. 32, pl. 9, fig. 1 | Middle Miocene | Srivastava and Guleria, 2006 | Reliable; similar to living *Bauhinia acuminata* L. |
| *Bauhinium palaeomalabaricum* U. Prakash et M. Prasad | Kalagarh, Pauri Garhwal District, Uttar Pradesh; Oodlabari, Darjeeling District, West Bengal, India | Wood | Prakash and Prasad, 1984, p. 140, pl. 1, figs. 1–3, pl. 2, figs. 5–8; Antal et al., 1996, p. 99, pl. 1, figs 1–4 | Middle Miocene | Prakash and Prasad, 1984; Srivastava and Guleria, 2006 | Similar to living *Bauhinia malabarica* Roxb., see Prakash and Prasad, 1984, p. 141 |
| *Bauhinia miocenica* R.C. Mehrotra et al. | Subansiri riverbeds in Dhemaji, Dhemaji District, Assam, India | Wood | Mehrotra et al., 2011, p. 687, fig. 5a–h | Middle Miocene–Late Miocene | Mehrotra et al., 2011 | Similar to fossil *Bauhinium palaeomalabaricum* and living *Bauhinia malabarica* Roxb., see Mehrotra et al., 2011, p. 689 |
| *Bauhinia siwalika* U. Lakh. et N. Awasthi | Siwalik, Bhikhnathoree, West Champaran District, Bihar; Cherrapunji, West Khasi Hills District, Meghalaya, India | Leaf and leaflet | Lakhanpal and Awasthi, 1984, p. 590, pl. 1, figs. 8–10; Ambwani, 1991, p. 142, fig. 1D | Middle Miocene–middle Pleistocene | Prasad et al., 2004; Srivastava and Guleria, 2006 | Reliable; similar to living *Bauhinia corymbosa* Roxb. ex DC., *B. diptera* Blume ex Miq., *B. hookerii* F. Muell., and *B. tomentosa* L., see Lakhanpal and Awasthi, 1984, p. 591 |
| *Bauhinia tertiara* N. Awasthi et R.C. Mehrotra | Naginimara Village, Kongan Coalfield, Mon District, Nagaland; Champanagar, Agartala District, Tripura, India | Wood | Awasthi and Mehrotra, 1990, p. 278, pl. 1, figs. 3, 8, pl. 2, figs. 1–3; Sen and Bera, 2005, p. 68, pl. 2, figs. 3, 4, 6 | Late Miocene | Srivastava and Guleria, 2006 | Similar to living *Bauhinia racemosa* Lam., see Awasthi, 1992, p. 243 |
| *Bauhinia olympica* Unger | Kyme, Euboea Island, Greece | Leaf | Unger, 1867, p. 61, pl. 15, fig. 36; Schimper, 1874, p. 390, no figure | Miocene | Unger, 1867 | Rejected as *Bauhinia* here due to its suprabasal actinodromous venation |
| *Bauhinia ecuadorensis* E.W. Berry | Loja Basin, Ecuator | Leaf | Berry, 1945, p. 127, pl. 8, fig. 10 | Miocene | **Menéndez, 1969;** Burnham, 1995 | Reliable; a nice bifoliolate leaf of *Bauhinia* |
| *Bauhinia* sp.  (*nomen nudum*) | Santa Ana, Columbia | Leaf | Engelhardt, 1895, p. 47, pl. 9, fig. 12, no description | Miocene | Berry, 1929; **Menéndez, 1969** | Unidentifiable |
| *Bauhinia* sp. | Oguni-Machi, Uzen Province, Japan | Leaf | Morita, 1932, p. 2, no description and figure | Miocene | Morita, 1932 | Unidentifiable |
| *Cercis miochinensis* H.H. Hu et R.W. Chaney | Shanwang Basin, Linqu County, Shandong Province, China | Leaf and fruit (no organic connections) | Hu and Chaney, 1938, p. 51, pl. 26, figs. 1, 3–5, pl. 27, fig. 5; WGCPC, 1978, p. 105, pl. 89, figs. 4, 5, pl. 90, fig. 1, pl. 91, fig. 1, pl. 93, figs. 1, 6; Wang, 2012b, p. 2, pl. 1, figs. 1–8, pl. 2, fig. 1; Specimens: PE 50610, 50648, 51995, 52907, 990002, 20110509, 20110604 | Miocene | Wang, 2012c; Wang et al., 2013 | Reliable for the pulvinate leaves of *Cercis* L., see Wang, 2012b; fruits assignable to *Cercis* are waiting for reconfirmation |
| *? Bauhinia* sp. | Tyrrell, Washington, USA | Leaf | Smiley, 1963, p. 234, pl. 17, fig. 9 | Late Miocene–early Pliocene | Smiley, 1963 | Doubtful; superficially similar to a leaflet of *Bauhinia*, see Smiley, 1963, p. 234 |
| *Bauhinia thonningii* Schumach. | Nkondo Formation (Albertine Group), Nkondo, Uganda | Wood | Dechamps and Ergo, 1994, p. 43, no description and figure | Late Miocene–Early Pliocene | Dechamps and Ergo, 1994; Pickford and Senut, 1994 | Similar to an extant species *Bauhinia thonningii* Schumach., see Dechamps and Ergo, 1994, p. 43 |
| *Bauhinia deomalica* N. Awasthi et R.C. Prakash | Namsang Beds, Deomali, Arunachal Pradesh; Neyveli Lignite Mine-I, South Arcot District, Tamil Nadu, India | Wood | Awasthi and Prakash, 1987, p. 179, pl. 2, figs. 2–6; Agarwal, 1991, p. 119, figs. 1, 2 | Miocene–Pliocene | Awasthi and Prakash, 1987; Srivastava and Guleria, 2006 | Similar to an extant species *Bauhinia foveolata* Dalzell, see Awasthi, 1992, p. 242 |
| *Bauhinium miocenicum* Trivedi et Panjwani | Sukhasot, Kalagarh, Pauri Garhwal District, Uttar Pradesh, India | Wood | Trivedi and Panjwani, 1986, p. 66, pl. 1, figs. A–E | Miocene–Pliocene | Trivedi and Panjwani, 1986 | Similar to an extant species *Bauhinia retusa* Roxb., see Awasthi, 1992, p. 242 |
| *Bauhinia nepalensis* N. Awasthi et N. Prasad | Surai Khola beds, near Surai Khola bridge, Surai Khola, Nepal | Leaf | Awasthi and Prasad, 1990, p. 309, pl. 5, figs. 6–8 | Late Miocene–late Pleistocene | Sanyal and Sinha, 2010 | Reliable; similar to living *Bauhinia malabarica* Roxb. and *B. variegata* L., see Awasthi and Prasad, 1990, p. 309 |
| *Bauhinia potosiana* E.W. Berry | Silver and Tin District, Potosi, Bolivia | Leaf | Berry, 1917, p. 144, pl. 17, figs. 1, 2 | Pliocene | Berry, 1917 | Rejected as *Bauhinia* here due to lacking the basal acrodromous or actinodromous venation |
| *Bauhinia waylandii* R.W. Chaney | Busano, Bugishu District, Eastern Province, Uganda | Leaf | Chaney, 1933, p. 706, fig. 1 | Pliocene | Willis, 1933 | Reliable; similar to living *B. macrantha* Oliv. [= *Bauhinia petersiana* Bolle subsp. *macrantha* (Oliv.) Brummitt et J.H. Ross], see Chaney, 1933, p. 706 |
| *Bauhinia emarginata* Mill.  (= *Bauhinia aculeata* L.) | Gatun Lake, Panama | Pollen | Bartlett and Barghoorn, 1973, p. 262, pl. 4, fig. 1; Graham, 1992, p. 162, fig. 9 | Quaternary | Graham, 1992 | Assigned to living *Bauhinia emarginata* Mill. by Bartlett and Barghoorn, 1973, p. 262 |
| *Bauhinia* sp.  (Cf. *Bauhinia purpurea* L.) | Mahuadanr Valley, Palamu District, Bihar, India | Leaf | Bande and Srivastava, 1990, p. 348, pl. 6, fig. 1 | Late Cenozoic | Bande and Srivastava, 1990 | Reliable; apparently a folded leaf of *Bauhinia* |

**References**

Agarwal A: **Occurrence of *Altingia* and *Bauhinia* in the Neyveli Lignite (Miocene), India.** *J Indian Bot Soc* 1991, 70:119–121.

Akhmetiev MA: **Paleocene and Eocene floristic and climatic change in Russia and northern Kazakhstan.** *Bull Geosci* 2010, 85:77–94.

Akhmetiev MA, Bratzeva GM, Klimova RS: **The age analogues of the *Engelhardtia* beds of Korea and Primorye.** *Dok Akad Nauk SSSR* 1973, 209:167–170.

Alekseev PI: **Genus *Liriodendrites* in Cretaceous and Early Paleogene floras of northern Asia.** *Paleontol J* 2009, 43:1181–1189.

Ambwani K: **Leaf impressions belonging to the Tertiary age of North-East India.** *Phytomorphology* 1991, 41:139–146.

Antal JS, Awasthi N: **Fossil flora from the Himalayan foot-hills of Darjeeling District, West Bengal, India and its palaeoecological and phytogeographical significance.** *Palaeobotanist* 1993, 42:14–60.

Antal JS, Prasad M, Khare EG: **Fossil woods from the Siwalik sediments of Darjeeling District, West Bengal, India.** *Palaeobotanist* 1996, 43:98–105.

Arya R, Awasthi N: **A new species of *Bauhinia* from the Kasauli Formation (Lower Miocene), Kasauli, Himachal Pradesh.** *Geophytology* 1994, 24:59–62.

Awasthi N: **Indian fossil legumes.** In *Advances in legume systematics, part 4: the fossil record*. Edited by Herendeen PS, Dilcher DL. Kew: The Royal Botanic Gardens; 1992:225–250.

Awasthi N, Mehrotra RC: **Some fossil woods from Tipam Sandstone of Assam and Nagaland.** *Palaeobotanist* 1990, 38:277–284.

Awasthi N, Prakash U: **Fossil woods of *Kingidendron* and *Bauhinia* from the Namsang beds of Deomali, Arunachal Pradesh.** *Palaeobotanist* 1987, 35:178–183.

Awasthi N, Prasad M: **Siwalik plant fossils from Surai Khola area, western Nepal.** *Palaeobotanist* 1990, 38:298–318.

Baikovskaya TN: **New plant species from Lower Tertiary sediments of the Raichikha locality in the Amur region.** In *Problems of Paleobotany*, edited by Takhtajan AL. Leningrad: Nauka; 1986:5–12.

Bande MB, Srivastava GP: **Late Cenozoic plant-impressions from Mahuadnar Valley, Palamu District, Bihar.** *Palaeobotanist* 1990, 37:331–366.

Bartlett AS, Barghoorn ES: **Phytogeographic history of the Isthmus of Panama during the past 12,000 years (A history of vegetation, climate, and sea-level change).** In *Vegetation and vegetational history of northern Latin America*. Edited by Graham A. Amsterdam: Elsevier Scientific Publishing Company; 1973:203–299.

Becker HF: **Fossil plants of the Tertiary Beaverhead Basins in southwestern Montana.** *Palaeontogr Abt B* 1969, 127:1–142.

Becker HF: **The Metzel Ranch flora of the Upper Ruby River Basin, Southwestern Montana.** *Palaeontogr Abt B* 1972, 141:1–61.

Berry EW: **A new Cretaceous *Bauhinia*.** *Torreya* 1908, 8:218–219.

Berry EW: **A new Cretaceous *Bauhinia* from Alabama.** *Amer J Sci, Ser* 4, 1910, 29:256–258.

Berry EW: The flora of the Raritan Formation. *Bull Geol Surv New Jersey* 1911, 3:1–233.

Berry EW: **Contributions to the Mesozoic flora of the Atlantic Coastal Plain, XI. Tennessee.** *Bull Torrey Bot Club* 1916a, 43:283–304.

Berry EW: **The Lower Eocene floras of southeastern North America.** *U S Geol Surv Profess Pap* 1916b, 91:1–481.

Berry EW: **Fossil plants from Bolivia and their bearing upon the age of uplift of the eastern Andes.** *Proc U S Natl Mus* 1917, 54:103–164.

Berry EW: **Upper Cretaceous floras of the eastern gulf region in Tennesse, Mississippi, Alabama, and Georgia.** *U S Geol Surv Profess Pap* 1919, 112:1–177.

Berry EW: **The Middle and Upper Eocene floras of southeastern North America.** *U S Geol Surv Profess Pap* 1924, 92:1–206.

Berry EW: **Tertiary fossil plants from Columbia, South America.** *Proc U S Natl Mus* 1929, 75:1–12.

Berry EW: **Fossil floras from southern Ecuador.** *Johns Hopkins Univ Stud Geol* 1945, 14:93–150.

Biagolini CH, Bernardes-de-Oliveira MEC, Caramês AG: **Itaquaquecetuba Formation, São Paulo basin, Brazil: new angiosperm components of Paleogene Taphoflora**. *Brazil J Geol 2013*, 43:639–652.

Böhme M, Aiglstorfer M, Antoine P-O, Appel E, Havlik P, Métais G, Phuc LT, Schneider S, Setzer F, Tappert R, Tran DN, Uhl D, Prieto J: **Na Duong (northern Vietnam)–an exceptional window into Eocene ecosystems from Southeast Asia.** *Zitteliana A*, 2013, **53**:121–167.

Bonci MC, Vannucci G, Tacchino S, Piazza M: **Oligocene fossil leaves of the Perrando collection: history, preservation, and paleoclimatic meaning.** *Boll Soc Paleontol Italiana* 2011, 50:145–164.

Brabenec B: **Souborná květena českého útvaru třetihorního.** *Archív pro Přírodovědecké Prozkoumání Čech* 1909, 14:1–374.

Brown RW: **The recognizable species of the Green River flora.** *U S Geol Surv Profess Pap* 1934, 185-C:45–77.

Brown RW: **Additions to some fossil floras of the western United States.** *U S Geol Surv Profess Pap* 1937, 186-J: 163–206.

Brown RW: **Fossil leaves, fruits, and seeds of *Cercidiphyllum*.** *J* *Paleontol* 1939, 13:485–499.

Brown RW: **Alterations in some living and fossil floras.** *J Washington Acad Sci* 1946, 36:344–355.

Brown RW: **New items in Cretaceous and Tertiary floras of the western United States.** *J Washington Acad Sci* 1956, 46:104–108.

Brown RW: **Paleocene flora of the Rocky Mountains and Great Plains.** *U S Geol Surv Profess Pap* 1962, 375:1–119.

Bruneau A, Mercure M, Lewis GP, Herendeen PS: **Phylogenetic patterns and diversification in the caesalpinioid legumes.** *Botany* 2008, 86:697–718.

Burnham RJ: **A new species of winged fruit from the Miocene of Ecuador: *Tipuana ecuatoriana* (Leguminosae).** *Amer J Bot* 1995, 82:1599–1607.

Calvillo-Canadell L, Cevallos-Ferriz SRS: ***Bauhcis moranii* gen. et sp. nov. (Cercideae, Caesalpinioideae), an Oligocene plant from Tepexi de Rodríguez, Puebla, Mexico, with leaf architecture similar to *Bauhinia* and *Cercis*.** *Rev Palaeobot Palynol* 2002, 122:171–184.

Calvillo-Canadell L, Grandolfo MA, Zamaloa MC, Cuneo RN, Wilf P, Johnson K: **Legume fossils from the early Eocene Laguna del Hunco paleoflora, Chubut, Patagonia, Argentina.** Paper No. 216-5. *Geol Soc Amer Abs Prog* 2007, 39:585 [available at <https://gsa.confex.com/gsa/2007AM/finalprogram/abstract_130641.htm>].

Carpenter RJ, Goodwin MP, Hill RS, Kanold K: **Silcrete plant fossils from Lightning Ridge, New South Wales: new evidence for climate change and monsoon**

**elements in the Australian Cenozoic.** *Austral J Bot* 2011, 59: 399–425.

Chaney RW: **The flora of Eagle Creek Formation.** *Contributions from Walker Museum* 1920, 2:115–181.

Chaney RW: **Additions to the paleontology of the Pacific Coast and Great Basin regions of North America 4. Geology and paleontology of the Crooked River Basin, with special reference to the Bridge Creek flora.** *Carnegie Inst Washington Publ* 1927, 346:45–138.

Chaney RW: **A Tertiary flora from Uganda.** *J Geol* 1933, 41:702–709.

Châteauneuf J-J, Nury D: **The flora of Oligocene of southern Province: stratigraphic, environmental and climatic implications.** *Géol France* 1995, 2:43–55.

Chen GJ, Chang MM: **A new cyprinin from Oligocene of South China.** *Sci China Earth Sci* 2011, 54:481–492.

Chen YF, Zhang DX: ***Bauhinia larsenii*, a fossil legume from Guangxi, China.** *Bot J Linn Soc* 2005, 147:437–440.

Cockerell TDA: **Two new fossil plants from Florissant, Colorado.** *Torreya* 1909, 9:184–185.

Collinson ME, Cleal CJ: **Early and early–middle Eocene (Ypresian–Lutetian) palaeobotany of Great Britain.** In *Mesozoic and Tertiary palaeobotany of Great Britain. Geological Conservation Review Series, No. 22.* Cleal CJ, Thomas BA, Batten DJ, Collinson ME. Peterborough: Joint Nature Conservation Committee; 2001:185–226

Collinson ME, Manchester SR, Wilde V: **Fossil fruits and seeds of the middle Eocene Messel biota, Germany.** *Abh Senckenberg Ges Naturf* 2012, 570:1–251.

Dechamps R, Ergo A: **Palaeovegetation (fossil plants) of the Albertine rift valley.** In *Geology and palaeobiology of the Albertine Rift Valley, Uganda*–*Zaire, vol. 2: Palaeobiology*. Edited by Senut B, Pickford M. *CIFEG Occasional Publication, Orléans* 1994*,* 29:29–45.

Denk T, Grímsson F, Zetter R, Símonarson LA: *Late Cainozoic floras of Iceland*. *Topics in Geobiology 35.* Dordrecht, Heidelberg, London & New York: Springer; 2011:1–854.

Dilcher DL, Crane PR: ***Archaeanthus*: An early angiosperm from the Cenomanian of the western Interior of North America.** *Ann Missouri Bot Gard* 1984, 71:351–384.

Dilcher DL, Crepet WL, Becker CD, Reynolds HC: **Reproductive and vegetative morphology of a Cretaceous angiosperm.** *Science* 1976, 191:854–856.

Endo S, Fujiyama I: **Some Late Mesozoic and Late Tertiary plants and a fossil insect from Thailand.** In *Contributions to the geology and palaeontology of Southeast Asia 31, geology and palaeontology of Southeast Asia, volume 2*. Edited by Kobayashi T, Toriyama R. Tokyo: University of Tokyo Press; 1966:191–197.

Engelhardt H: **Über neue Tertiärpflanzen Süd-Amerikas.** *Abh Senckenberg Naturf Ges* 1895, 19:1–47.

Engelhardt H: **Die Alttertiäre Flora von Messel bei Darmstadt.** *Abh Hess Geol Landesanst Darmstadt* 1922, **7**:17–128.

Ettingshausen CF: **Report on phyto-palaeontological investigation of the fossil flora of Sheppey.** *Proc Roy Soc London* 1879, 29:388–396.

Ettingshausen CF: **Die fossile Flora von Leoben in Steiermark 2.** *DenkschrKaiserl Akad Wiss, Wien Math -Naturwiss Kl* 1888, 54:319–384.

Feng GP, Wang YF, Li CS: **Reconsideration of the geological age of the Wuyun Formation, Heilongjiang Province, China.** *Chin Bull Bot* 2000, 17 (Spec issue):74–83.

Fritel P-H: *Histoire Naturelle de la France 24e bis Partie Paléobotanique (Plantes Fossiles).* Les Fils D'Émile Deyrolle; 1903:1–379.

Golovneva LB: **Maastrichtian–Danian floras of the Koryak Upland.** *Proc Komarov Bot Inst Russ Acad Sci* 1994, 13:1–147.

Graham A: **The current status of the legume fossil record in the Caribbean region.** In *Advances in legume systematics, part 4: the fossil record*. Edited by Herendeen PS, Dilcher DL. Kew: The Royal Botanic Gardens; 1992:161–167.

Guleria JS, Srivastava R, Prasad M: **Some fossil leaves from the Kasauli Formation of Himachal Pradesh, North-West India.** *Himalayan Geol* 2000, 21:43–52.

Guo SX, Zhang GF: **Oligocene Sanhe flora in Longjing County of Jilin, Northeast China.** *Acta Palaeontol Sin* 2002, 41:193–210.

Guo SX, Zhou ZK: **The megafossil legumes from China.** In *Advances in legume systematics, part 4: the fossil record.* Edited by Herendeen PS, Dilcher DL. Kew: The Royal Botanic Gardens; 1992:207–223.

Hably L: **The macroflora of the borehole Kiscell-1 in Budapest.** *Ann Hist-Nat Mus Natl Hungar* 1986, 78:31–40.

Hably L: **Catalogue of the Hungarian Cenozoic leaf, fruit and seed floras from 1856 to 2005.** *Stud. Bot. Hung.* 2006, 37:41–129.

Hably L: **The Early Oligocene Flora of Santa Giustina (Liguria, Italy)–Revision and comparison with the flora of the Tard Clay Formation.** *Riv Italiana Paleontol Stratigr* 2010, 116:405–420.

Harrison T, Msuya CP, Murray AM, Jacobs BF, Báez AM, Mundil R, Ludwig KR: **Paleontological investigations at the Eocene locality of Mahenge in North-Central Tanzania, East Africa.** In *Eocene biodiversity: Unusual occurrences and rarely sampled habitats.* Edited by Gunnell GF. New York: Kluwer Academic/Plenum Publishers; 2001:39–74.

Heer O: *Die Tertiäre Flora der Schweiz, Flora Tertiaria Helvetiae, vol. 3 (6).* Winterthur: Verlag der lithographischen Anstalt von Wurster & Comp.; 1857:1–200.

Herendeen PS, Crepet WL, Dilcher DL: **The fossil history of the Leguminosae: phylogenetic and biogeographic implications.** In *Advances in legume systematics, part 4, the fossil record*. Edited by Herendeen PS, Dilcher DL. Kew: The Royal Botanic Gardens; 1992:303–316.

Herendeen PS, Dilcher DL: ***Caesalpinia* subgenus *Mezoneuron* (Leguminosae, Caesalpinioideae) from the Tertiary of North America.** *Amer J Bot 1991,* 78:1–12.

Herendeen PS, Jacobs BF: **Fossil legumes from the middle Eocene (46.0 Ma) Mahenge flora of Singida, Tanzania.** *Amer J Bot* 2000, 87:1358–1366.

Jia H, Manchester SR: **Fossil leaves and fruits of *Cercis* (Leguminosae) from the Eocene of western North America.** *Int J Pl Sci* 2014, 175, in press.

Hollick A: **Wing-like appendages on the petioles of *Liriophyllum populoides* Lesq. and *Liriodendron alatum* Newb., with description of the latter.** *Bull Torrey Bot Club* 1894, 21:467–471.

Hollick A: **Appendages to the petioles of *Liriodendra*.** *Bull Torrey Bot Club* 1896, 23:249–250.

Hu HH, Chaney RW: **A Miocene flora from Shantung Province, China, part 1. Introduction and systematic considerations.** *Carnegie Inst Washington Publ* 1938, 507:1–82.

Jablonszky E: **Die Mediterrane Flora von Tarnóc.** *Mitteil Jahr Königlich-Ungar Geol Reich* 1915, 22:249–293.

Jacobs BF, Herendeen PS: **Eocene dry climate and woodland vegetation in tropical Africa reconstructed from fossil leaves from northern Tanzania.** *Palaeogeogr, Palaeoclimat, Palaeoecol* 2004, 213:115–123.

Jia H, Manchester SR: **Fossil leaves and fruits of *Cercis* (Leguminosae) from the Eocene of western North America.** *Int J Pl Sci* 2014, 175, in press.

Johnson KR: **Description of seven common fossil leaf species from the Hell Creek Formation (Upper Cretaceous: Upper Maastrichtian), North Dakota, South Dakota, and Montana.** *Proc Denver Mus Nat Hist, Ser 3*, 1996, 12:1–47.

Kirchheimer F: *Die Laubgewächse der Braunkohlenzeit.* Halle (Saale): Veb Wilhelm Knapp Verlag; 1957:1–783.

Kiritchkova AI: **Flora of the upper Indrikoteri Series near Akmole (= The flora of the upper part of the Indricotherian Suite of Akmola).** *Mater Istorii Fauny Fl Kazakhstana* 1955, 1:138–150.

Knowlton FH: **The stratigraphic relations and paleontology of the "Hell Creek Beds", "Ceratops Beds" and equivalents, and their reference to the Fort Union Formation.** *Proc Washington Acad Sci* 1909, 11:179–238.

Knowlton FH: **The Laramie flora of the Denver Basin, with a review of the Laramie problem.** *U S Geol Surv Profess Pap* 1922, 130:1–175.

Knowlton FH: **Flora of Latah Formation of Spokane, Washington, and Coeur D’Alene, Idaho.** *U S Geol Surv Profess Pap* 1926, 140:17-119.

Knowlton FH: **The flora of the Denver and associated formations of Colorado.** *U S Geol Surv Profess Pap* 1930, 155:1–142.

Koch BE: **Review of fossil floras and nonmarine deposits of West Greenland.** *Geol Soc Amer Bull* 1964, 75:535–548.

Kovar-Eder J, Kvaček Z, Ströbitzer-Hermann M: **The Miocene flora of Parschlug (Styria, Austria)—Revision and synthesis.** *Ann Naturhist Mus Wien* 2004, 105A:45–157.

Krassilov VA: *The Cretaceous flora of Sakhalin.* Moscow: Nauka; 1979:1–184.

Krassilov VA, Shilin PV, Vachrameev VA: **Cretaceous flowers from Kazakhstan.** *Rev Palaeobot Palynol* 1983, 40:91–113.

Kräusel R: **Die Tertiäre Flora der Hydrobienkalke von Main-Kastel.** *Palaeontol Zeit* 1938, 20:9–103.

Kryshtofovich AN: **On the Cretaceous flora of Russian Sakhalin.** *J Coll Sci Imp Univ Tokyo* 1918, 40:1–73.

Kryshtofovich AN: **Cretaceous flora of Sakhalin, Mgach and Polovinka.** In *A. N. Kryshtofovich selected works, Tom 2, 1962 (reprinted).* Moscow & Leningrad: Izdatel’stvo Academii Nauk SSSR; 1937:211–289.

Kryshtofovich AN, Baikovskaya TN: *Sarmatian flora of Krinka.* Moscow: Akademii Nauk SSSR, Botanical Institute of Komarov; 1965:1–134.

Kvaček Z: **Revisions to the Early Oligocene flora of Flörsheim (Mainz Basin, Germany) based on epidermal anatomy.** *Senckenberg Lethaea* 2004, 84:1–73.

Kvaček Z: **Forest flora and vegetation of the European early Palaeogene–a review.** *Bull Geosci* 2010, 85:63–76.

Kvaček Z, Wilde V: **Foliage and seeds of malvalean plants from the Eocene of Europe.** *Bull Geosci* 2010, 85:163–182.

LaMotte RS: **Middle Cenozoic floras of western North America 5. The upper Cedarville flora of northwestern Nevada and adjacent California.** *Carnegie Inst Washington Publ* 455:57–142.

LaMotte RS: **Catalogue of the Cenozoic plants of North America through 1950.** *Geol Soc Amer Mem* 1952, 51:1–381.

Lakhanpal RN, Awasthi N: **A late Tertiary forule from near Bhikhnathoree in West Champaran District, Bihar.** In *Proceedings of the symposium on evolutionary botany and biostratigraphy. Current Trends in Life Sciences 10 (A. K. Ghosh Commemoration Volume)*. Edited by Sharma AK, Mitra GC, Banerjee M. New Delhi: Today and Tomorrow’s Printers & Publishers; 1984: 587–596.

Lakhanpal RN, Guleria JS: **Plant remains from the Miocene of Kachchh, western India.** *Palaeobotanist* 1982, 30:279–296.

Lakhanpal RN, Guleria JS, Awasthi N: **The fossil floras of Kachchh-III Tertiary megafossils.** *Palaeobotanist* 1984, 33:228–319.

Lee CM, Atherton MJ, Wu SQ, He GX, Chen JH, Nau PS: Discovery of angiosperm fossils from Hong Kong, with discussion on the age of the Ping Chau Formation. *Geol Soc Hong Kong Newsletter* 1991a, 9:50–60.

Lee CM, Chen JH, He GX, Atherton MJ, Lai KW: On the age of the Ping Chau Formation. *Geol Soc Hong Kong Newsletter* 1991b, 9:34–49.

Lesquereux L: **Lignitic Formation and fossil flora.** *Ann Rep U S Geol Geogr Surv Territ* 1873, 6:317–427.

Lesquereux L: **Contributions to the fossil flora of the western territories, part 1. The Cretaceous flora.** *Ann Rep U S Geol Surv Territ* 1874, 6:1–136.

Lesquereux L: **Remarks on specimens of Cretaceous and Tertiary plants secured by the survey in 1877, with a list of the species hitherto described.** *Ann Rep U S Geol Geogr Surv Territ* 1878, 10:481–520.

Lesquereux L: **Contributions to the fossil flora of the western territories, part 3. The Cretaceous and Tertiary floras.** *Ann Rep U S Geol Surv Territ* 1883, 8:1–283.

Mädler K, Steffens P: **Neue Blattfloren aus dem Oligozän Neogen und Pleistozän der Türkei (= New finds of fossil leaves from Oliogcene, Neogene, and Pleistocene deposits of Turkey).** *Geol Jahr, Reih Region Geol Ausland* 1979, 33:3–33.

Manchester SR, Meyer HW: **Oligocene fossil plants of the John Day Formation, Fossil, Oregon.** *Oregon Geol* 1987, 49:115–127.

Manchester SR, McIntosh WC: **Late Eocene silicified fruits and seeds from the John Day Formation near Post, Oregon.** *PaleoBios* 2007, 27:7–17.

Massalongo AB: *Synopsis Florae Fossilis Senogalliensis*. Veronae: Apud A. Merlo; 1858:1–136.

Massalongo AB, Scarabelli EG: *Studii sulla Flora Fossile e Geologia Stratigrafica del Senigalliese.* Imola: Tipografia D’Ignazio Galeati e Figlio; 1859:1–504.

Mathur AK, Mishra VP, Mehra S: **Systematic study of plant fossils from Dagshai, Kasauli and Dharmsala formations of Himachal Pradesh.** *Geol Surv India, Palaeontol Indica, N S* 1996, 50:1–121.

MacGinitie HD: **Fossil plants of the Florissant beds, Colorado.** *Carnegie Inst Washington Publ* 1953, 599:1–198.

Mehrotra RC, Bera SK, Basumatary SK, Srivastava G: **Study of fossil wood from the Middle–Late Miocene sediments of Dhemaji and Lakhimpur districts of Assam, India and its palaeoecological and palaeophytogeographical implications.** *J Earth Syst Sci* 2011, 120:681–701.

**Menéndez CA: Die fossilen floren Südamerikas.** In ***Biogeography and ecology in South America, vol. 2.* Edited by Fittkau EJ, Illies J, Klinge H, Schwabe GH, Sioli H. Hague: Springer; 1969:519**–**561.**

Menzel P: **Beitrag zur Kenntniss der Tertiärflora des Jesuitengrabens bei Kundratitz.** *Sitzungsber Abh Naturwiss Gesell Isis Dresden* 1897: 3–18.

Meschinelli A, Squinabol X: *Flora Tertiaria Italica*. Patavii: Sumptibus Auctorum Typis Seminarii, 1892:1–575.

Meyer HW, Manchester SR: **The Oligocene Bridge Creek flora of the John Day Formation, Oregon.** *Univ Calif Publ Geol Sci* 1997, 141:1–195.

Meyer HW, Smith DM: Paleontology of the Upper Eocene Florissant Formation, Colorado. *Geol Soc Amer Special Pap* 2008, 435:1–177.

Morita H: **On new species of the genera *Cinnamomum* and *Smilax* from the Miocene deposits of Oguni-Machi, Uzen Province, Japan.** *Jap J Geol Geogr* 1932, 9:1–8.

Newberry JS: **Brief descriptions of fossil plants, chiefly Tertiary, from western North America.** *Proc U S Natl Mus* 1883, 5:502–514.

Newberry JS: **Description of a species of *Bauhinia* from the Cretaceous clays of New Jersey.** *Bull Torrey Bot Club* 1886, 13:77–78.

Newberry JS: **The flora of the Amboy Clays.** *Monogr U S Geol Surv* 1895, 26:1–260.

Nury D: **Lacustrine Oligocene basins in southern Province, France.** *AAPG Stud Geol* 2000, 46:381–388.

Onuki Y: *Fossil flora of the Fushun coal-field.* Archive no. 3795, deposited at the National Geogical Archives of China, Beijing. Fushun: Fushun Middle School; 1931:1–5*.*

Owens SA, Fields PF, Ewers FW: **Degradation of the upper pulvinus in modern and fossil leaves of *Cercis* (Fabaceae).** *Amer J Bot* 1998, 85:273–284.

Paolucci L: *Nuovi Materiali e Ricerche Critiche sulle Piante Fossili Terziarie dei Gessi di Ancona.* Ancona: A. Gustavo Morelli; 1896:1–158.

Pavlyutkin BI: **The Ust-Davydovka Formation: a Paleogene reference stratigraphic unit of the Primorye region, Russian Far East.** *Russ J Pacific Geol* 2007, 1:572–585.

Pavlyutkin BI, Petrenko TI: *Stratigraphy of Paleogene-Neogene sediments in Primorye*. Vladivostok: Dalnauka; 2010:1–164.

Penhallow DP: *Report on Tertiary plants of British Columbia collected by Lawrence M. Lambe in 1906, together with a discussion of previously recorded Tertiary floras. Canada Department of Mines, Geological Survey Branch 1013.* Ottawa: Government Printing Bureau; 1908:1–167.

Pickford M, Senut B: Palaeobiology of the Albertine Rift Valley: **General conclusions and synthesis.** In *Geology and palaeobiology of the Albertine Rift Valley, Uganda-Zaire, Vol. 2: Palaeobiology*. Edited by Senut B, Pickford M. *CIFEG Occasional Publication, Orléans* 1994*,* 29:409–423.

Pole M: **The fossil flora of Melville Island, northern Australia.** *The Beagle, Records of the Museums and Art Galleries of the Northern*

*Territory* 1998, 14:1–28.

Pole M, Bowman DMJS: **Tertiary plant fossils from Australia’s ‘Top End’**. *Austral Syst Bot* 1996, 9:113–126.

Prakash U, Prasad N: **Wood of *Bauhinia* from the Siwalik beds of Uttar Pradesh, India.** *Palaeobotanist* 1984, 32:140–145.

Principi P: **Le Dicotiledoni fossili del giacimento oligocenico di Santa Giustina e Sassello in Liguria.** *Mem Serv Descrizione Carta Geol Italia* 1916, 6: 9–294.

Principi P: **La Flora Oligocenica di Chiavon e Salcedo**. *Mem Serv Descrizione Carta Geol Italia* 1926, 10:1–130.

Rásky K: **Die Oligozäne Flora des Kisceller Tons in der Umgebung von Budapest.** *Földt Közlony* 1943, 73 (4-9):503–536.

Retallack G, Dilcher DL: **A coastal hypothesis for the dispersal and rise to dominance of flowering plants.** In *Palaeobotany, Palaeoecology, and Evolution, vol. 2.* Edited by Niklas KJ. New York: Praeger; 1981:27–77.

Ridd MF, Barber AJ, Crow MJ: *The geology of Thailand*. London: Geological Society Publishing House; 2011:1–626.

Romanov MS, Dilcher DL: **Fruit structure in Magnoliaceae *s. l.* and Archaeanthus and their relationships.** *Amer Jour Bot* 2013, 100:1494–1508.

Sanyal P, Sinha R: **Evolution of the Indian summer monsoon: synthesis of continental records.** *Geolo Soc London, Special Publ* 2010, 342:153–183.

Saporta G: **Études sur la Végétation du Sud-Est de la France a L’Époque Tertiaire, 3.** *Ann Sci Nat, Sér 4 Bot* 1862, 17:191–311.

Saporta G: **Notice sur les Plantes Fossiles des Calcaires Concrétionnés de Brognon (Côte-d'Or).** *Bull Soc Géol France, Deux Sér* 1866, 23:253–283

Saporta G: **Études sur la Végétation du Sud-Est de la France a L’Époque Tertiaire, Troisième Partie 1.** *Ann Sci Nat, Sér 5 Bot* 8: 5–136.

Saporta G: **Études sur la végétation du sud-est de la France a l'époque Tertiaire. Supplément. Revision de la Flore des Gypses d’Aix. Dicotyledoneae, Apetalae.** *Ann Sci Nat, Sér 5* *Bot* 1873, 18:23–146.

Saporta G: **Les Périodes Végétales De L'Époque Tertiaire.** *La Nature* 1877, 5:403–407.

Saporta G: *Le Monde des Plantes avant L'Apparition de L'Homme.* Paris: G. Masson; 1879:1–416.

Schimper WP: *Traité de paléontologie végétale, vol. 3.* Paris: J.B. Baillière; 1874:1–896.

Schimper W, Schenk A: **Palaeophytologie.** In *Handbuch der Palaeontologie 2*. Edited by Zittel KA. München & Leipzig: Druck und Verlag von R. Oldenbourg; 1890:1–958.

Seward AC, Conway V: **Additional Cretaceous plants from western Greenland.** *Kungl Svenska Vetenskapsakad Handl* 1935,15:3–41.

Sen I, Bera S: **Petrified wood remains from the Neogene of Tripura, India.** *Geophytology* 2005, 35:65–73.

Shi GL, Zhou ZY, Xie ZM: **A new Oligocene *Calocedrus* from South China and its implications for transpacific floristic exchanges.** *Amer J Bot* 2012, 99:108–120.

Shilin PV: **On the age of plant-bearing deposits of Taldysaj.** *Vestnik Akad Nauk Kazakh* *SSR* 1970, 1:57–59.

Shilin PV: **Late Cretaceous flora of the Beleuty River.** *Mater Istorii Fauny Fl Kazakhstana* 1977, 7:126–139.

Shilin PV, Romanova EV: *Senonskie flory Kazakhstana (= Senonian flora of Kazakhstan)*. Alma-Ata: Nauka; 1978:1–176.

Smiley CJ: **The Ellensburg flora of Washington.** *Univ Calif Publ Geol Sci* 1963, 35:159–276.

Songtham W, Ratanasthien B, Mildenhall DC: **New species of algae *Actinastrum lagerheim* and *Closterium nitzsch* ex. Ralfs from middle Miocene sediments of Chiang Muan basin, Phayao, Thailand, with tropical pollen composition.** *Sci Asia* 2004, 30:171–181.

Srivastava R, Guleria JS: *A catalogue of Cenozoic (Tertiary) plant megafossils from India (1989–2005).* Lucknow: Birbal Sahni Institute of Palaeobotany; 2006:1–76.

Takhtajan AL, Vakhrameev VA, Radtschenko GP: **Gymnosperms and angiosperms.** In *Osnovy Paleontologii*, vol. 15. Edited by Orlov A. Moscow: Gosgeoltekhizdat; 1963:1–743.

Tanai T: **Late Cretaceous floras from the Kuji District, northeastern Honshu, Japan.** *J Fac Sci Hokkaido Univ, Ser 4* *Geol Mineral* 1979, 19:75–136.

Tanai T: **The revision of the so-called "*Cercidiphyllum*" leaves from the Paleogene of North Japan.** *J Fac Sci Hokkaido Univ, Ser 4* *Geol Mineral* 1981, 19: 451–484.

Tao JR, Xiong XZ: **The latest Cretaceous flora of Heilongjiang Province and the floristic relationship between East Asia and North America.** *Acta Phytotax Sin* 1986, 24:1–15, 121–135.

Tao JR, Zhou ZK, Liu YS: *The evolution of the Late Cretaceous–Cenozoic floras in China.* Beijing: Science Press; 2000:1–282.

Trivedi BS, Panjwani M: **Fossil wood of *Bauhinia* from the Siwalik beds of the Kalagarh, U. P.** *Geophytology* 1986, 16:66–69.

Unger F: *Genera et Species Plantarum Fossilium*. Vindobona: Apud Wilhelmum Braumüller; 1850:1–627.

Unger F: **Sylloge Plantarum Fossilium.** *Denkschr Kaiserl Akad Wiss Wien, Math-Naturwiss Kl* 1864, 22:1–36.

Unger F: *Die Fossile Flora von Kumi auf der Insel Euboea.* Wien: Aus der Kaiserlich-Königlichen Hof- und Staatsdruckerei; 1867:3–66.

Usnadze MD: **Rastitelnye Ostsatki iz Kontinentalnyh Tretichnyh Otlozhenii Severnogo Priaralja (=Vegetative remains from the continental Tertiary deposits of northern Priaralye).** In *Sbornik Pamjati A. N. Kryshtofovicha*. Edited by Komarova BL. Moscow: Akademii Nauk SSSR; 1957:213–233.

Usnadze MD: **A contribution to the nomenclature of the Tertiary species of *Cercis*.** *Bot Zhurn* 1971, 56:970.

Vakhrameyev VA: **The Late Cretaceous florae of the USSR Pacific coast, their stratigraphic range and peculiarities of composition.** *Proc USSR Acad Sci, Geol Ser* 1966, 3:76–87.

Velenovský J: **Die Flora der Böhmischen Kreideformation 4.** *Beitr Paläontol Österreich-Ungarns* 1885, 5: 1–14.

Wang Q: **Nomenclatural notes on *Leguminosites* and several taxonomically relevant names (fossil Leguminosae).** *Taxon* 2012a, 61:871–877.

Wang Q: **Pulvini of *Cercis* leaves from the Miocene Shanwang Formation of Shandong Province and the early evolution of the pulvinus in Leguminosae.** *Acta Palaeontol Sin* 2012b, 51:1–13.

Wang Q: **Fruits of *Hemitrapa* (Trapaceae) from the Miocene of eastern China, their correlation with *Sporotrapoidites erdtmanii* pollen and paleobiogeographic implications.** *J Paleontol* 2012c, 86:156–166.

Wang Q, Manchester SR, Dilcher DL: **Fruits and foliage of *Pueraria* (Leguminosae, Papilionoideae) from the Neogene of Eurasia and their biogeographic implications.** *Amer J Bot* 2010, 97:1982–1998.

Wang Q, Manchester SR, Gregor H-J, Shen S, Li ZY: **Fruits of *Koelreuteria* (Sapindaceae) from the Cenozoic throughout the Northern Hemisphere: their ecological, evolutionary and biogeographic implications.** *Amer J Bot* 2013, 100:422–449.

Watelet A: *Description des Plantes Fossiles du Bassin de Paris.* Paris: J. B. Baillière et Fils; 1866:1–264.

WGCPC [abbreviation for "*Cenozoic plants from China*" Writing Group of Institute of Botany and Nanjing Institute of Geology and Palaeontology, Academia Sinica]: *Fossil Plants of China, 3 Cenozoic Plants from China*. Beijing: Science Press; 1978:1–232.

Wilf P, Johnson KR, Cúneo NR, Smith ME, Singer BS, Gandolfo MA: **Eocene Plant Diversity at Laguna del Hunco and Río Pichileufú, Patagonia, Argentina.** *Amer Nat* 2005, 165:634–650.

Willis B: **Age of the Bugishu sandstone on physiographic evidence.** *J Geol* 1933, 41:699–701.

Zhilin SG, Löve D, Cronquist A, Takhtajan A: **History of the development of the temperate forest flora in Kazakhstan, U. S. S. R. from the Oligocene to the early Miocene.** *Bot Rev* 1989, 55:205–330.
